# LG-Transformer: learned-graph transformer framework enabling diverse physicochemical properties prediction toward fuel design

**DOI:** 10.1038/s41467-026-73853-z

**Published:** 2026-06-03

**Authors:** Jiabo Zhang, Xiang Lv, Hui An, Jing Li, Peng Han, Zhen Huang

**Affiliations:** 1 Key Laboratory for Power Machinery and Engineering, Shanghai Jiao Tong University, Shanghai, China; 2 Department of Computer Science, University of Electronic Science and Technology of China, Chengdu, China; 3 Harbin Institute of Technology (Shenzhen), Shenzhen, China; 4 School of Software Engineering, Faculty of Electronic and Information Engineering, Xi’an Jiaotong University, Xi’an, China

**Keywords:** Fossil fuels, Computer science

## Abstract

Green fuels are essential for decarbonizing transportation sectors, requiring accurate prediction of different physicochemical properties to optimize engine performance and emissions. Although artificial intelligence-based models demonstrate significant potential to accelerate fuel design, most existing methods cannot utilize the internal and external information within and between fuel molecules with interpretability, limiting their generalizability for diverse properties prediction. To address these challenges, a deep learning framework, the learned graph feature fusion Transformer (LG-Transformer), is proposed. Unlike conventional graph neural networks (GNNs) that operate on atom-bond molecular graphs, LG-Transformer employs contrastive learning to construct an inter-molecular relationship graph guided by topological descriptors and property similarity, enabling property-aware feature propagation through Transformer layers for various property prediction. Supporting this effort, a comprehensive fuel property database is developed, containing 1850 diverse molecules across 26 chemical classes, each annotated with 17 key physicochemical properties relevant to engine performance. Here we show that LG-Transformer achieves superior predictive performance with an average *R*^2^ of 0.900, significantly outperforming other GNN and deep learning baselines. Additionally, interpretability analyses via integrated gradients reveal underlying molecular structure-property relationships.

## Abstract (web summary)

Green fuel design requires accurate and generalizable prediction of multiple properties across diverse molecules. Here, the authors develop a comprehensive fuel database and an interpretable LG-Transformer model to achieve high accuracy fuel property prediction.

## Introduction

The global transition to low-carbon energy is reshaping the transportation sector, with significant progress in the electrification of light-duty vehicles driven by advances in electric motors and battery technologies. However, combustion engines maintain the primary power source for heavy-duty vehicles, maritime shipping, and aviation, which together account for over 40% of transportation-related CO_2_ emissions^1–4^. Decarbonizing these sectors is critical for achieving net-zero targets, and low-carbon green fuels, such as hydrogen-based e-fuels and sustainable aviation fuels (SAFs), hold significant potential. For instance, the United States Department of Energy launched the co-optimization of fuels and engines (Co-Optima) initiative in 2016 to co-develop advanced green fuels and engines to achieve 60% thermal efficiency and reduce pollutant emissions by 50%^5^. The European Union introduced the ReFuelEU Aviation regulation, mandating a gradual increase in the use of SAFs to achieve a 55% reduction in aviation emissions by 2030^6^. Furthermore, Japan has established the Green Innovation Fund, which focuses on supporting the research and development of synthetic e-fuels. These coordinated efforts highlight the critical role of fuel innovation in achieving sustainable transportation.

Nevertheless, the development of these fuels is hindered by the need to balance combustion performance with engine compatibility, leading to long development cycles and delayed integration into existing infrastructure^7,8^. Conventional fuel development follows a passive, trial-and-error approach, in which candidate components are physically blended and tested to evaluate engine performance and emissions^9–15^. Although such empirical approaches have yielded valuable insights, it is limited by the vast combinatorial complexity of fuel mixtures, making exhaustive experimental screening time- and cost-prohibitive. To overcome these limitations, property-driven fuel design has emerged as a promising alternative. By linking key physicochemical properties of green fuels (e.g., cetane number (CN), derived cetane number (DCN), research octane number (RON), motor octane number (MON), viscosity, flash point (FP), boiling point (BP), among others) to engine combustion and emission behavior^5,16^, this approach enables efficient screening, reduces development cycles, and improves engine compatibility.

To support this methodological shift, computational prediction of fuel properties from molecular structure has become an essential tool. This field has progressed from early group additivity methods^17^ to machine learning (ML) models integrated with quantitative structure-property relationship (QSPR) analysis. These methods employ feature selection techniques to filter out irrelevant descriptors and identify key physicochemical features^18^, enabling more accurate property predictions through data-driven training. For example, various ML algorithms, such as random forest (RF), artificial neural networks (ANN), lightGBM (LGBM), support vector regression (SVR), and gradient boosting methods like CatBoost, have been widely applied to predict key fuel properties including CN^19^, MON^20^, RON^20^, viscosity^21^, MP^22^, standard enthalpy of formation (SEF)^23^, and heat of vaporization (HOV)^24^. More recently, with the advancement of deep learning, specialized architectures including convolutional neural networks (CNNs), recurrent neural networks (RNNs), and graph neural networks (GNNs) have demonstrated enhanced capabilities in predicting the above key physicochemical properties^25–28^. Despite these advances, these methods still exhibit critical limitations for practical applications. Traditional ML frameworks depend heavily on manually engineered molecular descriptors, which restrict their generalizability across a wide spectrum of fuel chemistries and limit their applicability to complex transportation fuels. Moreover, engine performance is governed by the coupled effects of multiple fuel properties, yet most models do not explicitly utilize the relationship between molecule descriptors and properties, which limits their applicability for predicting a broad range of properties in practical fuel design and optimization.

To address these limitations, advanced deep learning architectures capable of capturing both complex molecular features and different physicochemical properties prediction are urgently needed. The Transformer architecture, built on the self-attention mechanism^29^, offers a promising solution. Originally developed for natural language processing, Transformer architectures have demonstrated broad applicability across various scientific domains, including biology^30^, medicine^31^, and quantum computing^32^, enabling breakthroughs in tasks, such as protein folding, clinical diagnosis, and quantum error correction. In the field of molecule property prediction, Khambhawala et al.^33^ pioneered the application of Transformer architectures by employing simplified molecular input line entry system (SMILES) strings to predict the melting point (MP) of ionic liquids (ILs). Their approach involved pretraining the model on a large-scale dataset of 1.8 billion molecules to enhance molecular feature extraction, followed by fine-tuning on a task-specific IL dataset to improve prediction accuracy. However, the direct application of the Transformer to fuel design faces two fundamental challenges. First, text-based SMILES strings omit essential chemical and topological information, necessitating extensive pretraining on large-scale datasets to recover molecular context. Second, even with richer molecular descriptors, standard Transformers often treat them as unordered inputs, neglecting intrinsic physicochemical relationships and leading to inconsistent predictions. These limitations highlight the need for architectural improvements to traditional Transformer models in fuel design. One promising direction is to introduce a relational inductive bias into representation learning by integrating contrastive learning with a graph convolutional network (GCN). This approach can effectively capture molecular topology and encode chemically meaningful representations^34,35^. When combined with attention mechanisms, such hybrid models may better preserve structure-property relationships and facilitate accurate prediction of diverse physicochemical properties. However, the development of robust frameworks applied for predicting different properties remains constrained by the lack of standardized fuel datasets that incorporate multicomponent classifications and multidimensional property annotations.

Motivated by the limitations identified above, the objectives of the present study are three-fold: (1) to construct a comprehensive fuel property dataset including various key physicochemical attributes across representative molecular classes; (2) to develop a general framework applied for predicting various properties, which employs a learned-graph feature fusion module for descriptor-level fusion, combined with a Transformer-based attention mechanism to capture the higher-order dependencies among the fused features; and (3) to employ interpretability tools to assess the contribution of different molecular descriptors to each property prediction. To this end, a comprehensive database for predicting diverse properties fuel design is constructed, including 1850 fuels across 26 fuel categories and annotated with 17 key physicochemical properties relevant to engine performance. Based on this dataset, a learned-graph Transformer (LG-Transformer) framework is proposed. Unlike conventional GNNs that operate on molecular structural graphs, the “graph” in this work represents an inter-molecular relationship network built upon property similarity learned through contrastive learning. This enables the model to capture cross-molecular correlations in property space, marking a fundamental distinction and core contribution of this study. This innovative framework first learns structured, property-aware embeddings from molecular descriptors via a GCN feature propagation process based on contrastive learning. A subsequent Transformer module then models the high-order dependencies among these fused features to perform diverse properties prediction. In addition, the integrated gradients (IG)^36^ method is used to quantify the impact of different descriptor types on property predictions. The experimental results demonstrated that LG-Transformer achieves state-of-the-art performance, offering a powerful and computationally efficient tool to accelerate the virtual screening and rational design of next-generation green fuels.

## Results

### Dataset construction for predicting diverse fuel properties

Fuel design requires consideration of diverse physicochemical properties, as fuel performance in engines depends on complex and coupled property interactions. However, most existing studies focus on predicting a single property or a limited subset, often neglecting other properties that are essential for practical engine applications. To address this gap, the present work constructs a comprehensive dataset by aggregating data from a wide range of literature sources and chemical property databases^23,24,37–67^, as listed in Table 1. The dataset contains 1850 fuel molecules spanning 26 categories with 17 key physicochemical properties. All data included are collected exclusively from experimental measurements, with any predicted or estimated values strictly excluded to ensure data authenticity and reliability. The source of each experimental value has been clearly annotated on Figshare (see Data availability). Moreover, to facilitate reproducibility and provide comprehensive dataset information, Table 1 summarizes the units, test methods, and standards, number of data points, and representative measurement uncertainties for all properties. The selected key properties, grouped into physical and chemical properties, are detailed below.

**Table 1:** Units, measurement standards, data counts, and uncertainties for physical and chemical properties

	Properties	Units	Test methods or standards	Train data	Test data	Uncertainties
Physical Properties	Viscosity	mPa ⋅ s	ASTM D445^55^	253	63	± 0.35% ^75^
	EOV	kJ mol^−1^	Calorimetry (25^∘^C)^55^	442	110	± 0.5 kJ mol^−1^^76^
	SEF	kJ mol^−1^	Calorimetry (25^∘^C)^55^	269	67	± 5.0 kJ mol^−1^^23^
	VP	kPa	Reid Method (25^∘^C)^55^	555	139	± 5.5 kPa ^77^
	ST	dyne/cm	Ring Method (25^∘^C)^63^	416	104	± 0.9 dyne/cm ^78^
	FP	^∘^C	ASTM D93^55^	843	211	± 5^∘^C ^79^
	Density	g cm^−3^	Densimetry (15^∘^C to 30^∘^C)^55^	950	237	± 0.0005 g cm^−3^^101^
	BP	^∘^C	ASTM D86^55^	1007	252	± 2. 0^∘^C ^80^
	MP	^∘^C	Capillary Tube Method^55^	854	214	± 0. 5^∘^C ^81^
Chemical Properties	RON	–	ASTM D2699^72^	307	77	± 1.0 ^72^
	MON	–	ASTM D2700^73^	277	69	± 1.0 ^73^
	CN	–	ASTM D613^74^	510	128	± 2.5 ^74^
	DCN	–	ASTM D6890^53^	142	36	± 2.0 ^53^
	LHV	kJ mol^−1^	Bomb Calorimetry^58^	839	210	± 0.2% ^82^
	UFL	vol%	ASTM E681, E682^58^	462	115	± 0.5vol% ^58^
	LFL	vol%	ASTM E681, E682^58^	650	163	± 0.5vol% ^58^
	YSI	–	Color-Ratio Pyrometry^54^	354	89	${{{\rm{MAX}}}}(\pm 4.0,3.0\%)$^54^

All data are collected from standardized experiments to ensure reliability and consistency. *EOV* enthalpy of vaporization, *SEF* enthalpy of formation, *VP* vapor pressure, *ST* surface tension, *FP* flash point, *BP* boiling point, *MP* melting point, *RON* research octane number, *MON* motor octane number, *CN* cetane number, *DCN* derived cetane number, *LHV* lower heating value, *UFL* upper flammability limit, *LFL* lower flammability limit, *YSI* yield sooting index, *ASTM* American Society for Testing and Materials.

Physical properties: the physical properties of fuels play a critical role in the injection, atomization, droplet breakup, and vaporization processes. To systematically capture these effects, the present dataset includes nine key physical properties, namely BP, MP, vapor pressure (VP), density, surface tension (ST), viscosity, FP, SEF, and enthalpy of vaporization (EOV)^23,42,55,63–67^. Specifically, BP, MP, and VP primarily influence fuel injection characteristics^68^. Density, ST, and viscosity affect atomization performance, with viscosity being especially important for droplet breakup^68,69^. FP and EOV are selected as they are important for fuel vaporization behavior^68^, and SEF is included due to its fundamental role in characterizing combustion energy release and thermodynamic efficiency^64^. As summarized in Table 1, all measurements are collected under standardized conditions, with detailed test methods, temperatures, and representative uncertainties. Specifically, VP is measured at 25^∘^C using the Reid method^55^, SEF and EOV are obtained at 25^∘^C using the calorimetry method^55^, ST is measured at 25^∘^C using the Ring method^63^, density is measured between 15^∘^C to 30^∘^C using digital densimetry^55^, viscosity is measured at 20^∘^C following ASTM D445^55^, FP is determined using the Pensky-Martens closed-cup test^55^, BP is obtained via ASTM D86^55^, and MP uses the capillary-tube method^55^. Representative measurement uncertainties for each property are compiled from the sources or standardized testing methods and summarized in Table 1.

Chemical properties: Eight chemical properties of fuels that primarily influence combustion behavior and emission characteristics in engines are selected in the current dataset. Specifically, RON and MON reflect the anti-knock performance of fuels, with higher octane numbers enabling the use of higher engine compression ratios^39,70^. CN and DCN indicate ignition quality, including self-ignition tendency and ignition delay^39,71^. Yield sooting index (YSI) represents the sooting propensity of a fuel, which directly correlates with particulate emissions^54^. Lower heating value (LHV) describes the heat released per unit mass of fuel during complete combustion^61^. Upper flammability limit (UFL) and lower flammability limit (LFL) refer to the maximum and minimum volume concentration of combustible gases that can sustain combustion in air^56,57,59,60^. Similarly, as listed in Table 1, RON, MON, CN, DCN, UFL, and LFL are measured according to ASTM or equivalent standardized methods^53,58,72–74^. LHV is determined via bomb calorimetry^58^, and YSI is measured using color-ratio pyrometry^54^. Representative uncertainties for each property are compiled from the sources or standardized testing methods^23,53–55,58,72–82^ and summarized in Table 1, and more details are in the Methods section. These standardized protocols and uncertainty assessments ensure that the above property data are consistent, reliable, and traceable across the dataset.

**Fig. 1:**
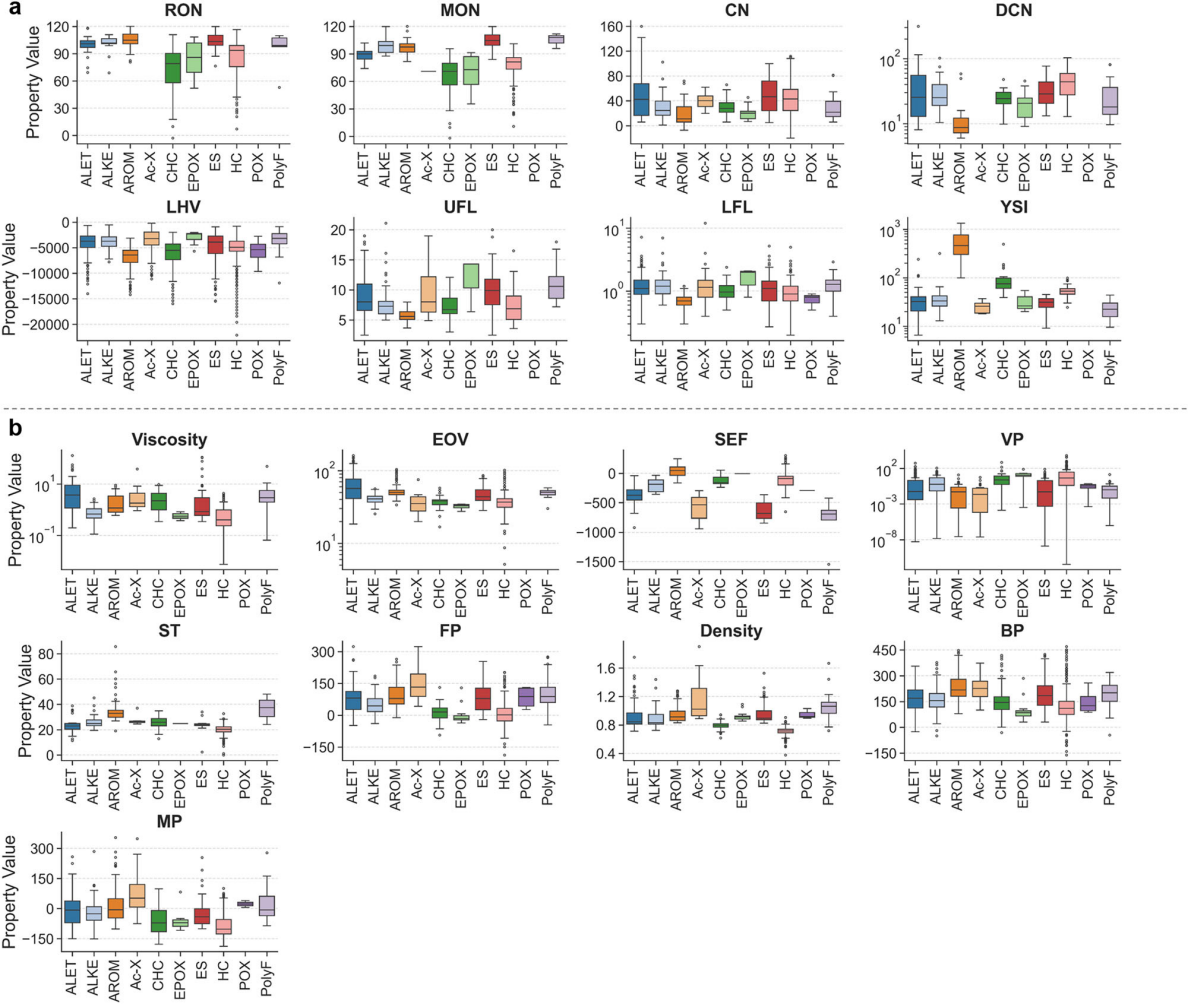
Distribution of fuel properties across different chemical classes in the database. The figure displays box plots for the value distributions of 17 key physicochemical properties across ten major fuel classes. The properties are grouped into Physical Properties (panel **a**) and Chemical Properties (panel **b**). For each box plot, the central line indicates the median, the box limits represent the upper and lower quartiles (the interquartile range, IQR), and the whiskers extend to 1.5 times the IQR from the box limits. Data points beyond the whiskers are shown as individual outliers. The visualization highlights the variance in property values both within and between different fuel classes. Sample sizes for each class are provided in Supplementary Tab. 2. Source data are provided as a Source Data file. (EOV enthalpy of vaporization, SEF enthalpy of formation, VP vapor pressure, ST surface tension, FP flash point, BP boiling point, MP melting point, RON research octane number, MON motor octane number, CN cetane number, DCN derived cetane number, LHV lower heating value, UFL upper flammability limit, LFL lower flammability limit, YSI yield sooting index. HC Hydrocarbons, CHC Cyclic hydrocarbons, AROM Aromatics, ALET Alcohols & Ethers, ES Esters, ALKE Aldehydes & Ketones, Ac-X Acids & Derivatives, PolyF Polyfunctionals, POX Peroxides, EPOX Epoxides).

On the other hand, to improve chemical representation and model generalization, the original 26 compound categories are consolidated into 10 major groups, containing hydrocarbons (HC), cyclic hydrocarbons (CHC), aromatics (AROM), alcohols and ethers (ALET), esters (ES), aldehydes and ketones (ALKE), acids and derivatives (Ac-X), polyfunctionals (PolyF), peroxides (POX), and epoxides (EPOX). The 17 key physicochemical properties are visualized in Fig. 1, which illustrates their distributions across the major compound groups. Supplementary Tab. 2 further summarizes the number of fuel molecules in each of the 26 chemical families across the 17 properties. It is readily observed that some fuel classes, such as aromatics and alkenes, are widely used in engines as fuel and therefore have extensive measurements, while other classes, such as bicycloalkanes and terpenes, are less common and contain fewer data points. In constructing the database, efforts are made to maximize coverage, and the resulting collection represents, to our knowledge, the most comprehensive dataset of engine-relevant fuels currently available. Despite these differences in data availability across fuel classes, the proposed modeling approach achieves strong predictive performance for all properties, which will be discussed in the following sections. Moreover, an open-access website, https://ai4fuel.sjtu.edu.cn/, has been constructed, which provides a complete dataset to efficiently search and retrieve property information.

### Framework of proposed LG-Transformer

**Fig. 2:**
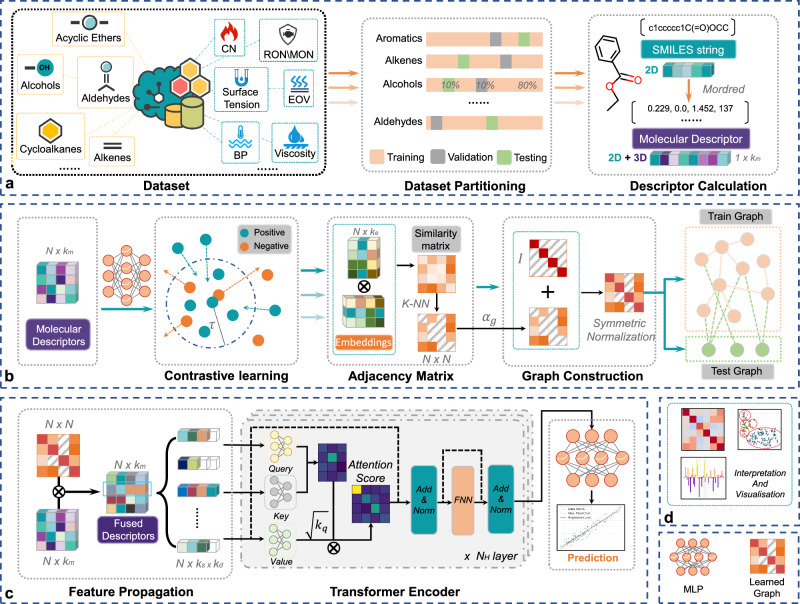
Schematic of the LG-Transformer model for fuel property prediction. **a** Dataset preparation. The database comprises 1850 compounds across 26 fuel classes with 17 physicochemical properties. Data is partitioned into training (80%) and testing (20%) sets, stratified by class. Initial 2D and 3D molecular descriptors are calculated from SMILES (simplified molecular input line entry system) strings using the Mordred toolkit. **b** Property-aware graph construction. A multi-layer perceptron (MLP)-based contrastive learning framework is employed to generate molecular embeddings. Positive and negative sample pairs are defined based on property similarity (threshold *τ*). A *k*-Nearest Neighbor (*k*-NN) graph is constructed from the resulting embedding similarity matrix and then symmetrically normalized (with neighbor weight *α*_g_) to form the final learned graph structure. **c** Graph-based feature fusion and prediction. The original descriptors undergo feature propagation on the learned graph to produce fused features. These features, grouped by descriptor type, are fed into a Transformer Encoder to capture inter-feature relationships via an attention mechanism. A final MLP performs regression to predict the target fuel property. **d** Model interpretation. The efficacy of the feature fusion process is assessed through visualization. Model interpretability is achieved by combining attention scores with descriptor contributions calculated using IG, providing insights into the prediction mechanism. (CN cetane number, RON research octane number, MON motor octane number, EOV enthalpy of vaporization, BP boiling point).

From Table 1, the 17 fuel properties show weak mutual correlations and correspond to distinct physical and chemical behaviors. Therefore, rather than employing an MTL framework^83,84^, each property is predicted independently. This design enables the development of a robust and generalizable single-task architecture that can be applied consistently across diverse fuel properties, while still supporting an integrated fuel design workflow through the combination of individually validated predictions. Figure 2 presents the overall framework of LG-Transformer for fuel property prediction, outlining the complete pipeline from dataset construction to model architecture and interpretation. Specifically, Fig. 2a shows the data preparation process. Note that, for each specific property dataset, five-fold cross-validation is performed using stratified sampling across different fuel types, with training and validation conducted independently for each property dataset. For each molecule, a comprehensive set of molecular descriptors, including both 2D and 3D structural information, is extracted from its SMILES string using Mordred^85^, forming the initial feature vector. A full list of descriptor groups is provided in Supplementary Tab. 1.

Moreover, Fig. 2b, c depicts the architecture of the LG-Transformer framework, which operates in two main stages as detailed in the Methods section. The first stage constructs a property-aware molecular graph through contrastive learning, while the second stage performs GCN feature propagation followed by a Transformer encoder to capture complex dependencies, and could be applied to different properties. Specifically, in the first stage, shown in Fig. 2b, a graph is learned to encode molecular relationships centered on fuel properties. A contrastive learning task is used to pretrain a multilayer perceptron (MLP) embedding network on molecular descriptors. Molecular similarity is defined based on property values, where positive pairs share similar properties and negative pairs are dissimilar in properties but close in the original feature space. This training process drives molecules with similar physicochemical behavior toward proximity in the latent embedding space. Embedding quality is assessed by calculating Spearman correlation with the corresponding property values. A similarity matrix is constructed through dot product, then sparsified using the *k*-nearest neighbors (KNN) algorithm. To obtain the final graph propagation matrix, the initial matrix is scaled by a tunable weight *α*_g_, augmented with self-loops by adding the identity matrix, and symmetrically normalized. This matrix serves as the learned molecular relationship graph.

Although the embeddings generated in the first stage effectively define the relational graph, the original molecular descriptors retain a more comprehensive set of physicochemical features. Therefore, the second stage, illustrated in Fig. 2c, utilizes the learned graph to refine these initial descriptors through a GCN feature propagation process. Each molecular feature vector is updated by aggregating information from neighboring molecules defined by the property-centric graph, yielding context-aware descriptors that incorporate both intrinsic molecular characteristics and relational context. These enriched descriptors are grouped and structured as an input sequence for the Transformer module. The self-attention mechanism within the Transformer captures long-range dependencies among descriptor groups, and a final MLP maps the encoded representation to property predictions. To enhance interpretability, the framework includes a model interpretation module, as shown in Fig. 2d, which quantifies the contributions of descriptor groups and attention pathways to the final predictions.

### Performance comparison between the LG-Transformer and baseline models on fuel property prediction

LG-Transformer shows the best performance in predicting all fuel properties. To validate the performance of the proposed LG-Transformer framework, a comprehensive comparative analysis is conducted against a diverse set of baseline models spanning four categories. The first category includes traditional machine learning models commonly used in fuel design, such as SVR^23^, MLP^23^, ML-QSPR^86^, and the gradient boosting model CatBoost^24^. The second category incorporates conventional deep learning architectures operating on non-graph molecular representations, specifically a SMILES-based CNN^87^ and a vanilla Transformer^29^. The third category evaluates representative graph-based molecular property prediction models^83,84,88^, including Chemprop^88^ and AttentiveFP^84^. The final category adds state-of-the-art contrastive learning methods, MolCLR^89^ and KANO^90^, to ensure a rigorous comparison against modern molecular representation learning approaches. To ensure a fair comparison, all models are evaluated under the same experimental conditions. First, an identical dataset is used for all methods. A five-fold stratified cross-validation scheme is employed to enhance the reliability and statistical stability of the evaluation, where the dataset is split into five equal folds randomly. In each iteration, four folds are combined for model training and validation, while the remaining fold is reserved for testing. This procedure is repeated for five iterations, ensuring that each fold serves exactly once as the test set. This approach effectively reduces overfitting risks and provides a more robust assessment of model generalizability compared to a single random split. Supplementary Tabs. 5 and 7 provides all the experimental setups. Second, for every baseline model, an independent Bayesian hyperparameter search is performed using Optuna^91^ within the respective search spaces suggested by their original literature, ensuring that all compared models achieve their optimal performance. Predictive performance for all target properties is evaluated using the coefficient of determination (*R*^2^). The detailed comparative results are summarized in Table 2. Additionally, regression fit scatter plots for all properties are provided in the Supplementary Figs. 4 and 5, and the hyperparameter search space is specified in Supplementary Tables. 4 and 6.

**Table 2:** Performance comparison of the benchmark models (*R*^2^ coefficients)

	Dataset	SVR^23^	MLP^23^	CatBoost^24^	Transformer^29^	CNN^87^	ML-QSPR^86^	Chemprop^88^	AttentiveFP^84^	MolCLR^89^	KANO^90^	LG-Transformer
Physical Properties	Viscosity	0.851 ± 0.049	0.835 ± 0.053	0.829 ± 0.049	0.854 ± 0.037	0.849 ± 0.049	0.835 ± 0.033	0.859 ± 0.033	0.869 ± 0.043	0.858 ± 0.044	0.875 ± 0.029	**0.894** ± **0.024**
	EOV	0.963 ± 0.021	0.958 ± 0.021	0.951 ± 0.020	0.962 ± 0.015	0.953 ± 0.014	0.965 ± 0.009	0.952 ± 0.018	0.967 ± 0.025	0.944 ± 0.027	0.952 ± 0.020	**0.979** ± **0.003**
	SEF	0.919 ± 0.055	0.917 ± 0.058	0.926 ± 0.054	0.922 ± 0.055	0.900 ± 0.065	0.909 ± 0.052	0.922 ± 0.056	0.929 ± 0.050	0.925 ± 0.049	0.928 ± 0.051	**0.940** ± **0.044**
	VP	0.884 ± 0.049	0.876 ± 0.049	0.866 ± 0.042	0.890 ± 0.039	0.856 ± 0.023	0.837 ± 0.067	0.891 ± 0.033	0.912 ± 0.054	0.883 ± 0.032	0.882 ± 0.040	**0.928** ± **0.070**
	ST	0.793 ± 0.114	0.802 ± 0.114	0.792 ± 0.136	0.805 ± 0.097	0.706 ± 0.145	0.732 ± 0.088	0.780 ± 0.096	0.799 ± 0.132	0.817 ± 0.125	0.790 ± 0.132	**0.835** ± **0.133**
	FP	0.928 ± 0.016	0.928 ± 0.016	0.922 ± 0.025	0.905 ± 0.031	0.881 ± 0.042	0.919 ± 0.030	0.911 ± 0.030	0.927 ± 0.022	0.928 ± 0.022	0.910 ± 0.021	**0.931** ± **0.020**
	Density	0.877 ± 0.033	0.934 ± 0.033	0.931 ± 0.020	0.926 ± 0.020	0.902 ± 0.015	0.867 ± 0.012	0.930 ± 0.012	0.934 ± 0.016	0.929 ± 0.019	0.919 ± 0.019	**0.947** ± **0.017**
	BP	0.835 ± 0.017	0.835 ± 0.017	0.836 ± 0.017	0.829 ± 0.017	0.800 ± 0.044	0.802 ± 0.027	0.812 ± 0.023	0.839 ± 0.012	0.814 ± 0.026	0.814 ± 0.026	**0.852** ± **0.030**
	MP	0.772 ± 0.039	0.768 ± 0.028	0.773 ± 0.035	0.759 ± 0.017	0.736 ± 0.028	0.748 ± 0.029	0.757 ± 0.030	0.772 ± 0.029	0.757 ± 0.020	0.772 ± 0.025	**0.796** ± **0.044**
Chemical Properties	RON	0.780 ± 0.058	0.796 ± 0.058	0.790 ± 0.039	0.815 ± 0.035	0.751 ± 0.060	0.779 ± 0.034	0.794 ± 0.041	0.786 ± 0.057	0.805 ± 0.050	0.822 ± 0.040	**0.856** ± **0.038**
	MON	0.737 ± 0.024	0.736 ± 0.024	0.764 ± 0.055	0.741 ± 0.049	0.727 ± 0.044	0.731 ± 0.032	0.780 ± 0.026	0.778 ± 0.028	0.761 ± 0.035	0.770 ± 0.024	**0.807** ± **0.042**
	CN	0.768 ± 0.038	0.787 ± 0.038	0.806 ± 0.057	0.792 ± 0.039	0.724 ± 0.108	0.758 ± 0.056	0.795 ± 0.048	0.782 ± 0.067	0.798 ± 0.032	0.806 ± 0.047	**0.843** ± **0.047**
	DCN	0.763 ± 0.077	0.733 ± 0.077	0.739 ± 0.056	0.768 ± 0.066	0.705 ± 0.099	0.654 ± 0.117	0.804 ± 0.085	0.826 ± 0.073	0.768 ± 0.072	0.830 ± 0.076	**0.842** ± **0.066**
	LHV	0.994 ± 0.002	0.993 ± 0.002	0.991 ± 0.004	0.993 ± 0.002	0.983 ± 0.007	0.993 ± 0.003	0.989 ± 0.006	0.994 ± 0.001	0.991 ± 0.002	0.993 ± 0.003	**0.995** ± **0.001**
	UFL	0.855 ± 0.030	0.854 ± 0.030	0.855 ± 0.029	0.869 ± 0.025	0.851 ± 0.020	0.850 ± 0.014	0.864 ± 0.018	0.887 ± 0.031	0.871 ± 0.027	0.873 ± 0.047	**0.917** ± **0.013**
	LFL	0.890 ± 0.054	0.890 ± 0.054	0.856 ± 0.052	0.869 ± 0.043	0.829 ± 0.079	0.788 ± 0.075	0.849 ± 0.042	0.914 ± 0.054	0.860 ± 0.036	0.896 ± 0.032	**0.953** ± **0.013**
	YSI	0.952 ± 0.018	0.956 ± 0.018	0.962 ± 0.006	0.959 ± 0.019	0.946 ± 0.023	0.956 ± 0.020	0.963 ± 0.013	0.973 ± 0.014	0.946 ± 0.006	0.968 ± 0.009	**0.982** ± **0.005**
MEAN		0.857 ± 0.041	0.859 ± 0.041	0.858 ± 0.041	0.862 ± 0.036	0.829 ± 0.051	0.831 ± 0.041	0.862 ± 0.036	0.876 ± 0.042	0.862 ± 0.037	0.871 ± 0.038	**0.900** ± **0.036**

The table evaluates predictive accuracy for key fuel physicochemical properties across our model and ten representative models, including traditional machine learning methods (SVR, MLP, CatBoost, ML-QSPR), deep learning models using non-graph representations (CNN based on SMILES, Transformer), graph-based approaches (Chemprop, AttentiveFP), and contrastive learning frameworks (MolCLR, KANO). Results are reported as the average test *R*^2^ scores from 5-fold cross-validation. Our proposed LG-Transformer consistently achieves the best performance across all properties. (*EOV* enthalpy of vaporization, *SEF* enthalpy of formation, *VP* vapor pressure, *ST* surface tension, *FP* flash point, *BP* boiling point, *MP* melting point, *RON* research octane number, *MON* motor octane number, *CN* cetane number, *DCN* derived cetane number, *LHV* lower heating value, *UFL* upper flammability limit, *LFL* lower flammability limit, *YSI* yield sooting index.).

^*^ The best-performing results are marked in bold.

Overall, the proposed LG-Transformer achieves state-of-the-art performance with a mean *R*^2^ score of 0.900. This represents a performance improvement of 5% over the best-performing traditional machine learning model CatBoost (*R*^2^ = 0.858), and 4.4% over the vanilla Transformer (*R*^2^ = 0.862). Specifically, the traditional ML methods, including CatBoost, MLP, and SVR, exhibit inconsistent performance across different properties, with significant drops in accuracy for challenging tasks, such as viscosity, RON, and DCN. This limitation arises from their reliance on manually designed molecular descriptors, which cannot fully capture the complex and non-linear relationships between molecular structure and fuel properties. Graph-based methods, such as Chemprop (*R*^2^ = 0.862) and AttentiveFP (*R*^2^ = 0.876), achieve better overall performance but remain inferior to LG-Transformer on several key properties, including ST, RON, CN, LFL, and UFL. Similarly, contrastive learning models, such as MolCLR (*R*^2^ = 0.862) and KANO (*R*^2^ = 0.871), outperform traditional methods but still fall short. These approaches focus mainly on molecular connectivity or global structural similarity and fail to fully capture inter-molecular topology. In contrast, by integrating atomic-level, topological, and global molecular features, LG-Transformer effectively models complex structure-property relationships, providing consistent and reliable predictions across all fuel properties.

**Fig. 3:**
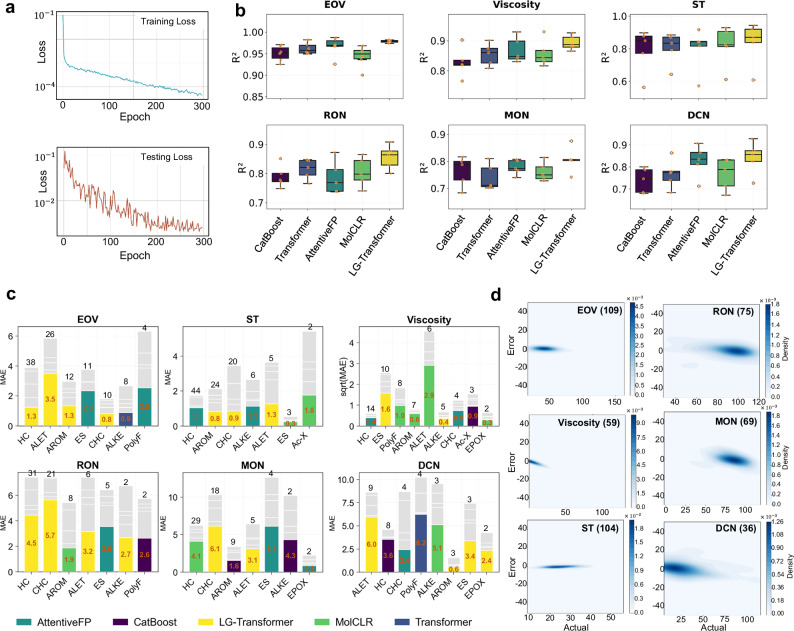
Analysis of key property prediction errors. The figure illustrates the predictive performance of LG-Transformer against four baseline models (CatBoost^24^, Transformer^29^, AttentiveFP^84^, MolCLR^89^) on six key fuel properties. **a** Training and testing loss curves for the LG-Transformer on the MON prediction task. The curves demonstrate the model’s rapid convergence and good generalization ability without significant overfitting. **b** Box plots of the *R*^2^ distributions across 5-fold cross-validation for LG-Transformer and baseline models on six key fuel properties. For each box plot, the central line indicates the median, the box limits represent the upper and lower quartiles (the interquartile range, IQR), and the whiskers extend to 1.5 times the IQR from the box limits. Data points beyond the whiskers are shown as individual outliers. The plots demonstrate that LG-Transformer achieves higher median *R*^2^ values, tighter IQRs (reflecting greater consistency), and fewer outlier points compared to other models. These results underscore the enhanced robustness and reliability of the proposed framework in predicting diverse fuel properties. **c** Comparison of model prediction errors across fuel classes and properties using ranked stacked bar charts. Each bar corresponds to a fuel class, with the sample size annotated at the top. Segments within each bar are stacked in ascending order of Mean Absolute Error (MAE), where the bottom segment (optimal model) is shown in color with its MAE value labeled, and upper segments (suboptimal models) are rendered in gray. Note that Viscosity is displayed using $\sqrt{{{{\rm{MAE}}}}}$ scaling for visual clarity. **d** Kernel density estimation plots illustrating the distribution of prediction errors against actual property values. The x-axis represents the actual measured values, the y-axis denotes the prediction errors (predicted minus actual), and the color intensity indicates the data density. Sample sizes for each property are indicated in parentheses within the subplot titles. The dense concentration of data points (darker blue regions) around the zero-error line demonstrates that the LG-Transformer model maintains high accuracy and stability across the common value ranges for each property. Source data are provided as a Source Data file. (EOV enthalpy of vaporization, ST surface tension, RON research octane number, MON motor octane number, DCN derived cetane number).

Advantages of the LG-Transformer in predicting key fuel properties. To comprehensively evaluate the performance advantages of LG-Transformer over other baseline models, statistical analysis and visualization are performed on the prediction errors of these models when predicting key properties of different fuel groups. Specifically, six representative properties are selected as representatives for further analysis, including three physical properties (EOV, viscosity, ST) and three chemical properties (RON, MON, and DCN), with evaluations conducted on representative folds for each property. These properties are selected because they collectively influence the behavior of a fuel during injection, evaporation, and combustion, which ultimately determine engine performance and emission levels. A visual analysis of the prediction performance for these properties is provided in Fig. 3. Specifically, Fig. 3a illustrates the training and testing loss curves for the MON prediction task, which demonstrates the excellent convergence achieved by the LG-Transformer. Figure 3b presents the five-fold cross-validation results of the LG-Transformer and baseline models on key fuel property prediction tasks. The box plots illustrate that LG-Transformer delivers superior predictive stability and accuracy for most properties, reflected by its tighter interquartile ranges and consistently higher median *R*^2^ values relative to other methods. The performance is particularly pronounced for critical metrics, such as RON and MON. These results suggest that LG-Transformer holds a distinct advantage in capturing the complex characteristics of fuel properties. Additionally, as shown in Fig. 3c and detailed in Supplementary Tab. 3, LG-Transformer demonstrates comprehensive superiority by achieving the highest *R*^2^ and the lowest or second-lowest MAE across all fuel groups. Its advantage is most pronounced in groups like HC and AROM, which represent major constituent families of practical engine fuels. Moreover, the model remains robust even under severe label sparsity. For example, it attains excellent accuracy in the small EPOX group (*n* = 6, *R*^2^ = 0.996) and Ac-X group (*n* = 5, *R*^2^ = 0.960). These results further demonstrate that LG-Transformer effectively captures structure-property relationships and maintains strong learning capability even when data availability is limited.

Furthermore, Fig. 3d provides a deeper performance analysis for the LG-Transformer using 2D Kernel Density Estimation (KDE) plots, which illustrate the relationship between prediction errors and true property values. For each property, the high-density regions (dark blue) are tightly concentrated around the zero-error line across the common range of actual values, confirming that LG-Transformer maintains high accuracy and stable performance throughout the typical value spectrum. Taking the prediction of RON and MON as an example, LG-Transformer predicts not only the narrowest error distribution but also shows exceptional accuracy in the high-octane region, making it a valuable tool for screening advanced gasoline blends. Similarly, for DCN, the LG-Transformer shows unparalleled consistency, maintaining high stability within the most common range (10 < *DCN* < 40) for diesel fuels, which underscores its reliability for practical applications.

### The effectiveness of learned-graph feature fusion

Ablation studies validate the effectiveness of learned-graph feature fusion. To elucidate the individual contributions of the key components within the LG-Transformer architecture, a systematic ablation study is conducted. The results, summarized in Table 3, quantify the predictive accuracy (*R*^2^ coefficient) of the full model against three degraded variants. One variant excludes the contrastive learning module (referred to as -Contrastive), another variant removes the GCN feature propagation module (referred to as -GCN), and the third variant removes both components (referred to as -LG), which is equivalent to a vanilla Transformer. Removing the contrastive learning module reduces the mean *R*^2^ to 0.879, indicating that the property-guided graph construction and learned embedding exploit the information well. A more significant performance drop occurs when the GCN feature propagation module is removed, with the mean *R*^2^ decreasing to 0.871. The most substantial degradation is observed in the -LG variant, where removing both modules causes the mean *R*^2^ to fall to 0.862. The ablation study quantitatively demonstrates that both the contrastive learning and GCN feature propagation modules contribute positively to the model performance, while GCN feature propagation is identified as the primary contributor. To further investigate model sensitivity to graph construction, the impact of two key hyperparameters is systematically evaluated. Specifically, the property similarity threshold is first varied (*τ* ∈ 2, 3, 4) to determine a stable value for generating high-quality embeddings, thereby ensuring the stable update of negative samples during the embedding training. Second, the neighbor weight coefficient (*α*_g_ ∈ 0.1, 0.5, 1.0) is adjusted to assess the contribution of neighbor nodes to feature fusion. The corresponding results are detailed in Supplementary Tabs. 2 and 3.

**Table 3:** Performance comparison of the ablation study models (*R*^2^ coefficients)

	Dataset	LG-Transformer	-Contrastive	-GCN	-LG
Physical Properties	Viscosity	**0.894** ± **0.0243**	0.873 ± 0.0301	0.857 ± 0.0187	0.854 ± 0.0373
	EOV	**0.979** ± **0.003**	0.972 ± 0.006	0.966 ± 0.004	0.962 ± 0.015
	SEF	**0.940** ± **0.044**	0.931 ± 0.047	0.928 ± 0.045	0.922 ± 0.055
	VP	**0.928** ± **0.070**	0.905 ± 0.075	0.872 ± 0.063	0.890 ± 0.039
	ST	**0.835** ± **0.133**	0.817 ± 0.140	0.815 ± 0.143	0.805 ± 0.097
	FP	**0.931** ± **0.020**	0.914 ± 0.024	0.897 ± 0.013	0.905 ± 0.031
	Density	**0.947** ± **0.017**	0.928 ± 0.013	0.932 ± 0.024	0.926 ± 0.020
	BP	**0.852** ± **0.030**	0.834 ± 0.034	0.816 ± 0.039	0.829 ± 0.017
	MP	**0.796** ± **0.044**	0.763 ± 0.049	0.771 ± 0.049	0.759 ± 0.017
Chemical Properties	RON	**0.856** ± **0.038**	0.825 ± 0.042	0.829 ± 0.045	0.815 ± 0.035
	MON	**0.807** ± **0.042**	0.772 ± 0.048	0.759 ± 0.051	0.741 ± 0.049
	CN	**0.843** ± **0.047**	0.822 ± 0.051	0.810 ± 0.052	0.792 ± 0.039
	DCN	**0.842** ± **0.066**	0.814 ± 0.072	0.794 ± 0.076	0.768 ± 0.066
	LHV	**0.995** ± **0.001**	0.993 ± 0.001	0.992 ± 0.003	0.993 ± 0.002
	UFL	**0.917** ± **0.013**	0.880 ± 0.018	0.883 ± 0.009	0.869 ± 0.025
	LFL	**0.953** ± **0.013**	0.927 ± 0.017	0.915 ± 0.020	0.869 ± 0.043
	YSI	**0.982** ± **0.005**	0.968 ± 0.010	0.966 ± 0.010	0.959 ± 0.019
MEAN		**0.900** ± **0.036**	0.879 ± 0.040	0.871 ± 0.039	0.862 ± 0.036

The table evaluates the impact of individual components in our LG-Transformer framework by systematically removing the contrastive learning module (-Contrastive), the GCN-based feature propagation module (-GCN), or both (-LG, equivalent to a vanilla Transformer). Results demonstrate that the complete LG-Transformer model consistently achieves the highest predictive accuracy across nearly all physicochemical properties. (*EOV* enthalpy of vaporization, *SEF* enthalpy of formation, *VP* vapor pressure, *ST* surface tension, *FP* flash point, *BP* boiling point, *MP* melting point, *RON* research octane number, *MON* motor octane number, *CN* cetane number, *DCN* derived cetane number, *LHV* lower heating value, *UFL* upper flammability limit, *LFL* lower flammability limit, *YSI* yield sooting index.).

^*^ The best-performing results are marked in bold.

**Fig. 4:**
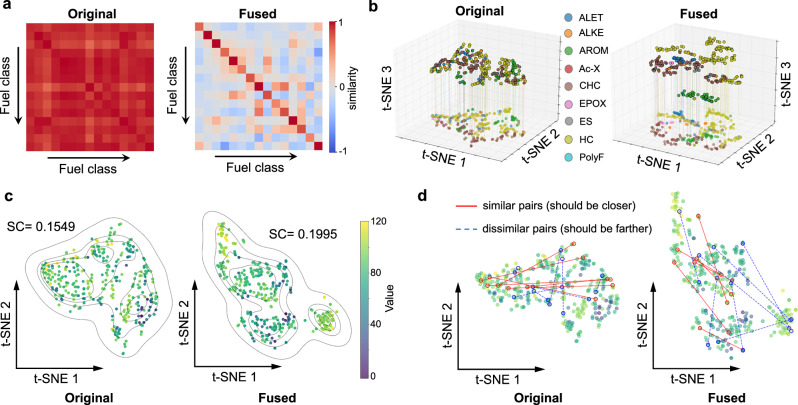
Validation of feature fusion on motor octane number (MON) dataset^24,37–41,43^ for improved molecular representation. **a** Cosine similarity comparison between original and fused feature representations across different fuel classes, demonstrating enhanced intra-class similarity after fusion and reduces inter-class similarity. **b** 3D t-distributed Stochastic Neighbor Embedding (t-SNE) visualization of molecular feature embeddings projected onto the t-SNE 1-t--SNE 2 plane. The 3D scatter points represent fuel molecules in the embedded feature space (t-SNE 1, 2, and 3), with translucent vertical lines connecting each point to its corresponding projection on the bottom plane (shown as open circles). **c** 2D t-SNE embedding of molecules colored continuously by their MON. Contour lines represent MON isoclines. The increased Spatial Continuity (SC) score for the fused features (right) compared to the original features (left) indicates a smoother and more accurate property landscape. **d** Visualization of the relative distance change for positive (red) and negative (blue) sample pairs in the t-SNE embedding space. Positive and negative pairs are defined by a MON similarity threshold. The fused features demonstrate the desired contrastive effect: positive pairs contract while negative pairs expand, shown on a unified scale with a normalized boundary margin. Source data are provided as a Source Data file. (HC Hydrocarbons, CHC Cyclic hydrocarbons, AROM Aromatics, ALET Alcohols & Ethers, ES Esters, ALKE Aldehydes & Ketones, Ac-X Acids & Derivatives, PolyF Polyfunctionals, EPOX Epoxides).

Visualization validates the effectiveness of learned-graph feature fusion. To rigorously validate the efficacy of the proposed learned-graph feature fusion mechanism, a systematic comparative analysis between the original and fused molecular descriptors is presented in Fig. 4, using MON as an example (Visualizations of other properties are shown in the Supplementary Figs. 1–3). Specifically, Fig. 4a displays heatmaps of the cosine similarity matrix between different fuel types. These plots confirm that the feature fusion process enhances intra-class correlations (intensified diagonal blocks) while suppressing inter-class noise (diminished off-diagonal intensities). Next, Fig. 4b provides a 3D t-SNE^92^ visualization of the molecular representations, where points are colored by their respective fuel group. This visualization further demonstrates the improved class separability, as the fused features form compact, well-separated clusters in stark contrast to the disordered original features. In Fig. 4c, the features are visualized in a 2D t-SNE plot, colored by their MON value and accompanied by contour lines of constant MON and the corresponding spatial continuity (SC) score (refer to Supplementary Eq. (3)). The analysis reveals that the fusion creates a smooth property manifold where feature geometry is highly correlated with the target property, an observation quantitatively confirmed by a 21% increase in the SC score, from 0.154 to 0.187. Finally, Fig. 4d visualizes the sample pairs defined during contrastive learning, with solid red lines connecting similar pairs and dashed lines connecting dissimilar pairs. This panel illustrates the mechanistic basis for the structural improvement, showing that the model successfully learns a property-aware metric by pulling similar molecules together while pushing dissimilar ones apart, providing a mechanistic basis for the enhanced predictive performance.

### Explaining the relationship between fuel molecular structure and properties using attention-based integrated gradients method

**Fig. 5:**
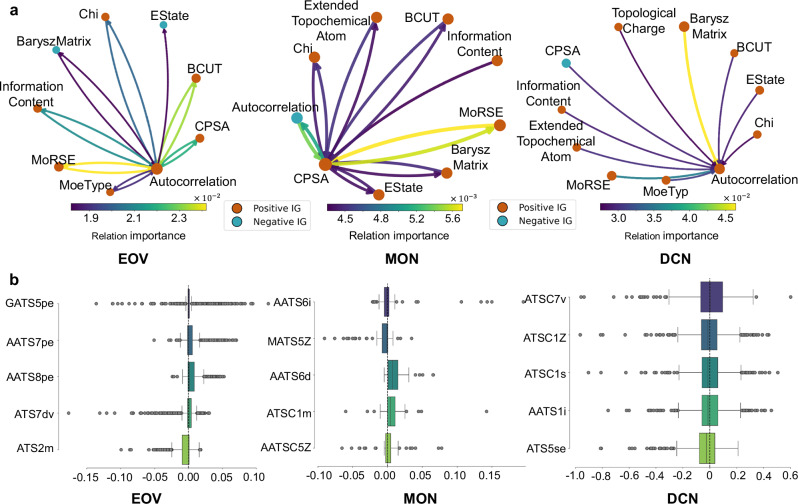
Visualization of model interpretability using a composite attention-integrated gradients (IG) framework. This figure illustrates the decision-making process of LG-Transformer for three fuel properties (EOV: enthalpy of vaporization^24,55^, MON: motor octane number^24,37 --41,43^, and DCN: derived cetane number^44,45,47 -- 53^) by assessing descriptor contributions. This method combines attention weights with IG contribution to provide a multi-layered and chemically intuitive explanation. **a** Descriptor Relation Networks visualize the interplay between different descriptor groups. In each network, a node represents a descriptor group. The size of the node is proportional to the attention weight assigned by the model and the IG contribution, indicating the importance of group. The node’s color corresponds to the cumulative IG contribution of that group to the prediction: orange/red tones signify a positive contribution (increasing the property value), while blue/teal tones signify a negative contribution, as indicated by the color bar. **b** Box plots provide a drill-down analysis of the top five individual contributing descriptors within the key groups `Autocorrelation'. Each plot shows the distribution of IG contribution for a single descriptor across all test set molecules, revealing the most influential underlying structure-property drivers. The detailed definitions of the descriptors are provided in Supplementary Tab. 1. Source data are provided as a Source Data file.

Traditional interpretations of attention-based models primarily rely on visualizing attention scores to assess feature importance. However, such approaches cannot often distinguish the directionality of influence (positive or negative) and do not provide a quantitative measure of contribution. To address these limitations, an enhanced interpretability framework is proposed by integrating attention mechanisms with the IG method. This combined approach is applied to the prediction of several key properties, including EOV, MON, and DCN, as shown in Fig. 5. The interpretability framework is visualized as a descriptor relation network (Fig. 5a), where each node corresponds to a descriptor group. The size of a node is proportional to the IG contribution value (refer to Supplementary Eq. (4)) of the group, reflecting its overall importance. The color of the node corresponds to the cumulative IG contribution. Orange tones indicate a positive contribution, and teal tones indicate a negative one. The thickness of the connections between nodes represents the magnitude of the attention score, while the direction indicates dependency. The color of a connection is jointly determined by both attention and IG scores. A brighter color means that the connection and its linked nodes have a greater overall influence on the model decisions. Furthermore, the box plots in Fig. 5b provide a deeper drill-down analysis. These plots show the distribution of IG scores for the top five contributing individual descriptors within the Autocorrelation descriptor group (refer to Supplementary Tab. 1). Heatmaps of attention scores and IG contribution plots for other properties are available in Supplementary Figs. 6 and 7.

This interpretability framework demonstrates that the model extracts chemically meaningful rules directly from molecular descriptor data. In predicting EOV, the model assigns the highest importance to features within the Autocorrelation descriptor group, especially those reflecting molecular polarizability and surface area. The drill-down plots highlight strong positive contributions from descriptors, such as GATS5pe and AATS7pe, consistent with the physical principle that larger and more polarizable molecules exhibit stronger dispersion forces and require more energy to vaporize^93^. Moreover, for MON prediction, the model learns to penalize features associated with molecular linearity and size, a finding evident in the significant negative contributions from descriptors, such as MATS5Z and AATSC5Z. This finding matches the well-known principle that straight-chain alkanes have lower anti-knock quality than branched or aromatic compounds. In contrast, for DCN, these same features show the opposite effect. Descriptors, such as ATSC1Z and ATSC1s emerge as strong positive contributors, aligning with the established understanding that straight-chain alkanes ignite more readily due to their structural favorability for low-temperature oxidation^50,94^. This contrasting behavior within the same descriptor family illustrates that the model can internalize nuanced and opposing chemical principles across different fuel performance metrics.

**Fig. 6:**
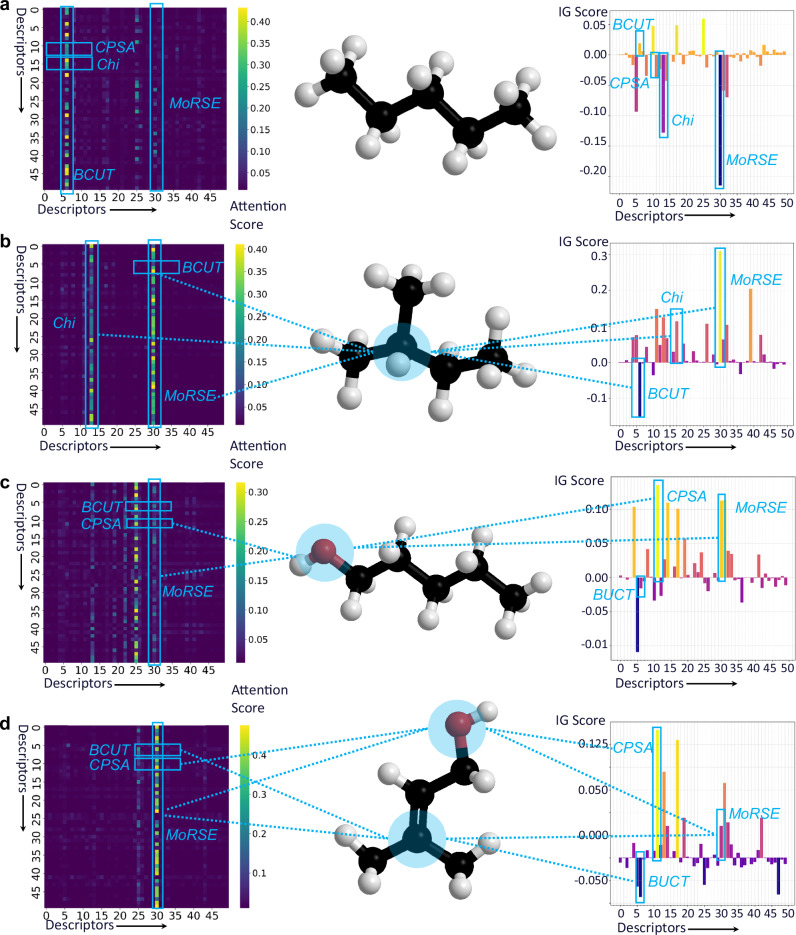
Interpretability analysis of LG-Transformer for MON prediction on representative fuel molecules. The figure illustrates the model’s decision-making process by integrating attention mechanism and Integrated Gradients analyses. Each subplot is divided into three panels: the attention heatmap (left), the molecular structure diagram (center), and the integrated gradients (IG) score bar chart (right). **a** For the baseline molecule n-pentane, the model identifies its linear architecture as negative contributors to MON. **b** For the branched alkane 2-methylbutane, the model attributes the MON increase to its compact, spherical topology. **c** For 1-pentanol, its strong polarity and hydrogen-bonding capacity are identified as the core positive contributor. **d** For Prenol, which incorporates both branching and a functional group, this model is capable of capturing their synergistic effects. The detailed definitions of the descriptors are provided in Supplementary Tab. 1. Source data are provided as a Source Data file.

Furthermore, to gain a deeper understanding of the decision-making mechanism of LG-Transformer in predicting specific fuel properties, an interpretability analysis is conducted using RON prediction as an example. Detailed analysis is presented in Fig. 6, which visualizes the molecular structures together with their attention patterns and IG scores. As shown in Fig. 6a, n-pentane (MON = 62.6) is used as the reference molecule with a zero vector baseline. Figure 6b–d then examine three structurally modified derivatives, namely 2-methylbutane (MON = 89.6), 1-pentanol (MON = 75.9), and prenol (MON = 74.2), capturing the effects of branching, the introduction of an oxygen-containing functional group, and the presence of multiple functional groups, respectively. This selection aims to investigate how the model captures the complex influence of different structural features on MON.

The results highlight that LG-Transformer captures MON-relevant structural variations primarily through key Mordred descriptors, including MoRSE for three-dimensional topology, Chi and BCUT for branching and electronic distribution, and CPSA for molecular polarity. Comparing Fig. 6b–d with Fig. 6a, MoRSE consistently acts as a positive contributor, as above structural modifications alter inter-molecular distances and increase topological complexity, resulting in higher MON relative to n-pentane. In Fig. 6b, the enhanced branching in 2-methylbutane further increases Chi and BCUT, reinforcing its high MON. For 1-pentanol (Fig. 6c), the introduction of the -OH group substantially increases polarity, reflected by a strong rise in CPSA, which drives MON above that of n-pentane. Comparing prenol with 1-pentanol (Fig. 6c, d), the introduction of the C=C bond modifies the electronic distribution and reduces BCUT, partially counteracting the polarity-driven increase and leading to a slight decrease in MON for prenol. These analyses demonstrate that LG-Transformer effectively captures inter-molecular topological features, directing attention to key structural elements and quantitatively linking them to variations in fuel properties.

## Discussion

To address the challenge of accurately predicting different physicochemical properties across a wide range of fuels, this study first constructs a large-scale and diverse fuel property database. Building on this foundation, a hierarchical deep learning framework, namely LG-Transformer, is developed. By incorporating a learned-graph feature fusion strategy guided by contrastive learning, the model achieves state-of-the-art performance, substantially surpassing both traditional machine learning, conventional deep learning and existing GNN-based and contrastive learning baselines. Moreover, the framework offers chemically meaningful interpretations of its predictions, demonstrating its ability to capture the underlying structure-property relationships that govern fuel behavior. Building on these results, the following discussion evaluates the robustness and applicability of LG-Transformer under two extended scenarios: (i) predicting properties of unseen fuel molecules and (ii) generalizing the model across different molecule property prediction tasks.

To this end, an independent external dataset is constructed from two additional sources^24,68^, comprising 88 RON, 37 MON, 48 EOV, 172 BP, and 69 density data points, including 260 previously unseen fuel molecules. Learned graphs are then constructed for these unseen fuels (methodology detailed in Sec. 4.6), and LG-Transformer is applied to predict their properties. The results, summarized in Table 4a, are compared with multiple baseline methods, showing that LG-Transformer consistently achieves the highest *R*^2^ across all properties. These outcomes demonstrate the robustness of the model and reliable predictive performance on previously untrained molecular data.

**Table 4:** Generalization performance of LG-Transformer on external fuel properties and public molecular benchmarks

(a) *R*^2^ scores on external test set					
Dataset	LG-Transformer	Catboost^24^	Transformer^29^	MolCLR^89^	AttentiveFP^84^
RON	**0.886** ± **0.038**	0.835 ± 0.049	0.825 ± 0.032	0.857 ± 0.042	0.866 ± 0.026
MON	**0.830** ± **0.067**	0.763 ± 0.079	0.787 ± 0.058	0.817 ± 0.064	0.789 ± 0.069
EOV	**0.956** ± **0.013**	0.949 ± 0.029	0.940 ± 0.038	0.949 ± 0.018	0.944 ± 0.025
BP	**0.831** ± **0.049**	0.770 ± 0.048	0.808 ± 0.038	0.789 ± 0.049	0.824 ± 0.058
Density	**0.954** ± **0.006**	0.914 ± 0.016	0.887 ± 0.009	0.934 ± 0.013	0.940 ± 0.006
MEAN	**0.892** ± **0.035**	0.846 ± 0.044	0.849 ± 0.035	0.869 ± 0.037	0.873 ± 0.037

(b) MoleculeNet benchmarks					
Model	Physical chemistry (RMSE)			Quantum mechanics (MAE)	
	ESOL	FreeSolv	Lipophilicity	QM7	QM8
GCN^102^	1.431 ± 0.050	2.870 ± 0.135	0.712 ± 0.049	122.9 ± 2.2	0.0366 ± 0.0000
GIN^103^	1.452 ± 0.020	2.765 ± 0.180	0.850 ± 0.071	124.8 ± 0.7	0.0371 ± 0.001
MPNN^104^	1.167 ± 0.430	1.621 ± 0.952	0.672 ± 0.051	111.4 ± 0.9	0.0148 ± 0.001
DMPNN^105^	1.050 ± 0.008	1.673 ± 0.082	0.683 ± 0.016	103.5 ± 8.6	0.0156 ± 0.001
CMPNN^106^	0.798 ± 0.112	1.570 ± 0.442	0.614 ± 0.029	75.1 ± 3.1	0.0153 ± 0.002
N -GRAM^107^	1.100 ± 0.030	2.510 ± 0.191	0.880 ± 0.121	125.6 ± 1.5	0.0320 ± 0.003
Hu et al.^108^	1.100 ± 0.006	2.764 ± 0.002	0.739 ± 0.003	113.2 ± 0.6	0.0215 ± 0.001
GEM^109^	0.813 ± 0.028	1.748 ± 0.114	0.674 ± 0.022	60.0 ± 2.7	0.0163 ± 0.001
GROVER^110^	1.423 ± 0.288	2.947 ± 0.615	0.823 ± 0.010	91.3 ± 1.9	0.0182 ± 0.001
MolCLR^89^	1.113 ± 0.023	2.301 ± 0.247	0.789 ± 0.009	90.9 ± 1.7	0.0185 ± 0.013
MolCLR_CMPNN_^89^	0.911 ± 0.082	2.021 ± 0.133	0.875 ± 0.003	89.8 ± 6.3	0.0179 ± 0.001
KANO^90^	0.797 ± 0.098	1.670 ± 0.076	0.68 ± 0.035	75.2 ± 2.8	0.0147 ± 0.0012
LG-Transformer	**0.5859** ± **0.022**	**0.9531** ± **0.195**	**0.587** ± **0.024**	**53.2** ± **3.4**	**0.010255** ± **0.0004**

^a^*R*^2^ scores ( ± std) on external fuel property test datasets (*EOV* enthalpy of vaporization, *BP* boiling point, *RON* research octane number, *MON* motor octane number). ^b^Performance on MoleculeNet benchmarks, with RMSE reported for physical chemistry tasks (ESOL, FreeSolv, Lipophilicity) and MAE for quantum mechanics tasks (QM7, QM8). The best results in each panel are highlighted in bold.

^*^ The best-performing results in each column are marked in bold.

On the other hand, to evaluate the generalization capability of LG-Transformer beyond fuel property prediction, an extensive experiment is conducted on the publicly available MoleculeNet benchmark^95^, a complex dataset widely used for molecular machine learning evaluation. Five related regression tasks are selected, including ESOL, FreeSolv, and Lipophilicity from physical chemistry, and QM7 and QM8 from quantum mechanics. LG-Transformer is then applied to these tasks and evaluated against established graph-based and contrastive learning methods, with the results summarized in Table 4b. It is readily observed that LG-Transformer achieves state-of-the-art performance across all selected tasks, with particularly strong results on QM7, a high-dimensional and challenging dataset, demonstrating its ability to capture complex electronic and structural relationships. These findings confirm that the architecture is effective not only for fuel-specific property prediction but also generalizes broadly to diverse molecular regression problems.

To summarize, a central contribution of this work lies in its two-stage approach to representation learning. Unlike conventional methods that feed raw or engineered descriptors directly into a predictive model, the LG-Transformer first constructs a property-centric molecular similarity graph. The contrastive learning module learns to map molecules into a latent space where distance correlates with property similarity, not just structural similarity. The subsequent GCN feature propagation then enriches the feature vector of each molecule with information from its property-based neighbors. This process effectively resolves the issue of feature disorder, providing the attention mechanism of the Transformer with structured, context-aware inputs that explicitly encode the complex, non-linear relationships between descriptors and target properties. The success of the LG-Transformer hybrid architecture offers broader implications for the data-driven design of diverse chemical and engineering domains, primarily by demonstrating the powerful synergy between representation learning with an induced relational bias and attention-based Transformer models. This paradigm offers a potent alternative to approaches that rely on massive, unannotated datasets (e.g., SMILES pre-training), by instead learning rich representations from smaller and well-annotated datasets.

In conclusion, this work presents a comprehensive, multi-class fuel property database accessible at https://ai4fuel.sjtu.edu.cn/, offering a valuable foundation for the engine community. Furthermore, a robust and interpretable deep learning framework is developed, achieving state-of-the-art accuracy in predicting fuel properties and effectively linking molecular structures to complex performance metrics. These integrated approaches establish a solid platform to accelerate AI-driven optimization of next-generation green fuels.

## Methods

### Problem definition

Given a set of samples *T* = {*m*_*i*_∣*i* = 1, 2, …, *n*} containing *n* fuel molecules, each *m*_*i*_ is represented by a *k*_*m*_ dimension descriptor vector **x**_*i*_. And the corresponding measured fuel property *y*_*i*_ for the molecule *m*_*i*_ is also provided. The core objective is to construct a data-driven model that captures the implicit relationships between molecular descriptors and physicochemical properties. For any molecule *m*_*i*_, the model aims to minimize the prediction error between the estimated value of the model $\widehat{{y}_{i}}$ and the experimentally measured value *y*_*i*_.

To solve this problem, a feature fusion method based on contrastive learning is proposed, which is combined with a sequence model to predict the molecular property values. Firstly, contrastive learning is employed to improve the representation of molecular descriptors. Furthermore, the attention mechanism of the Transformer is leveraged to generate embeddings to capture the intricate correlations within fuel molecules. Finally, an MLP network is used to predict fuel property values based on the generated embeddings.

### Embedding network based on contrastive learning

The molecular descriptors calculated by Mordred^85^ primarily characterize chemical structures, but cannot fully capture the interrelationships between molecular properties. To encapsulate both molecular structural information and feature-property correlations, contrastive learning is adopted to train an MLP network. Given the molecular descriptor ${{{{\bf{x}}}}}_{i}\in {{\mathbb{R}}}^{{k}_{m}}$, this network maps the input **x**_*i*_ to an embedding ${{{{\bf{e}}}}}_{i}\in {{\mathbb{R}}}^{{k}_{e}}$ using a weight matrix ${{{{\bf{W}}}}}_{e}\in {{\mathbb{R}}}^{{k}_{m}\times {k}_{e}}$ and a bias vector ${{{{\bf{b}}}}}_{e}\in {{\mathbb{R}}}^{{k}_{e}}$ as follows:

1 $${{{{\bf{e}}}}}_{i}=ReLU({{{{\bf{W}}}}}_{e}{{{{\bf{x}}}}}_{i}+{{{{\bf{b}}}}}_{e}),$$

where *Relu*( ⋅ ) denotes a non-linear activation function.

To ensure that samples with similar labels have similar representations in the embedding space, the positive sample mask matrix ${{{{\bf{M}}}}}_{pos}\in {{\mathbb{R}}}^{n\times n}$ is constructed. The off-diagonal element **M**_*pos*_(*i*, *j*) (where *i*, *j* ∈ {1, …, *n*} and *i* ≠ *j*) is determined based on the absolute distance between the experimental property values *y*_*i*_ and *y*_*j*_. The specific definition is as follows:

2 $${{{{\bf{M}}}}}_{pos}(i,j)={\mathbb{I}}[| {y}_{i}-{y}_{j}| \le \delta ],$$

where ${\mathbb{I}}$ is the indicator function that equals one if the condition is satisfied, otherwise it will be zero. In addition, the diagonal element **M**_*pos*_(*i*, *i*) is set to 0. With this formula, a pair of different samples is defined as a positive pair if their normalized absolute label distance is less than *δ*.

Furthermore, a sample pair with dissimilar property values and similar feature vectors is defined as a negative pair. For each pair of molecules *m*_*i*_ and *m*_*j*_, *d*_*ij*_ is employed to calculate the Euclidean distance between their corresponding embeddings **e**_*i*_ and **e**_*j*_. By introducing the feature distance threshold *τ*, the negative sample mask matrix ${{{{\bf{M}}}}}_{{{{\rm{neg}}}}}\in {{\mathbb{R}}}^{n\times n}$ is constructed as follows:

3 $${{{{\bf{M}}}}}_{neg}(i,j)={\mathbb{I}}\left\{{{{{\bf{M}}}}}_{pos}(i,j)=0\wedge {d}_{ij} < \tau \right\}.$$

To correctly capture the implicit connections between molecular features and properties, the loss function is formulated as follows:

4 $${{{\mathcal{L}}}}=\frac{1}{{N}_{pos}}{\sum }_{i,j}{{{{\bf{M}}}}}_{pos}(i,j){d}_{ij}^{2}+\frac{1}{{N}_{neg}}{\sum }_{i,j}{{{{\bf{M}}}}}_{neg}(i,j)max{(\tau -{d}_{ij},0)}^{2},$$

where *N*_*pos*_ and *N*_*neg*_ respectively represent the number of positive sample pairs and negative sample pairs. This formulation guarantees that positive sample pairs are closer in Euclidean distance in the embedding space, while negative sample pairs are separated by at least a distance of *τ*. This method can be leveraged to obtain a topologically ordered embedding in the target property space. These embeddings will be used subsequently for feature propagation operations based on GCN.

### GCN Feature propagation based on embedding

Once the embedding is generated, corresponding similarities can be constructed for these embedding vectors. Given a pair of molecules *m*_*i*_ and *m*_*j*_, the similarity matrix ${{{\bf{S}}}}\in {{\mathbb{R}}}^{n\times n}$ for their corresponding embeddings **e**_*i*_ and **e**_*j*_ is constructed as follows:

5 $${{{\bf{S}}}}(i,j)={{{{\bf{e}}}}}_{i}\cdot {{{{\bf{e}}}}}_{j}^{T}.$$

With this definition, the similarity matrix **S** is dense, which will increase the computational load of feature fusion. In order to sparsify this matrix, the *k*-nearest neighbors (KNN) algorithm is adopted to construct the adjacency graph. Then, the sparse adjacency matrix ${{{\bf{G}}}}\in {{\mathbb{R}}}^{n\times n}$ is constructed as follows:

6 $${{{\bf{G}}}}(i,j)=\left\{\begin{array}{ll}{{{\bf{S}}}}(i,j),& \,{{{\rm{if}}}}\,j\in {{{{\mathcal{N}}}}}_{k}({m}_{i})\\ 0,& \,{{{\rm{otherwise}}}}\,.\end{array}\right.$$

where ${{{{\mathcal{N}}}}}_{k}({m}_{i})\subseteq \{1,2,\ldots,n\}$ denotes the k-nearest neighbor set of the fuel molecule *m*_*i*_. The sparse adjacency matrix **G** represents the predicted similarity between each molecule and its neighbors.

To balance the influence of neighbors on the molecule, the adjustable parameter *α*_g_ is introduced to regulate the weight of neighbors. In addition, a self-loop is added to preserve the characteristics of the node itself, so that the adjacency matrix ${{{\bf{A}}}}\in {{\mathbb{R}}}^{n\times n}$ is constructed as follows:

7 $${{{\bf{A}}}}={{{\bf{I}}}}+{\alpha }_{g}\cdot Norm({{{\bf{G}}}}),$$

where *Norm*(**⋅**) denotes a row-wise normalization function applied to the matrix and **I** represents the identity matrix. Then, the construction of the symmetric normalized adjacency matrix $\widetilde{{{{\bf{A}}}}}\in {{\mathbb{R}}}^{n\times n}$ is as follows:

8 $$\widetilde{{{{\bf{A}}}}}={\widetilde{{{{\bf{D}}}}}}^{-\frac{1}{2}}{{{\bf{A}}}}{\widetilde{{{{\bf{D}}}}}}^{-\frac{1}{2}},$$

where $\widetilde{{{{\bf{D}}}}}$ is the diagonal degree matrix with entries $\widetilde{{{{\bf{D}}}}}(i,i)={\sum }_{j}{{{\bf{A}}}}(i,j)$. The fused feature vector ${\widetilde{{{{\bf{x}}}}}}_{i}\in {{\mathbb{R}}}^{{k}_{m}}$ corresponding to the descriptor feature **x**_*i*_ can be obtained through GCN feature propagation, which is defined as follows:

9 $${\widetilde{{{{\bf{x}}}}}}_{i}={\widetilde{a}}_{ii}{{{{\bf{x}}}}}_{i}+{\sum }_{j\in {{{{\mathcal{N}}}}}_{k}({m}_{i})}{\widetilde{a}}_{ij}{{{{\bf{x}}}}}_{j}$$

where **x**_*j*_ is the original feature vector of the molecule *m*_*j*_, ${\widetilde{a}}_{ij}$ is the element in the *i*-th row and *j*-th column of $\widetilde{{{{\bf{A}}}}}$. This enables the propagation of features based on the neighbor relationships of molecules, thereby achieving the effect of feature enhancement. Note that, in order to avoid excessive introduction of noise and improve computational efficiency, only a single-layer feature propagation method is employed for feature fusion as^96^.

### Encoder-based transformer architecture

The regression prediction model used in this study adopted a transformer encoder architecture, which can automatically capture the implicit relationships within molecules. To better utilize the chemical characteristics contained in molecular descriptors, each fused descriptor ${\widetilde{{{{\bf{x}}}}}}_{i}\in {{\mathbb{R}}}^{{k}_{m}}$ is classified according to the descriptor group defined by Mordred^85^, thereby obtaining the input ${\widehat{{{{\bf{x}}}}}}_{i}\in {{\mathbb{R}}}^{{k}_{s}\times {k}_{d}}$, where *k*_*s*_ denotes the number of descriptor categories and *k*_*d*_ denotes the feature dimension of each descriptor category. For groups containing fewer than *k*_*d*_ descriptors, the remaining positions are padded with zeros.

The encoder architecture is mainly divided into a multihead self-attention module and a feed-forward network (FFN) module. For the molecule *m*_*i*_, the input at layer *l* is denoted as ${\widehat{{{{\bf{x}}}}}}_{i}^{(l)}$. All inputs are first calculated by the multihead self-attention module as follows:

10 $${{{{\bf{Q}}}}}_{h}^{(l)}={\widehat{{{{\bf{x}}}}}}_{i}^{(l)}{{{{\bf{W}}}}}_{Q}^{(l)},\,{{{{\bf{K}}}}}_{h}^{(l)}={\widehat{{{{\bf{x}}}}}}_{i}^{(l)}{{{{\bf{W}}}}}_{K}^{(l)},\,{{{{\bf{V}}}}}_{h}^{(l)}={\widehat{{{{\bf{x}}}}}}_{i}^{(l)}{{{{\bf{W}}}}}_{V}^{(l)},$$

11 $${{{{\mathcal{A}}}}}_{h}^{(l)}=softmax\left(\frac{{{{{\bf{Q}}}}}_{h}^{(l)}{\left({{{{\bf{K}}}}}_{h}^{(l)}\right)}^{\top }}{\sqrt{{k}_{q}}}\right){{{{\bf{V}}}}}_{h}^{(l)},$$

12 $$\,{{{\rm{MultiHead}}}}\,({{{{\bf{Q}}}}}^{(l)},{{{{\bf{K}}}}}^{(l)},{{{{\bf{V}}}}}^{(l)})=\,{{{\rm{Concat}}}}\,({{{{\mathcal{A}}}}}_{1}^{(l)},\ldots,{{{{\mathcal{A}}}}}_{{N}_{H}}^{(l)}){{{{\bf{W}}}}}^{(l)},$$

where *h* = 1, …, *N*_*H*_, *k*_*q*_ = *k*_*d*_/*N*_*H*_. ${{{{\bf{W}}}}}_{Q}^{(l)}\in {{\mathbb{R}}}^{{k}_{d}\times {k}_{q}},{{{{\bf{W}}}}}_{K}^{(l)}\in {{\mathbb{R}}}^{{k}_{d}\times {k}_{q}},{{{{\bf{W}}}}}_{V}^{(l)}\in {{\mathbb{R}}}^{{k}_{d}\times {k}_{q}}$ and ${{{{\bf{W}}}}}^{(l)}\in {{\mathbb{R}}}^{{k}_{d}\times {k}_{d}}$ are trainable weight matrices. Then the molecular descriptors are calculated by the feed-forward network as follows:

13 $${\widehat{{{{\bf{x}}}}}}_{i}^{{(l)}^{{\prime} }}=\,{{{\rm{LayerNorm}}}}\,\left({\widehat{{{{\bf{x}}}}}}_{i}^{(l)}+\,{{{\rm{MultiHead}}}}\,({{{{\bf{Q}}}}}^{(l)},{{{{\bf{K}}}}}^{(l)},{{{{\bf{V}}}}}^{(l)})\right),$$

14 $${\widehat{{{{\bf{x}}}}}}_{i}^{(l+1)}=\,{{{\rm{LayerNorm}}}}\,\left({\widehat{{{{\bf{x}}}}}}_{i}^{{(l)}^{{\prime} }}+ReLU\left({\widehat{{{{\bf{x}}}}}}_{i}^{{(l)}^{{\prime} }}{{{{\bf{W}}}}}_{1}+{{{{\bf{b}}}}}_{1}\right){{{{\bf{W}}}}}_{2}+{{{{\bf{b}}}}}_{2}\right),$$

where **W**_1_, **W**_2_ are trainable weight matrices and **b**_1_, **b**_2_ are bias vectors. ${\widehat{{{{\bf{x}}}}}}_{i}^{({l}_{n})}$ is used to represent the output of the entire encoder architecture, where *l*_*n*_ represents the number of layers. Finally, a task-specific MLP network projects the refined descriptors onto the estimated attribute values ${\widehat{y}}_{i}$:

15 $${\widehat{y}}_{i}=ReLU\left({{{{\bf{W}}}}}_{t}{\widehat{{{{\bf{x}}}}}}_{i}^{({l}_{n})}+{{{{\bf{b}}}}}_{t}\right),$$

where **W**_*t*_ denotes a weight matrix and **b**_*t*_ denotes a bias vector.

Based on the distribution of the property values in the dataset, the MSE loss function is selected for training, defined as follows:

16 $${{{{\mathcal{L}}}}}_{MSE}=\frac{1}{n}{\sum }_{i=1}^{n}{\left({\widehat{y}}_{i}-{y}_{i}\right)}^{2},$$

where *y*_*i*_ and ${\widehat{y}}_{i}$ are the ground truth and predicted value, respectively.

### Data preparation

The database is constructed by integrating fuel property data from multiple literature sources and existing databases. Duplicate molecular entries are first removed based on isomeric SMILES. After deduplication, measurement uncertainties for the retained properties are evaluated to assess data reliability, and the corresponding values are summarized in Table 1. Note that, when available, uncertainties are taken directly from the source literature. Otherwise, they are adopted from widely recognized standards or derived from expertise based on the measurement method. This process ensures consistent quality control throughout the dataset^46^. According to the uncertainty analysis, when multiple sources report property values for the same molecule, priority is given to experimental measurements obtained under widely recognized standards, such as ASTM. Subsequently, for each fuel molecule in the dataset, the corresponding SMILES string is retrieved from PubChem or the literature. Any missing entries are generated using the OPSIN toolkit^97^. Molecular descriptors are then computed based on these SMILES strings using Mordred^85^.

### Model training

The model training process consists of two primary stages. In the first stage, a contrastive learning framework is used to pre-train the embedding network of molecular descriptors, which contains two hidden layers. To mitigate scale discrepancies between features and to stabilize the training process, we applied standardization to both features and labels. The network is trained using the Adam optimizer, and the Spearman correlation coefficient is used as the primary metric to evaluate the monotonic relationship between learned representations and fuel properties. The detailed training setting of this embedding network is presented in Supplementary Tab. 7. Notably, updating all negative sample pairs in each iteration can impede model convergence. To address this, a random sampling strategy is implemented, selecting only five random negative pair for updates in each training step.

The second stage is to train a transformer-based regression model with the learned features from the first stage. To achieve a more uniform label distribution, logarithmic transformation and normalization are applied to the target labels. The Adam optimizer is employed for model training. The hyperparameters of the model are optimized using an Optuna-based Bayesian search^91^, where the model is trained on the training set and evaluated on the validation set to identify the optimal configuration. After obtaining the best hyperparameters, the Transformer model is retrained on both the training and validation sets using these optimal settings to produce the final model for inference. The detailed search spaces of hyperparameters and the experimental settings are provided in Supplementary Tables. 6 and 7. Furthermore, Supplementary Fig. 8 provides a comparison of the training efficiency between the LG-Transformer and other baselines.

### Model Inference

After the model is well trained, the model inference for the new molecule *m*_*p*_ can be conducted with its original descriptor **x**_*p*_. Firstly, the learned embedding **e**_*p*_ of molecule *m*_*p*_ could be obtained as Eq. (1). Subsequently, the similarity **s**_*p*_ between *m*_*p*_ and all known molecules is computed as

17 $${{{{\bf{s}}}}}_{p}={{{{\bf{e}}}}}_{p}\cdot {{{{\bf{E}}}}}_{A}^{\top },$$

where **E**_*A*_ is the original descriptor matrix of all known molecules. The sparse connection **g**_*p*_ of *m*_*p*_ is then obtained according to Eq. (6) as

18 $${{{{\bf{g}}}}}_{p}(i)=\left\{\begin{array}{ll}{{{{\bf{s}}}}}_{p}(i),& \,{{{\rm{if}}}}\,i\in {{{{\mathcal{N}}}}}_{k}({m}_{p})\\ 0,& \,{{{\rm{otherwise}}}}\,,\end{array}\right.$$

where **s**_*p*_(*i*) is the *i*-th element of vector **s**_*p*_, and ${{{{\mathcal{N}}}}}_{k}({m}_{p})$ denotes the k-nearest neighbor set in known molecules of the fuel molecule *m*_*p*_. After obtaining the connection vector **g**_*p*_ for molecule *m*_*p*_, the corresponding adjacency vector is computed as ${{{{\bf{a}}}}}_{p}={\alpha }_{g}\frac{{{{{\bf{g}}}}}_{p}}{\parallel {{{{\bf{g}}}}}_{p}{\parallel }_{1}}$. The degree of the new molecule is *d*_*p*_ = 1 + ∑_*j*_**a**_*p*_(*j*). For each known molecule *m*_*j*_, let *d*_*j*_ be its corresponding degree as *d*_*p*_. Finally, the fused feature ${\widetilde{{{{\bf{x}}}}}}_{p}$ of *m*_*p*_ can be written in the same form as Eq. (9):

19 $${\widetilde{{{{\bf{x}}}}}}_{p}={\widetilde{a}}_{p,p}{{{{\bf{x}}}}}_{p}+{\sum }_{j\in {{{{\mathcal{N}}}}}_{k}({m}_{p})}{\widetilde{a}}_{p,j}{{{{\bf{x}}}}}_{j},$$

with coefficients ${\widetilde{a}}_{p,p}=1/{d}_{p}$ and ${\widetilde{a}}_{p,j}={{{{\bf{a}}}}}_{p}(j)/(\sqrt{{d}_{p}}\sqrt{{d}_{j}})$.

The fused feature ${\widetilde{{{{\bf{x}}}}}}_{p}$ obtained above is then divided into descriptor groups according to the predefined mapping, and each group is zero-padded to a fixed dimensional width to form a consistent token representation. The resulting grouped and padded feature sequence is finally fed into the pretrained Transformer regression model to generate the property prediction for the new molecule.

### Evaluation metrics

The squared Pearson correlation coefficient (*R*^2^) and the mean absolute error (MAE) are employed to evaluate the efficacy of the model predictions. These are defined as follows:

20 $${R}^{2}=1-\frac{{\sum }_{i=1}^{n}{( \, {y}_{i}-{\widehat{y}}_{i})}^{2}}{{\sum }_{i=1}^{n}{( \, {y}_{i}-{\overline{y}})}^{2}},$$

21 $${{{\mathrm{MAE}}}}\,=\frac{1}{n}{\sum }_{i=1}^{n}\left|{y}_{i}-{\widehat{y}}_{i}\right|$$

where *n* denotes the sample size, *y*_*i*_ denotes the measured property value, ${\widehat{y}}_{i}$ denotes the predicted value, and ${\overline{y}}=\frac{1}{n}{\sum }_{i=1}^{n}{y}_{i}$ denotes the mean observed value.

### Baselines

In order to evaluate the performance of the method proposed in this paper for predicting fuel properties, a diverse set of relevant baselines spanning multiple modeling paradigms is selected. These include traditional machine learning models, such as SVR^23^, MLP^23^, CatBoost^24^, and ML-QSPR^86^. Conventional deep learning architectures operating on non-graph molecular representations are also incorporated, specifically a SMILES-based CNN^87^ and a vanilla Transformer^29^. In addition, it also includes some representative graph-based methods for predicting molecular properties^83,84,88^, such as Chemprop^88^ and AttentiveFP^84^. To ensure a fair comparison with state-of-the-art approaches in molecular representation learning, contrastive learning models MolCLR^89^ and KANO^90^ are further added. The same dataset partitioning is applied across all models, and hyperparameter optimization is performed using Optuna^91^ within the search space specified in Supplementary Tables. 5 and 7.

## Supplementary information

Supplementary Information

Transparent Peer Review file

## Source data

Source data for Figure 5

Source data for Figure 6

Source data for Supplementary Figures 1-3

Source data for Supplementary Figure 6

Source data for Supplementary Figure 7

Source data for Supplementary Figure 8

## Data Availability

Source data are provided with this paper. All fuel databases proposed in this work are open source on figshare: 10.6084/m9.figshare.29571449^98^. Source data are provided with this paper.

## Code availability

All the codes and the version used in this publication are freely available in the Github repository: https://github.com/furyIndex/AI4fueland the Zenodo: 10.5281/zenodo.19050612^99^. The source code of this work is also freely available in the Code Ocean capsule at 10.24433/CO.0191841.v1^100^.
