# Supplementary material for: LG-Transformer: learned-graph transformer framework enabling diverse physicochemical properties prediction toward fuel design
Source: Nat Commun. 2026 Jun 3;17:7137. doi: 10.1038/s41467-026-73853-z (PMC13396237; doi:10.1038/s41467-026-73853-z)
Supplement: Supplementary file 1 — Supplementary Information [file 41467_2026_73853_MOESM1_ESM.pdf]

Supplementary Information

LG-Transformer: Learned-Graph Transformer Framework Enabling Diverse Physicochemical Properties Prediction toward Fuel Design

Jiabo Zhang[a], Xiang Lv[b], Hui An[a], Jing Li[c], Peng Han[d,*], Zhen Huang[a,*]

[a]*Key Laboratory for Power Machinery and Engineering, Shanghai Jiao Tong University, Shanghai 200240, China*

[b]*Department of Computer Science, University of Electronic Science and Technology of China, Chengdu, 610054, China*

[c]*Harbin Institute of Technology (Shenzhen), Shenzhen, 518055, China*

[d]*School of Software Engineering, Faculty of Electronic and Information Engineering, Xi'an Jiaotong University, Xi'an 710049, China*

* ***Correspondence:***

Peng Han: penghan_study@foxmail.com

Zhen Huang: z-huang@sjtu.edu.cn

1. Supplementary Tables

Supplementary Tab. 1 shows all the descriptor groups defined by the Mordred descriptor calculation tool [1] used in this paper and their corresponding meanings.

Supplementary Table 1. Description of the 50 descriptor groups.

Descriptor Group	Explanation
ABCIndex	A topological index that quantifies the connectivity between atoms and bonds in a molecule.
AcidBase	An indicator of molecular acidity or basicity.
AdjacencyMatrix	Descriptors calculated from the molecular adjacency matrix, reflecting direct atomic connections.
Aromatic	A descriptor that counts the number of aromatic atoms and bonds within a molecule.
AtomCount	A descriptor that counts the total number of various types of atoms in a molecule.
Autocorrelation	An autocorrelation descriptor that encodes the spatial distribution patterns of topological structure and physicochemical properties.
BCUT	Circular fingerprints generated from the molecular topology to describe structural features.
BalabanJ	A topological index that combines information on average distance and connectivity.
BaryszMatrix	Descriptors calculated from the Barysz matrix, which reflects vertex and edge weights.
BertzCT	An index that measures molecular complexity based on structural connectivity.
BondCount	A descriptor that counts the number of different types of chemical bonds in a molecule.
CPSA	An index that describes charge distribution by combining molecular surface area with partial charge information.
CarbonTypes	An index describing the types of carbon atoms and their hybridization ratios in a molecule.
Chi	A topological index that characterizes molecular complexity and connectivity.
Constitutional	An index describing the basic composition of a molecule, such as the number of atoms, bonds, and rings.
DetourMatrix	A graph theory descriptor based on the longest path lengths between atoms in a molecule (the detour matrix).
DistanceMatrix	A graph theory descriptor based on the shortest distances between atoms in a molecule (the distance matrix).
EState	An electro-topological state descriptor that identifies key topological features and fragments affecting molecular activity.
EccentricConnectivityIndex	A topological index combining atomic connectivity and eccentricity information.
ExtendedTopochemicalAtom	An ETA descriptor that quantifies core atomic properties such as valence electron count and period number.
FragmentComplexity	A descriptor used to quantify the structural complexity of molecular fragments.
Framework	A ratio descriptor describing the structural features of a molecule's scaffold and framework.
GeometricalIndex	An index describing the 3D geometry of a molecule, such as its diameter, radius, and shape index.
GravitationalIndex	An index based on an analogy to universal gravitation, calculated from the mass distribution of atoms within the molecule.
HydrogenBond	A descriptor that counts the number of hydrogen bond donors and acceptors in a molecule.
InformationContent	An index based on information theory that measures the complexity and information content of a molecular structure.
KappaShapeIndex	An index that quantifies molecular shape features such as size, cyclicity, and degree of branching.
Lipinski	A filter for evaluating drug-likeness based on rules like Lipinski's Rule of Five.
LogS	A descriptor that estimates the aqueous solubility (LogS) of a molecule by analyzing its structure.
McGowanVolume	A descriptor used to quantify and estimate the volumetric properties of a molecule.

Continued on next page

Table 1 – continued from previous page

Descriptor Group	Explanation
MoRSE	A representation of molecular structure calculated from 3D atomic coordinates, based on concepts from electron diffraction.
MoeType	A series of descriptors used to estimate molecular surface area, such as the solvent-accessible surface area.
MolecularDistanceEdge	A descriptor that calculates the distance edges between specific pairs of atom types in a molecule.
MolecularId	A unique or semi-unique identifier generated from molecular structural information.
MomentOfInertia	The moment of inertia, describing the mass distribution and 3D shape of a molecule.
PBF	A descriptor that quantifies 3D structural properties by measuring atomic distances to a best-fit plane.
PathCount	A descriptor that counts the number of paths of a specific length within the molecular graph.
Polarizability	An index describing a molecule's ability for its charge distribution to shift under an external electric field.
RingCount	A descriptor that counts the number of various types of rings in a molecule.
RotatableBond	A descriptor that counts the number and ratio of rotatable bonds in a molecule.
SLogP	A descriptor that estimates the octanol-water partition coefficient (LogP) and molar refractivity of a molecule.
TopoPSA	A descriptor that estimates the polar surface area of a molecule based on its topology.
TopologicalCharge	An index based on molecular topology that describes long-range charge transfer.
TopologicalIndex	An index describing topological features of a molecule, such as its diameter and radius.
VdwVolumeABC	The van der Waals volume of a molecule calculated based on the ABC method.
VertexAdjacencyInformation	Magnitude information describing the adjacency relationships of vertices in the graph.
WalkCount	A descriptor that counts the number of "walks" (non-self-avoiding paths) of a specific length in the molecular graph.
Weight	An index describing the molecular weight and average atomic weight.
WienerIndex	A topological index based on the sum of the shortest paths between all pairs of atoms in a molecule.
ZagrebIndex	An index that quantitatively measures molecular topology and branching based on atom degrees.

Supplementary Tab. 2 shows the number of fuel molecules of different categories in the database with different properties.

Supplementary Table 2. Detailed fuel categories and sample counts in the Constructed database. NA represents an empty value. EOV: enthalpy of vaporization, SEF: enthalpy of formation, VP: vapor pressure, ST: surface tension, FP: flash point, BP: boiling point, MP: melting point, RON: research octane number, MON: motor octane number, CN: cetane number, DCN: derived cetane number, LHV: lower heating value, UFL: upper flammability limit, LFL: lower flammability limit, YSI: yield sooting index.

Group	Compound Class	Physical Properties									Chemical Properties							
		Viscosity	EOV	SEF	VP	ST	FP	Density	BP	MP	RON	MON	CN	DCN	LHV	UFL	LFL	YSI
Hydrocarbons (HC)	n-Alkanes	17	20	17	29	26	32	28	31	32	5	5	19	10	32	17	32	8
	iso-Alkanes	16	64	39	41	62	64	85	86	71	42	41	78	18	69	39	59	22
	Alkenes	27	107	109	99	116	101	129	141	108	100	95	47	12	133	75	83	41
	Alkynes	1	5	18	11	10	16	23	22	21	8	4	1	NA	17	NA	10	3
Cyclic hydrocarbons (CHC)	Cycloalkanes	35	40	16	34	92	48	97	96	99	79	68	55	20	89	24	29	20
	Cyclic alkenes	2	10	4	10	9	13	15	19	15	19	19	15	1	17	12	11	12
	Bicycloalkanes	NA	NA	1	NA	NA	NA	NA	NA	NA	4	2	NA	NA	NA	NA	NA	NA
Aromatics (AROM)	Aromatics	29	60	26	76	120	132	162	161	163	41	43	49	14	162	104	123	121
	Terpenes	NA	NA	NA	NA	NA	NA	NA	NA	NA	2	NA	2	NA	NA	NA	NA	NA
Alcohols & Ethers (ALET)	Alcohols	34	88	47	110	19	154	135	158	129	25	18	51	17	123	87	110	50
	Acyclic ethers	14	37	14	38	8	55	68	68	48	7	7	46	16	51	31	44	29
	Cyclic ethers	NA	NA	NA	NA	NA	NA	NA	NA	NA	3	2	8	NA	NA	NA	NA	NA
	Other cyclic ethers	4	6	3	14	NA	13	12	14	12	NA	2	NA	5	12	2	9	3
Esters (ES)	Carbonate ester	1	2	1	4	NA	5	5	5	4	NA	NA	NA	NA	5	1	3	3
	Saturated esters	39	54	8	73	12	117	146	150	132	19	15	111	14	93	30	85	40
	Unsaturated esters	10	NA	2	21	3	41	46	46	29	NA	NA	56	3	27	16	24	21
Aldehydes & Ketones (ALKE)	Aldehydes	15	6	4	30	12	42	39	41	29	NA	1	8	7	36	25	35	18
	Cyclic ketone	3	2	1	6	2	8	10	9	8	3	2	5	4	7	2	7	2
	Ketones	14	26	8	32	12	42	50	50	41	10	9	18	7	31	17	29	28
Acids & Derivatives (Ac-X)	Carboxylic acids	15	7	9	27	8	57	39	50	54	NA	NA	9	NA	61	33	47	5
	Carboxylic anhydride	4	NA	2	6	NA	9	7	9	9	NA	NA	NA	NA	8	3	6	NA
	Amides	NA	NA	NA	NA	NA	NA	NA	NA	NA	1	NA	NA	NA	NA	NA	NA	NA

Continued on next page

Table 2 – continued from previous page

Group	Compound Class	Physical Properties									Chemical Properties							
		Viscosity	EOV	SEF	VP	ST	FP	Density	BP	MP	RON	MON	CN	DCN	LHV	UFL	LFL	YSI
Polyfunctionals (PolyF)	Polyfunctionals	25	14	5	51	8	90	75	86	55	5	3	38	21	66	56	58	14
Peroxides (POX)	Peroxide	NA	NA	1	NA	4	2	NA	2	NA	NA	NA	NA	NA	4	NA	3	NA
Epoxides (EPOX)	Furans	3	4	1	8	1	11	13	13	7	7	7	13	12	6	3	6	3
	Phenols	NA	NA	NA	NA	NA	NA	NA	NA	NA	1	NA	NA	NA	NA	NA	NA	NA

Supplementary Tab. 3 presents the sample counts of different fuel groups across six key property datasets (EOV, Viscosity, ST, RON, MON, and DCN) and the predictive R^2 performance of five models on each category. These results demonstrate that LG-Transformer effectively captures structure-property relationships and maintains robust predictive capabilities even under conditions of limited data availability.

To evaluate the quality of the learned embeddings, spearman's rank correlation coefficient is used to measure the monotonic relationship between the embedding space and the label space. For any pair of molecules m_i and m_j, consider their embeddings $\mathbf{e}_i$ and $\mathbf{e}_j$ and corresponding experimental attribute values y_i and y_j. The coefficient correlates the set of pairwise Euclidean distances $\{d_{ij} = \|\mathbf{e}_i - \mathbf{e}_j\|_2\}$ with the set of pairwise absolute label differences $\{\Delta y_{ij} = |y_i - y_j|\}$. It is formally calculated as follows:

$$\rho = \frac{\mathrm{cov}(rg(d_{ij}), rg(\Delta y_{ij}))}{\sigma_{rg(d_{ij})}\sigma_{rg(\Delta y_{ij})}}, \tag{1}$$

where $rg(\cdot)$ is the rank transformation, $\mathrm{cov}(\cdot,\cdot)$ denotes the covariance, and σ represents the standard deviation of the ranked variables. Based on this formula, the embedding quality of all datasets is tested under different property margins τ (as shown in Supplementary Tab. 4).

Supplementary Table 3. The performance comparisons of different models among six key fuel property datasets (EOV: enthalpy of vaporization, Viscosity, ST: surface tension, RON: research octane number, MON: motor octane number, and DCN: derived cetane number) for different fuel groups (HC: Hydrocarbons, CHC: Cyclic hydrocarbons, AROM: Aromatics, ALET: Alcohols & Ethers, ES: Esters, ALKE: Aldehydes & Ketones, PolyF: Polyfunctionals, EPOX: Epoxides, Ac-X: Acids & Derivatives) on representative folds. The table presents the R^2 averaged across all predicted properties, for the proposed LG-Transformer and four baseline models (Transformer, CatBoost, MolCLR, and AttentiveFP) on each fuel group. The 'Count' column indicates the total number of fuels within each group. For each fuel group, the highest R^2 among the models is highlighted in bold.

Fuel group	Count	Mean R^2				
		LG-Transformer	Transformer [2]	CatBoost [3]	MolCLR [4]	AttentiveFP [5]
HC	164	**0.982**	0.960	0.972	0.967	0.974
CHC	77	**0.955**	0.937	0.942	0.943	0.945
AROM	63	**0.992**	0.976	0.973	0.982	0.981
ALET	57	**0.966**	0.958	0.950	0.957	0.954
ES	36	**0.976**	0.965	0.960	0.968	0.972
ALKE	26	**0.984**	0.973	0.974	0.969	0.974
PolyF	18	**0.966**	0.945	0.956	0.945	0.965
EPOX	6	**0.996**	0.985	0.992	0.990	0.992
Ac-X	5	**0.960**	0.923	0.933	0.955	0.947

* The best-performing results are marked in bold.

Supplementary Table 4. Spearman's rank correlation coefficient results of contrastive learning performance on test data under varying τ parameters (fold 1). EOV: enthalpy of vaporization, SEF: enthalpy of formation, VP: vapor pressure, ST: surface tension, FP: flash point, BP: boiling point, MP: melting point, RON: research octane number, MON: motor octane number, CN: cetane number, DCN: derived cetane number, LHV: lower heating value, UFL: upper flammability limit, LFL: lower flammability limit, YSI: yield sooting index.

Dataset	Original	τ=2	τ=3	τ=4
SEF	0.3901	0.762	0.736	0.765
LHV	0.6026	0.763	0.848	0.825
RON	0.1445	0.340	0.570	0.570
MON	0.1723	0.512	0.567	0.520
CN	0.1634	0.377	0.486	0.489
YSI	0.4553	0.520	0.792	0.780
Density	0.4336	0.680	0.740	0.760
BP	0.4711	0.590	0.649	0.630
MP	0.3167	0.430	0.520	0.582
UFL	0.4123	0.598	0.610	0.579
LFL	0.4572	0.622	0.780	0.731
Viscosity	0.248	0.450	0.723	0.756
EOV	0.4916	0.720	0.842	0.866
VP	0.0235	0.585	0.731	0.758
DCN	0.2668	0.524	0.594	0.585
ST	0.3245	0.554	0.665	0.630
FP	0.4581	0.572	0.673	0.665

In addition, Supplementary Tab. 5 systematically studies the influence of neighbouring nodes in the feature fusion process through comparative experiments with different neighbour weight coefficients $\alpha_g \in \{0.1, 0.5, 1.0\}$.

Supplementary Table 5. Influence of different neighbor weights α_g on R^2. EOV: enthalpy of vaporization, SEF: enthalpy of formation, VP: vapor pressure, ST: surface tension, FP: flash point, BP: boiling point, MP: melting point, RON: research octane number, MON: motor octane number, CN: cetane number, DCN: derived cetane number, LHV: lower heating value, UFL: upper flammability limit, LFL: lower flammability limit, YSI: yield sooting index.

	Dataset	$\alpha_g = 0.1$	$\alpha_g = 0.5$	$\alpha_g = 1$
Physical Properties	Viscosity	0.894	0.882	0.871
	EOV	0.979	0.962	0.953
	SEF	0.940	0.932	0.915
	VP	0.928	0.901	0.895
	ST	0.835	0.808	0.812
	FP	0.931	0.939	0.906
	Density	0.947	0.942	0.925
	BP	0.852	0.855	0.831
	MP	0.796	0.784	0.773
Chemical Properties	RON	0.856	0.869	0.835
	MON	0.807	0.783	0.785
	CN	0.843	0.835	0.821
	DCN	0.842	0.834	0.819
	LHV	0.995	0.995	0.987
	UFL	0.917	0.893	0.892
	LFL	0.953	0.921	0.928
	YSI	0.982	0.979	0.959
MEAN		0.900	0.889	0.877

Supplementary Tab. 6 lists the hyperparameter search spaces for all baseline methods, which are taken from the original papers of these methods. Supplementary Tab. 7 lists the experiment settings for all the baseline methods.

Supplementary Table 6. Hyperparameter configuration for all baseline models. Baseline methods maintain parameter configurations from their original publications.

Model	Parameter	Value
CatBoost [3]	epochs	{1000, 1200, 1500}
	depth	{2, 4, 6}
	learning_rate	{0.01, 0.02, 0.05}
	l2_leaf_reg	{1.0, 3.0, 5.0}
MLP [6]	epochs	{1000, 1500, 2000}
	batch_size	{4, 8, 16, 32, 64, 128, 256}
	hidden_dim_1	{20, 40, 80, 160}
	hidden_dim_2	{20, 40, 80, 160}
	dropout_rate	[0.1, 0.4]
SVR [6]	C	{1, 10, 100, 500, 1000, 2000, 3000, 4000, 5000, 6000, 7000, 8000, 9000, 10000}
	epsilon	{0.1, 0.15, 0.2, 0.25, 0.3, 0.35, 0.4, 0.45, 0.5}
CNN [7]	kernel_sizes	{1,2,3,4,5,6,7,8,9,10,15,20}
	num_filters	{100,200,200,200,200,100,100,100,100,100,160,160}
Ridge(ML-QSPR) [8]	est_alpha	{1e-6, 1e-5, 1e-4, 1e-3, 1e-2, 1e-1, 1.0}
SGD(ML-QSPR) [8]	est_alpha	{1e-6, 1e-5, 1e-4}
	est_eta0	{0.001, 0.01}
KNN(ML-QSPR) [8]	est_n_neighbors	{3, 5, 7, 11}
	est_weights	{"uniform", "distance"}
RF(ML-QSPR) [8]	est_max_depth	{None, 10, 20, 30}
	est_min_samples_leaf	{1, 2, 4}
AttentiveFP [5]	radius	{2, 3, 4, 5, 6}
	T	{1, 2, 3, 4, 5}
	fingerprint_dim	[30, 300]
	weight_decay	[2, 6]
	learning_rate	[2, 5]
	p_dropout	[0, 0.5]

Supplementary Table 7. The experiment settings of all baseline models.

Model	Parameter	Value
CatBoost [3]	trials	50
	patience	50
MLP [6]	trials	50
	max_epochs	1500
	patience	150
SVR [6]	trials	50
CNN [7]	epochs	300
Chemprop [9]	epochs	300
AttentiveFP [5]	max_epochs	500
	patience	50
	hpo_max_iter	20
	hpo_init_evals	2
	hpo_seed	168
MolCLR [4]	num_layer	5
	emb_dim	300
	feat_dim	512
	drop_ratio	0.2
	epochs	300
ACS [10]	max-steps	10000
	val-interval	500
	learning-rate	1e-3
	weight-decay	1e-4
	conv-layers	8
	smoothing-alpha	0.1
KANO [11]	epochs	300

Supplementary Tab. 8 lists the hyperparameter space for LG-Transformer. Supplementary Tab. 9 lists the experiment settings for LG-Transformer.

Supplementary Table 8. Hyperparameter configuration of LG-Transformer.

Model	Parameter	Value
LG-Transformer	learning rate	[1e-4, 3e-3]
	feedforward dim	{512, 1024, 2048}
	dropout rate	[0.1, 0.5]
	weight decay	[1e-6, 1e-2]
	num_heads	{1, 2}
	num_layers	{1, 2, 3, 4}
	hidden_dim_1	{64, 128}
	hidden_dim_2	1024
	hidden_dim_3	{128, 256}

Supplementary Table 9. The experiment settings of LG-Transformer.

Model	Parameter	Value
Embedding	optimizer	Adam
	loss_function	$\mathcal{L}$ (as per Eq. (4) in the main text)
	learning rate	0.01
	dropout rate	0.5
	weight decay	1e-3
	hidden_dim_1	512
	hidden_dim_2	128
	epochs_embed	100
	bs_embed	512
	k	10
	δ	0.1
Transformer	optimizer	Adam
	scalar	{StandardScaler, LogTargetScaler}
	loss_function	MSE
	trials	50
	epochs_tr	500
	patience	50
	bs_tr	256
	exp_k	10

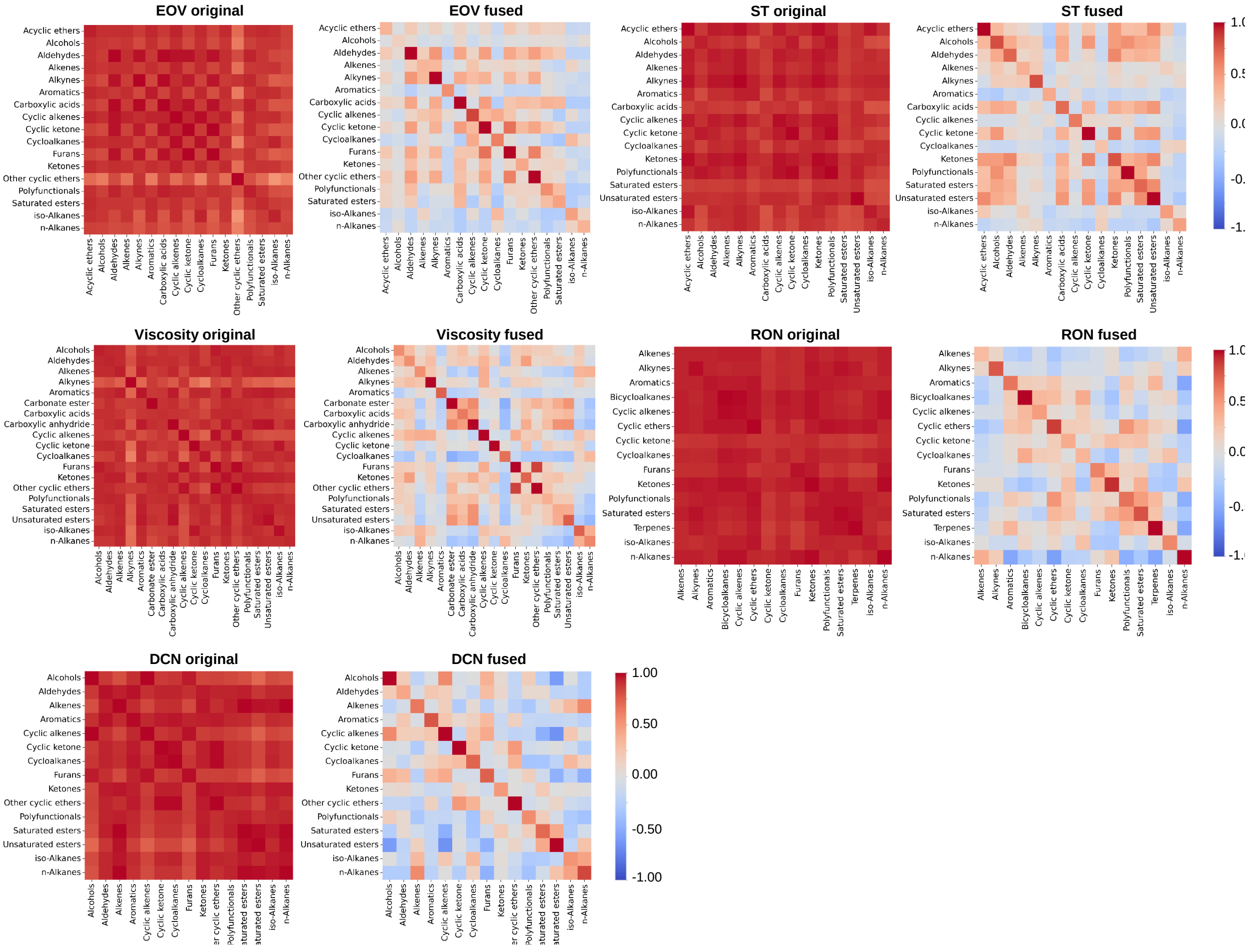

Supplementary Figure 1. Inter-class similarity heatmap of key properties.

2. Supplementary Figures

Supplementary Fig. 1 complements the analysis in the main text by providing a comparative visualization of inter-class similarity matrices for five key properties before ('Original') and after ('Fused') learned-graph feature fusion. Each heatmap displays the cosine similarity between the mean molecular representations of different fuel classes.

Supplementary Fig. 2 supplements the analysis in the main text by displaying the 2D t-SNE [12] embeddings of molecular representations for five key properties. Each plot represents individual compounds as points, colored by their respective fuel class, offering a side-by-side comparison of the feature space before ('Original') and after fusion ('Fused').

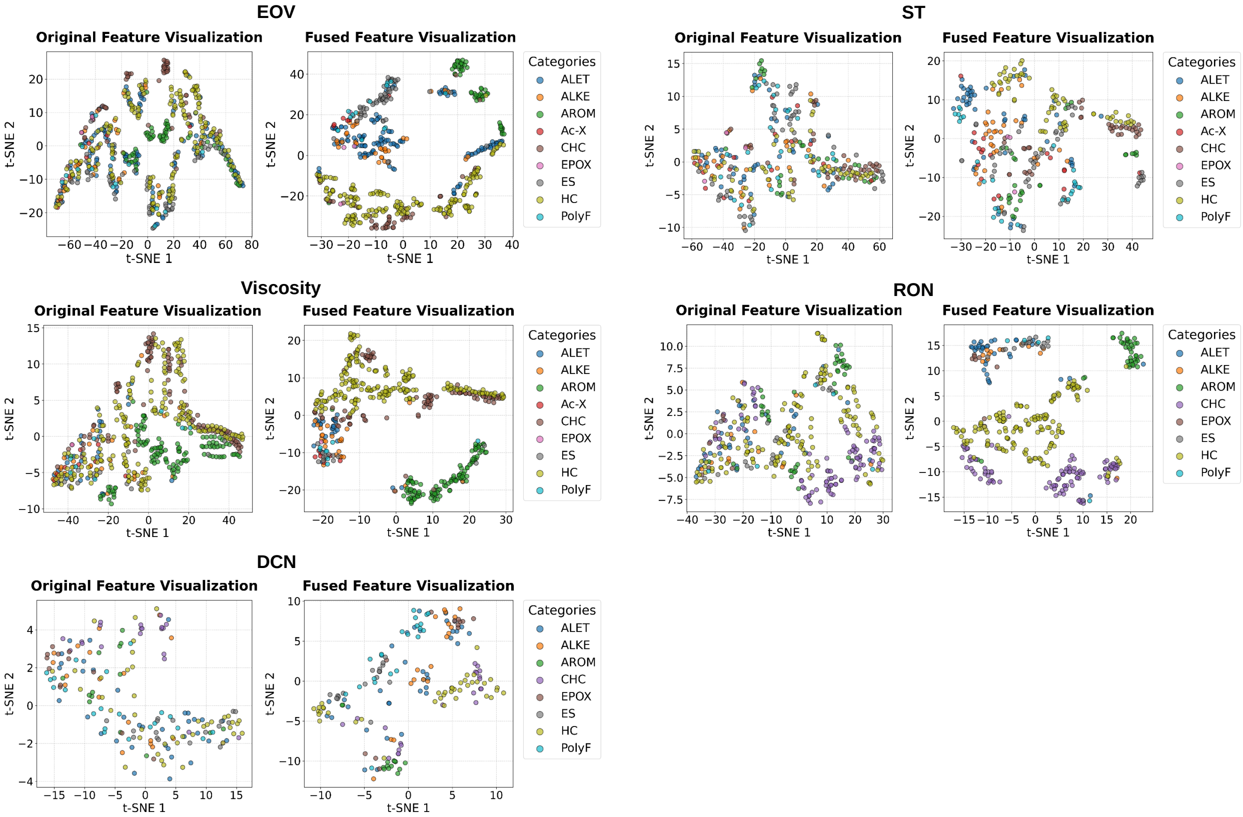

Supplementary Figure 2. Scatter plot of key properties after dimensionality reduction using t-SNE.

Supplementary Fig. 3 provides a comparative visualization of the feature space topology in relation to continuous property values for five key properties. It offers a side-by-side comparison before ('Original') and after ('Fused') feature fusion. In each plot, molecular representations are projected into a 2D t-SNE space, and each point is colored according to its true property value. To quantitatively evaluate the structural coherence of the molecular feature space with respect to target properties, a spatial continuity metric is introduced. For each molecule m_i represented by its feature vector $\mathbf{x}_i$ and property value y_i, the local continuity score c_i for molecule m_i is computed as:

$$c_i = \frac{1}{|\mathcal{N}_k(m_i)|} \sum_{m_j \in \mathcal{N}_k(m_i)} \frac{1}{1 + |y_i - y_j|}, \tag{2}$$

where $|\mathcal{N}_k(m_i)|$ denotes the cardinality of the neighborhood set. This formulation measures the average property similarity between m_i and its neighbors, with values approaching 1 indicating perfect local consistency. The global spatial continuity metric C is then defined as the mean of all local continuity scores:

$$C = \frac{1}{n} \sum_{i=1}^{n} c_i, \tag{3}$$

where n represents the total number of molecules. This comprehensive metric ranges from 0 to 1, with higher values indicating superior alignment between the topological organization of the feature space and the underlying property distribution.

Supplementary Fig. 4 evaluates the predictive accuracy of the LG-Transformer model across all physicochemical properties on the test set. Each scatter plot compares the predicted values (y-axis) with the actual experimental values (x-axis). The blue points represent individual data points, the solid green line is their linear regression fit, and the red dashed line indicates the ideal prediction (y=x) where these values are equal.

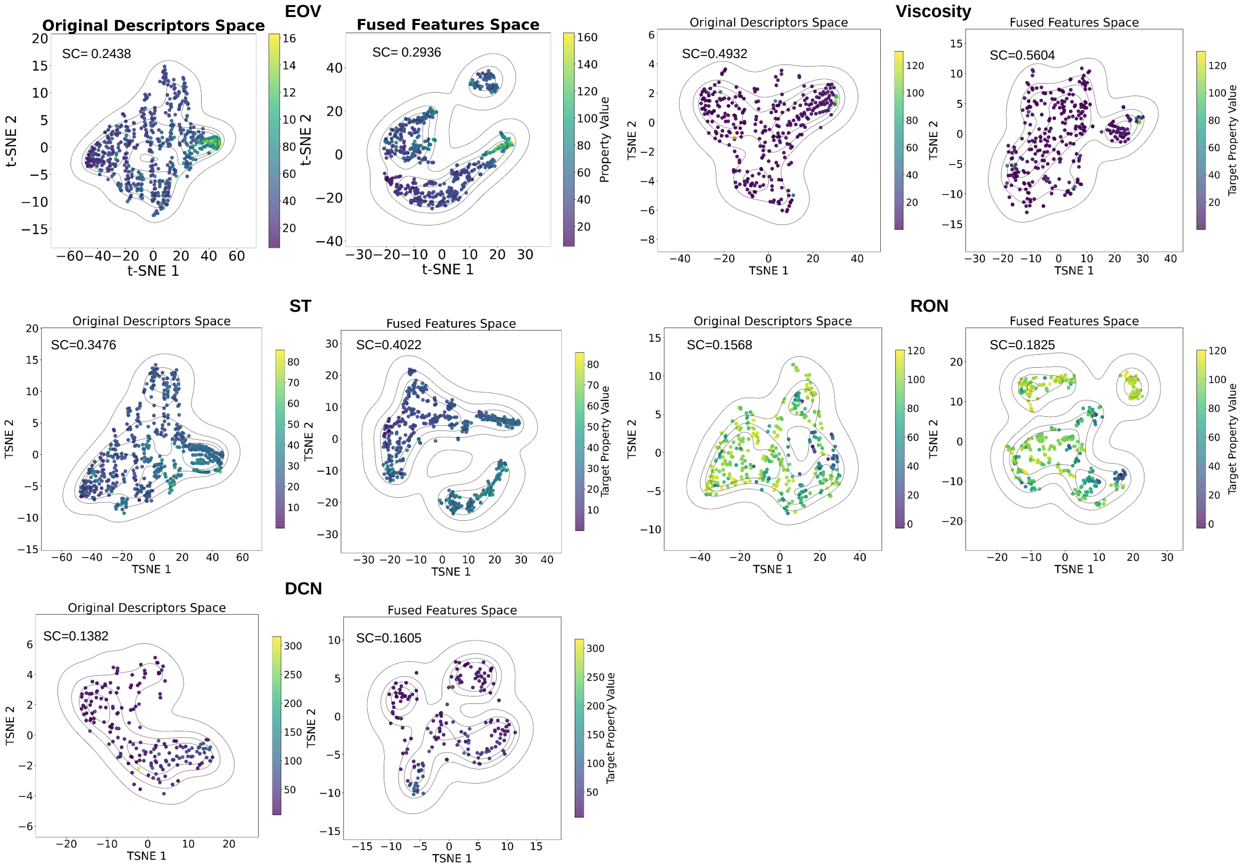

Supplementary Figure 3. Scatter plot of property colouring after dimension reduction of key properties using t-SNE.

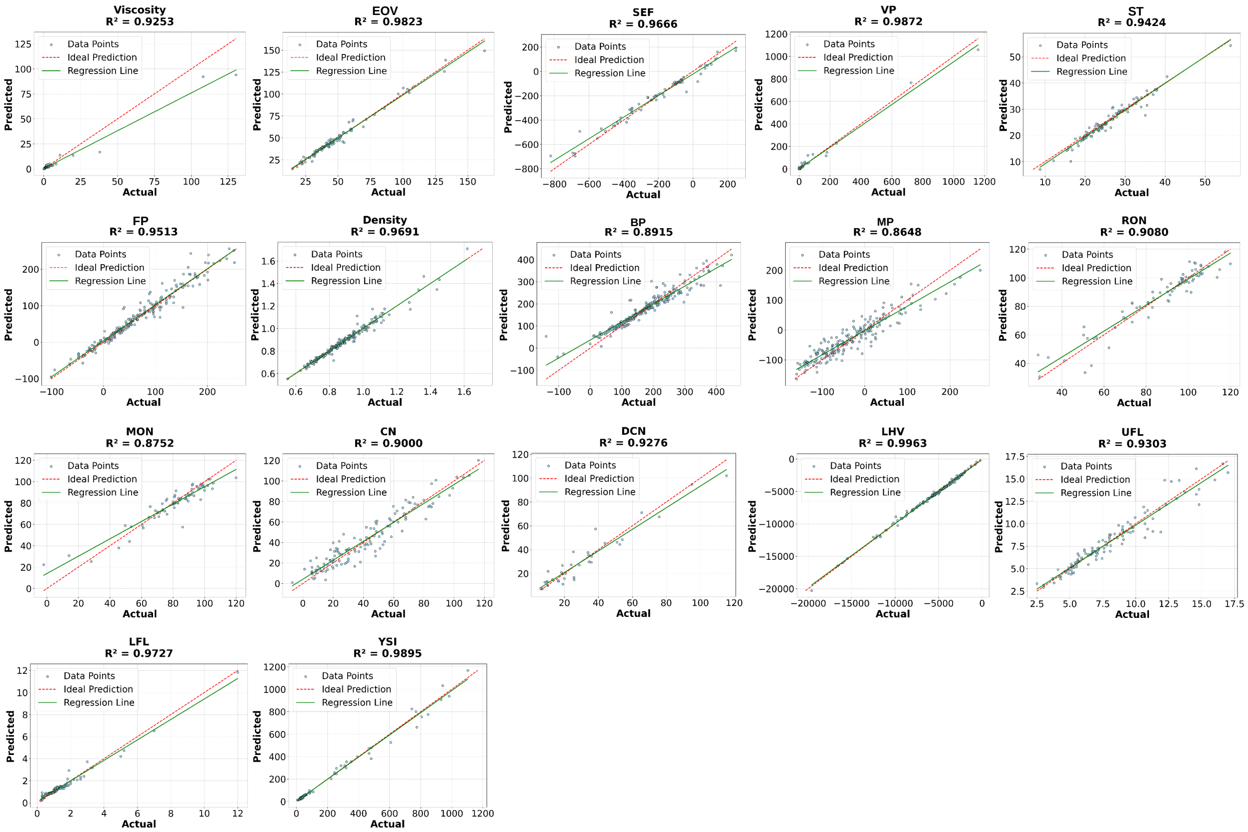

Supplementary Figure 4. Regression Fit Scatter Plot for all properties.

Supplementary Fig. 5 presents a sample-by-sample comparison between the predicted values of LG-Transformer (red line) and the actual experimental values (blue line) for all properties of the test set. The x-axis represents the index of each compound in the test set, while the y-axis represents the property value.

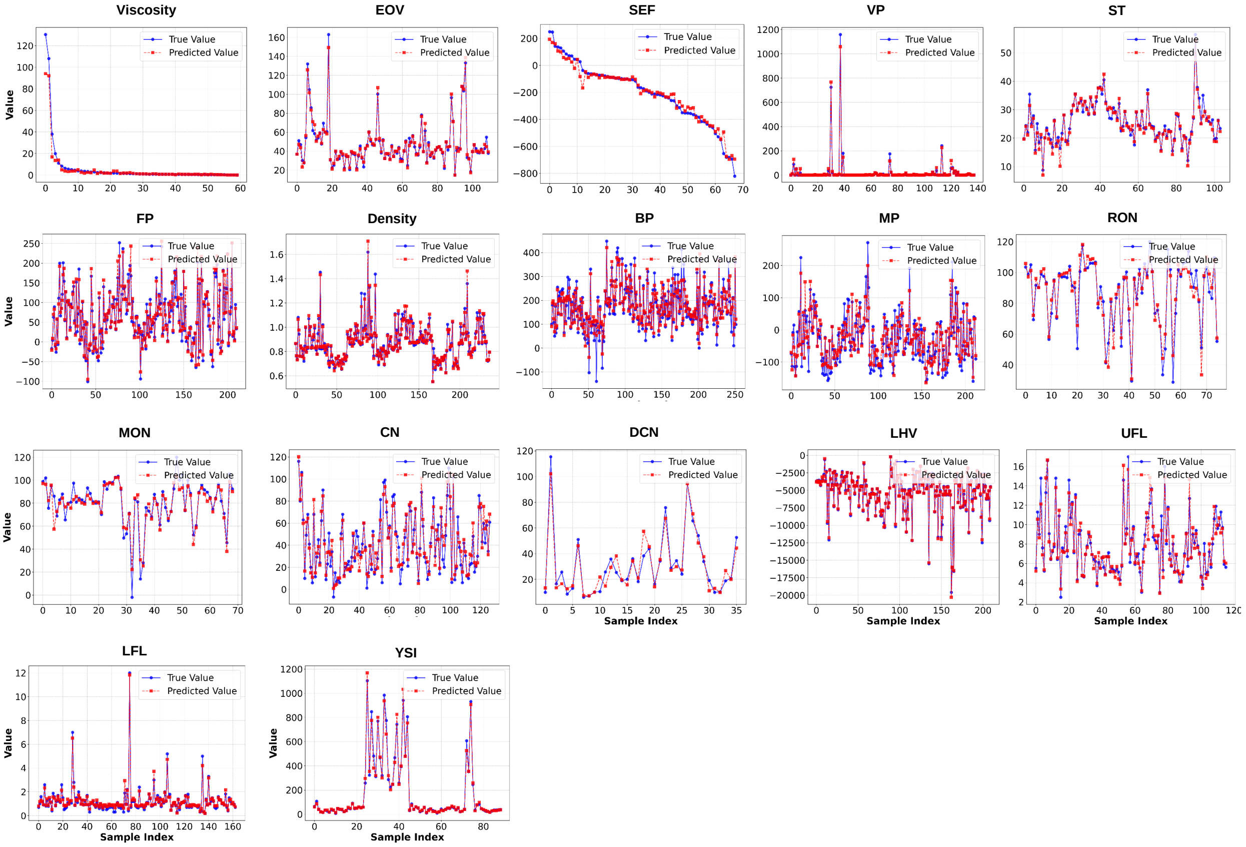

Supplementary Figure 5. Actual vs. Predicted Scatter Plot for all properties.

Supplementary Fig. 6 visualizes the learned attention score matrices from the Transformer Encoder for all property prediction tasks. Each heatmap illustrates the relationships between different groups of molecular descriptors. The y-axis ('Query') represents the descriptor group attending, and the x-axis ('Key') represents the group being attended to. The color intensity of each cell corresponds to the magnitude of the attention score, with brighter colors (yellow) indicating stronger interactions.

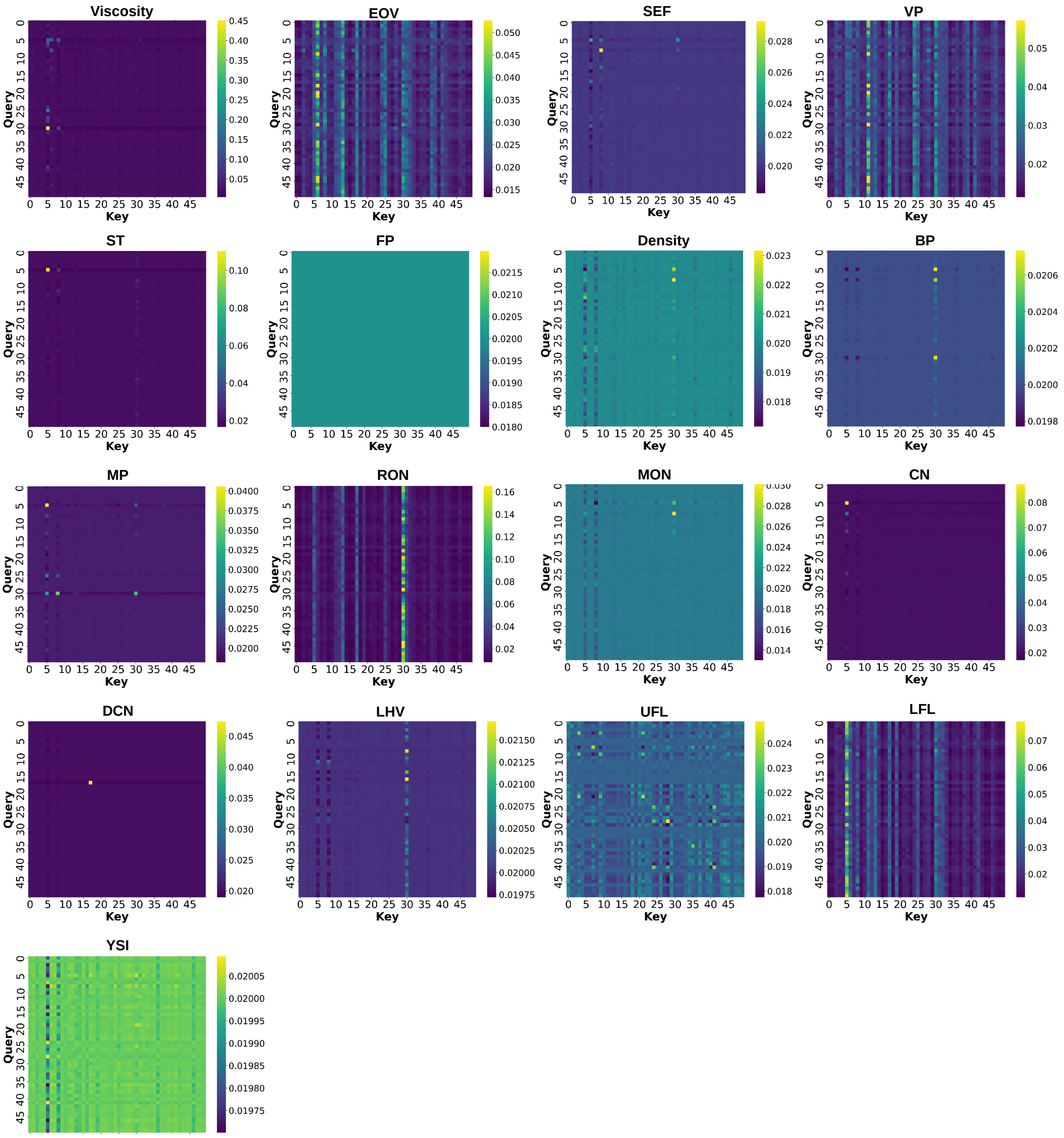

Supplementary Figure 6. Heatmap of attention scores for all properties.

Integrated Gradients [13] is a gradient-based model interpretation technique, grounded in the core hypothesis that prediction variations can be decomposed into cumulative gradient contributions of input features along a path from a baseline input to the actual input. The input to the model for a single sample is the categorized molecular descriptor matrix

$\tilde{\mathbf{x}} \in \mathbb{R}^{k_s \times k_d}$. This matrix is composed of k_s descriptor categories, where the i-th category is represented by the vector $\mathbf{c}_i \in \mathbb{R}^{k_d}$ (which can be considered the i-th row of $\tilde{\mathbf{x}}$). Similarly, a baseline input matrix is defined as $\tilde{\mathbf{x}}' \in \mathbb{R}^{k_s \times k_d}$, with its i-th category vector being $\mathbf{c}'_i \in \mathbb{R}^{k_d}$. The IG attribution for the i-th descriptor category vector $\mathbf{c}_i$ is then computed as:

$$IG(\mathbf{c}_i) = (\mathbf{c}_i - \mathbf{c}'_i) \cdot \int_{\alpha_{in}=0}^{1} \frac{\partial f(\tilde{\mathbf{x}}' + \alpha_{in}(\tilde{\mathbf{x}} - \tilde{\mathbf{x}}'))}{\partial \mathbf{c}_i} d\alpha_{in}, \tag{4}$$

where $(\mathbf{c}_i - \mathbf{c}'_i)$ is the difference vector for the i-th category. The term $\frac{\partial f(\cdot)}{\partial \mathbf{c}_i}$ represents the gradient of the output of the model with respect to the category vector $\mathbf{c}_i$. α_{in} denotes the interpolation coefficient that controls the path from baseline $\mathbf{c}'_i$ to actual input $\mathbf{c}_i$, while the integral term represents the cumulative contribution of the gradient along this route. The zero vectors are adopted as baselines, and the contribution values across all samples are computed for all property predictions, ultimately averaged to generate the contribution histograms shown in Supplementary Fig. 7. In each bar chart, the x-axis represents the index of the descriptor group, and the y-axis shows its total contribution to the final prediction. A positive bar indicates that the descriptor group contributes to increasing the predicted property value, while a negative bar indicates a decreasing contribution.

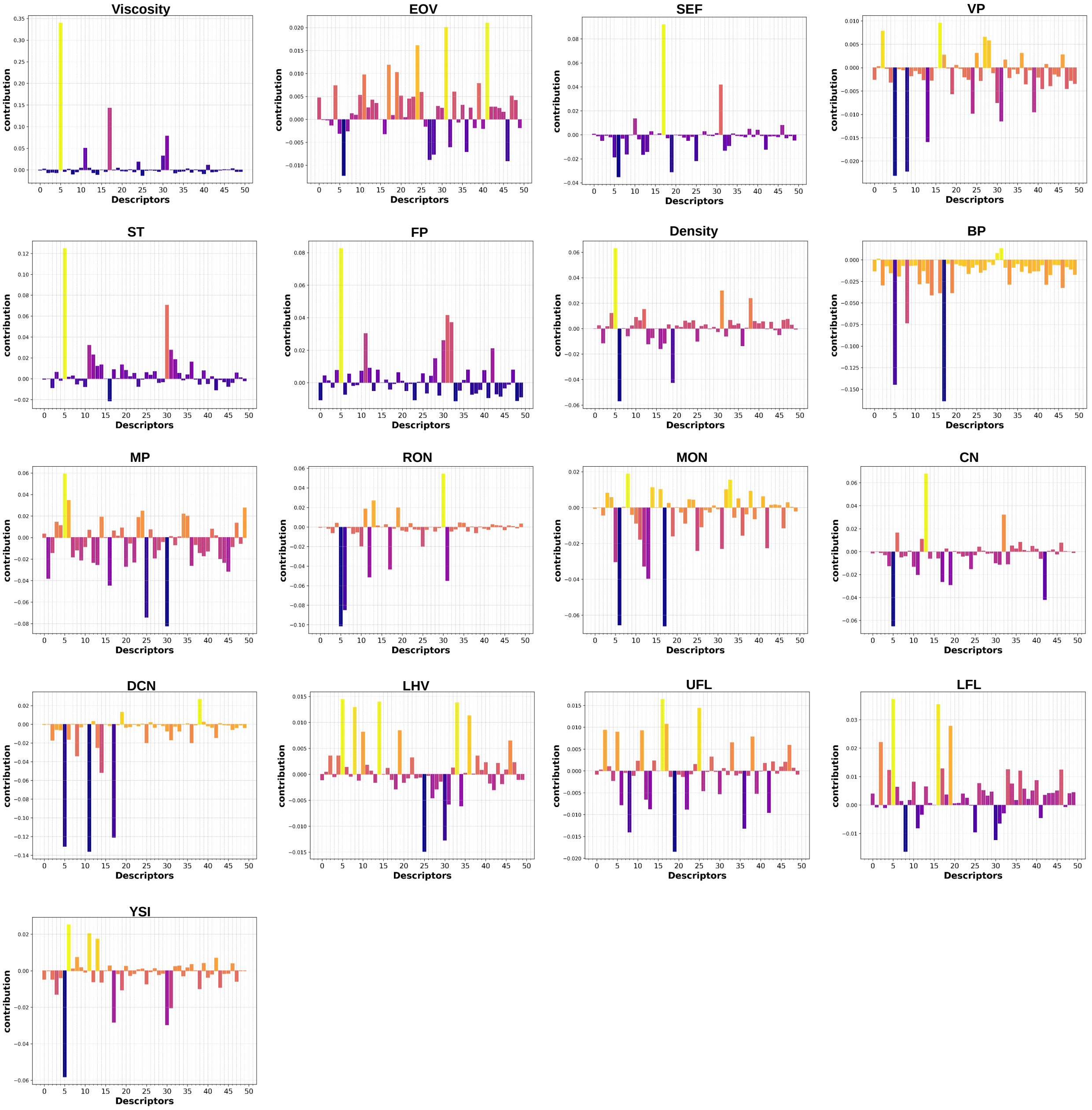

Supplementary Figure 7. IG scores for all properties.

The graph presented in Figure 8 compares the prediction accuracy of different models—including LG-Transformer, Transformer, CatBoost, and MLP—during training on the HOV dataset across various epochs (with a batch size of 128). The x-axis represents the time (in epoch) required for training, while the y-axis indicates the R^2 values, reflecting the

models' prediction accuracy. LG-Transformer demonstrates a superior balance, converging to the highest R^2 score (0.97) in approximately 290 epochs. In contrast, the standard Transformer model achieves the highest performance ($R^2 = 0.95$) after approximately 330 epochs. However, MLP requires nearly 350 epochs to converge to the optimal performance ($R^2 = 0.93$). The number of epochs required for Catboost to converge is much larger (about 1000 epochs), and the best performance is also worse ($R^2 = 0.94$).

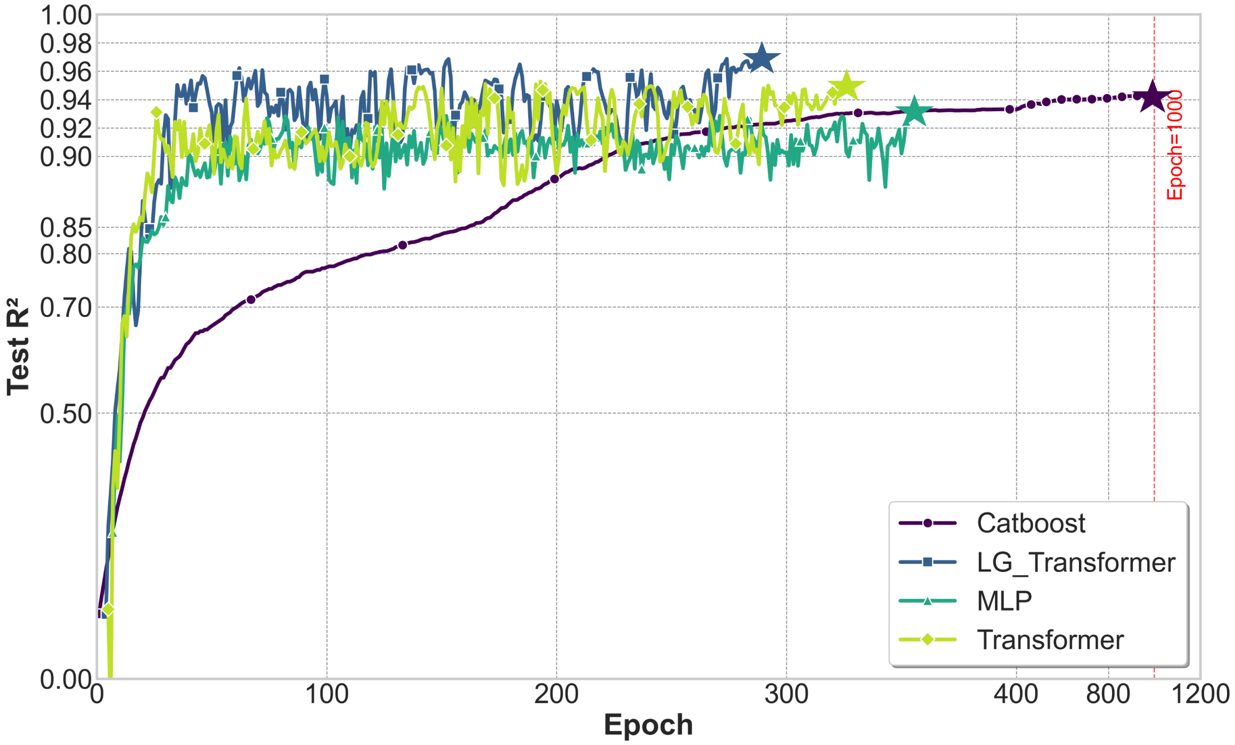

Supplementary Figure 8. Graph showing the variation in computational cost and prediction accuracy for different models.

Supplementary References

[1] H. Moriwaki, Y.-S. Tian, N. Kawashita, T. Takagi, Mordred: A molecular descriptor calculator, Journal of Cheminformatics 10 (1) (2018) 4.

[2] A. Vaswani, N. Shazeer, N. Parmar, J. Uszkoreit, L. Jones, Attention is all you need, in: Advances in Neural Information Processing Systems, Vol. 30, Curran Associates, Inc., 2017, pp. 5998–6008.

[3] S. S. Nagaraja, S. M. Sarathy, B. Mohan, J. Chang, Machine learning-driven screening of fuel additives for increased spark-ignition engine efficiency, Proceedings of the Combustion Institute 40 (1-4) (2024) 105658.

[4] Y. Wang, J. Wang, Z. Cao, A. Barati Farimani, Molecular contrastive learning of representations via graph neural networks, Nature Machine Intelligence 4 (3) (2022) 279–287.

[5] Z. Xiong, D. Wang, X. Liu, F. Zhong, X. Wan, X. Li, Z. Li, X. Luo, K. Chen, H. Jiang, M. Zheng, Pushing the boundaries of molecular representation for drug discovery with the graph attention mechanism, Journal of Medicinal Chemistry 63 (16) (2020) 8749–8760.

[6] K. K. Yalamanchi, V. C. O. van Oudenhoven, F. Tutino, M. Monge-Palacios, A. Alshehri, X. Gao, S. M. Sarathy, Machine learning to predict standard enthalpy of formation of hydrocarbons, The Journal of Physical Chemistry A 123 (38) (2019) 8305–8313.

[7] P. Karpov, G. Godin, I. V. Tetko, Transformer-cnn: Swiss knife for qsar modeling and interpretation, Journal of Cheminformatics 12 (1) (2020) 17.

[8] R. Li, J. M. Herreros, A. Tsolakis, W. Yang, Machine learning-quantitative structure property relationship (ml-qspr) method for fuel physicochemical properties prediction of multiple fuel types, Fuel 304 (2021) 121437.

[9] E. Heid, K. P. Greenman, Y. Chung, S.-C. Li, D. E. Graff, F. H. Vermeire, H. Wu, W. H. Green, C. J. McGill, Chemprop: A machine learning package for chemical property prediction, Journal of Chemical Information and Modeling (2023).

[10] B. A. Eraqi, D. Khizbullin, S. S. Nagaraja, S. M. Sarathy, Molecular property prediction in the ultra-low data regime, Communications Chemistry 8 (1) (2025) 201.

[11] Y. Fang, Q. Zhang, N. Zhang, Z. Chen, X. Zhuang, X. Shao, X. Fan, H. Chen, Knowledge graph-enhanced molecular contrastive learning with functional prompt, Nature Machine Intelligence 5 (5) (2023) 542–553.

[12] L. Van der Maaten, G. Hinton, Visualizing data using t-sne., Journal of machine learning research 9 (11) (2008).

[13] M. Sundararajan, A. Taly, Q. Yan, Axiomatic attribution for deep networks, in: Proceedings of the 34th International Conference on Machine Learning, Vol. 70 of Proceedings of Machine Learning Research, PMLR, 2017, pp. 3319–3328.
