# Supplementary material for: LG-Transformer: learned-graph transformer framework enabling diverse physicochemical properties prediction toward fuel design
Source: Nat Commun. 2026 Jun 3;17:7137. doi: 10.1038/s41467-026-73853-z (PMC13396237; doi:10.1038/s41467-026-73853-z)
Supplement: Supplementary file 2 — Transparent Peer Review file [file 41467_2026_73853_MOESM2_ESM.pdf]

nature portfolio

Peer Review File

LG-Transformer: Learned-Graph Transformer Framework Enabling Diverse Physicochemical Properties Prediction toward Fuel Design

Corresponding Author: Professor Peng Han

This file contains all reviewer reports in order by version, followed by all author rebuttals in order by version.

Version 0:

Reviewer comments:

Reviewer #1

(Remarks to the Author)
Review of Transformer: Learned-Graph Transformer Framework Enabling Multi-Objective Physicochemical Property Prediction toward Fuel Design

The manuscript introduces LG-Transformer, a hybrid framework that (i) learns a property-aware graph via contrastive learning on descriptor embeddings, (ii) propagates features with a GCN, and (iii) models inter-descriptor dependencies with a Transformer encoder to predict 17 physicochemical properties for 1,850 molecules spanning 26 classes. The authors assemble a new database and report an average $R^2 \approx 0.933$ with interpretability analyses based on integrated gradients (IG). The problem is relevant, the dataset curation is substantial, and the architecture is well-motivated. However, several major issues around problem framing (multi-objective vs. single-task), validation scheme, evaluation design, dataset curation should be addressed.

Strengths
The motivation and model architecture are clear. The framework is built on an intuitive pipeline that injects relational inductive bias.
The curated data are exposed via an open website and Figshare. The code is well organized. It’s good for reuse.
The authors conducted ablation studies to show each module’s contribution, demonstrating the effectiveness of the model architecture.

Comments

It is unclear what the authors refer to by “multi-objective.” If this means utilizing a unified backbone (i.e., shared parameters) with task-specific heads (i.e., multi-task learning, MTL), then it is important to quantify the advantage of MTL relative to single task learning through ablations. It is also important to adopt the naming convention used within ML and molecular property prediction domains (multi-task learning) to avoid confusing the readers.
Tab. 1 shows many NA entries and highly uneven counts across classes and properties (e.g., bicycloalkanes, terpenes). This shows that the collected dataset has problems with label missingness or imbalance. The imbalance also exists where some large families (e.g., aromatics, alkenes) dominate counts versus sparse families. Do these risks bias training and unstable estimates for low-data cells?

Lack of comparison with comparable and state-of-the-art (SOTA) deep learning models for molecular property prediction. While the authors follow a mostly standard procedure for baselining, they compare only to classical techniques (boosting, SVR), and non-graph based methods (descriptors-based MLP implementation and a generic transformer). A fairer comparison should include models established for molecular property prediction applications on multiple properties, including ones based on molecular graphs. Examples include D-MPNN/Chemprop [1], ML-QSPR [2] , Attentive FP [3], and ACS [4]. Also important to gauge performance against SOTA contrastive learning models, such as [5], [6]and others.

Beyond the authors' curated dataset, evaluation on standard molecular property datasets (e.g., MoleculeNet [7]) would provide a better basis for comparison and position this work relative to existing literature

To verify the generalizability of the model, it is paramount to evaluate its performance on k-fold cross-validation training split or some of its variations. The traditional train/test split is known to overestimate model performance, especially with the lack of an unseen validation set which allows overfitting on the test data. Moreover, the hyperparameter optimization process and corresponding parameter search space are not clearly described in the manuscript. To ensure fair performance reporting, the authors should evaluate the model performance on k-fold cross-validation or a Murcko-scaffold split [8]. This also serves to demonstrate the stability of the training technique and robustness to random parameter initializations.

Lack of detailed information about training dataset curation. The data sources provided on https://ai4fuel.sjtu.edu.cn/ show datasets known to contain predicted property values (e.g., DIPPR 801). It is important to train the models solely on experimental and rigorously derived fuel property values. A table of units and temperatures for every property, and how data drawn from multiple sources was standardized is necessary. In the supplementary data, it is important to include the data source of each fuel property value. Moreover, to facilitate results reproduction, please include a script in the code repository to enable quick training and result validation.
Units of Mean Absolute Error values are missing in Table 2. The readers cannot discern the significance of reported MAEs if the physical unit is missing (e.g.; 1.3 Pa v.s. 1.3 kPa for VP in Table 2). Moreover. aggregation of MAEs across properties with different units (i.e., MAE 13.12 in Table 2) is not meaningful and can misguide the reader into thinking it can be used as a universal metric to compare across models. Average R2 is a better choice.
Splitting is performed per property, implying varying sample sets per target; cross-property correlations may be underexploited and evaluation comparability across targets can be tricky. Do authors consider this in contrastive learning?
The dataset is collected from heterogeneous sources, so it's important to spell out the specific property units, detailed unit harmonization, conflict resolution, quality control, and outlier handling procedures in the main text or caption of Fig. 1.
Are measurement uncertainties recorded or retrievable from the cited sources? Authors should estimate and report the uncertainty for each property in the dataset to enable usage of the curated dataset.
In contrastive learning, negatives are defined as label-dissimilar yet feature-close samples. Early in training most pairs may be far in embedding space; how are sufficient negatives found or updated across epochs? I think more detail on the mining schedule would help.
In Methods, Norm(G) is introduced but not defined (row-norm? sym-norm? softmax over neighbors?). This choice would affect stability and scaling; please detail it.
GCN propagation depth/hops: the authors seem to use a single aggregation with $\tilde{A}$ in eq. (9). Did the authors try deeper propagation (e.g., stacked GCN layers) and observe over-smoothing or gains? Please clarify the design choice.
About the descriptor-token formation details, when building the $ks \times kd$ token grid: how are missing descriptors handled, are groups padded/masked, and is token order fixed across molecules? Mordred can yield NaNs. The paper does not state whether NaNs are imputed (mean/median/zero), dropped, or handled via a learned "missing" mask/channel. Standardization alone is insufficient context here because it presupposes finite values. This should be discussed. In addition, descriptor groups differ in size, yet the Transformer requires a fixed per-token width kd. The authors should describe which strategies are used here (e.g. padding, masking, pooling, or projection).
About the baselines, the paper compares the LG-transformer against traditional ML (SVR, MLP, CatBoost), a descriptor-only Transformer, and CNN, however, authors should also include some modern, strong molecular baselines, such as D-MPNN, Graphormer, MolFormer, ChemBERTa, etc. As written, the baseline roster is not state-of-the-art for molecular ML, which undercuts the "SOTA" claim.
Only random class-stratified splits are used. Since the dataset includes a range of fuel families, it is suggested that scaffold and leave-class-out splits should be reported. Random splits often overestimate performance. Authors should consider these splits enforcing structural dissimilarity or extrapolation across fuels to mirror real deployment of fuel design.
In ablation studies, because the graph is learned with label-defined positives, the authors should explicitly explain split-safe construction in detail to prevent leakage influencing ablation results. For example, in the "-contrastive" setting, replace the learned embedding with a label-free alternative and avoid building any graph from label distances; that would be direct leakage.
The authors mention MSE and MSLE loss functions. Please clarify which tasks use which loss and why, and whether losses were task-specific in a multi-task setting.

References
[1] E. Heid et al., "Chemprop: A Machine Learning Package for Chemical Property Prediction," J Chem Inf Model, 2023, doi: 10.1021/acs.jcim.3c01250.
[2] R. Li, J. M. Herreros, A. Tsolakis, and W. Yang, "Machine learning-quantitative structure property relationship (ML-QSPR) method for fuel physicochemical properties prediction of multiple fuel types," Fuel, vol. 304, p. 121437, 2021, doi: https://doi.org/10.1016/j.fuel.2021.121437.
[3] Z. Xiong et al., "Pushing the Boundaries of Molecular Representation for Drug Discovery with the Graph Attention Mechanism," J Med Chem, vol. 63, no. 16, pp. 8749–8760, Aug. 2020, doi: 10.1021/acs.jmedchem.9b00959.
[4] B. A. Eraqi, D. Khizbullin, S. S. Nagaraja, and S. M. Sarathy, "Molecular property prediction in the ultra-low data regime," Commun Chem, vol. 8, no. 1, p. 201, Jul. 2025, doi: 10.1038/s42004-025-01592-1.
[5] Y. Wang, J. Wang, Z. Cao, and A. Barati Farimani, "Molecular contrastive learning of representations via graph neural networks," Nat Mach Intell, vol. 4, no. 3, pp. 279–287, 2022.
[6] Y. Fang et al., "Knowledge graph-enhanced molecular contrastive learning with functional prompt," Nat Mach Intell, vol. 5, no. 5, pp. 542–553, 2023, doi: 10.1038/s42256-023-00654-0.
[7] Z. Wu et al., "MoleculeNet: A Benchmark for Molecular Machine Learning," Chem Sci, vol. 9, Oct. 2017, doi:

10.1039/C7SC02664A.
[8] G. W. Bemis and M. A. Murcko, “The Properties of Known Drugs. 1. Molecular Frameworks,” J Med Chem, vol. 39, no. 15, pp. 2887–2893, Jan. 1996, doi: 10.1021/jm9602928.

(Remarks on code availability)

Reviewer #2

(Remarks to the Author)
Elements of a reviewer report
Key results
This manuscript describes a new machine learning approach for the prediction of mono fuel properties (physical and chemical) for the evaluation of new fuels. The novel approach is a two-stage approach to representation learning to improve the prediction accuracy for multiple properties using the same architecture.
The manuscript is well-written and well structured.
Validity
The manuscript well describes the method used and the logic behind the approach. The authors also describe well the training and optimization procedure for the neural network and validation method of the LG-transformer.
One minor comment: I see that the authors have used an optimizer algorithm to optimize their neural network – if it has not been done it always sound to read up on “rules of thumbs” on ranges for the optimization parameters depending on the spread of data and number of data points used in the training data sets, and see if these are aligned with those of results of the optimization algorithm.
What could improve the validity of the LG-transformer is:
1. A list of number of data points used for training and validation for each individual property. I know from experience that some property data is easier to find or measure than other. It should be transparent how much training data is available for the neural network.
2. Adding a final validation after the model is defined with random datapoints from different property datasets that was not incorporated in the initial training and validation. This can help spotting any optimization issues causing the model to be less general for future predictions on untrained input.
3. One important thing is that the model can capture the behavior in the predictions. For example, it is briefly discussed in the Discussion section that the alkanes show lower octane number and higher cetane number, which is expected, but how did the model capture the behavior of other molecules?
Or how did it behave when multiple functional groups where present in one molecule?
On the same subject it is also interesting to discuss if there where ranges or molecules where the model performed worse and if so if these are ranges/molecules important for fuel evaluation or not.
This can be done with showing the full predictions (predicted value vs measured value) in a few graphs also showing important outliers (if any).
Significance
The use of various machine learning or neural network methods to more accurately predict chemical or physical properties has been a hot topic in both chemical and mechanical engineering over the last few years. The methods are getting better as the knowledge on ML and ANNs increase over time. For fuel property prediction it is an interesting approach, but I think the significance of accurate models are higher for multi-component fuels over single-component fuels (as discussed in this paper). However, there is significance of this type of work also for other applications to improve property prediction and not only for fuel evaluation. Perhaps a wider scope than just for fuel evaluation could lead to a higher significance of this paper.
Data and methodology
In general the data and methodology is well described and documented. However, some elements are described in more detail than others. This cause a bit of confusion for the reader as some sections include a lot of details while other topics are only briefly discussed.
The description on how the LG-transformer is used to predict different properties is unclear to me, and only mentioned briefly. The authors should better describe which properties are predicted together and if methods differ between the prediction of different properties (perhaps in a schematic?).
If all properties are used for training and prediction it is important to note how the authors have dealt with properties where one node might give positive impact for one property but, for another property negative impact.
The presentation of the data could also benefit from a more detailed comparison of the approach described in the manuscript and the approach in other papers (e.g. comparing R2 and root mean square error (RMSE) with the results from models in other publications (many already included in the reference list).
Even if the authors of those papers focused on one property only, it is important to clearly show the benefits of the presented model in comparison to already published models in a clear and transparent way.
I am also missing a short discussion on computational cost versus accuracy of predictions from the authors.
Analytical approach
The analytic approach is good. For an even better discussion of the results I would advise the authors to include the three discussion points mentioned under “Validity”.
Suggested improvements
The improvements mentioned under the other headings include all my suggested improvements.
Clarity and context
The authors provide enough information for the paper to be clear to the reader and for the model and methodology to be well-described. A more detailed and transparent comparison to previous work on the same topic could improve the

manuscript in relation to consideration of previous work.
All the necessary references are available in the reference list, the authors can use the information in those papers to compare model performances as it would better show the novelty and progress of the LG-transformer compared to previous efforts.
References
I think that the paper includes enough and relevant references to the work.

(Remarks on code availability)

Reviewer #3

(Remarks to the Author)
This manuscript presents a novel deep learning framework (LG-Transformer) for the simultaneous prediction of multiple key physicochemical properties of fuel molecules. The core contribution lies in integrating contrastive learning with Graph Convolutional Networks (GCN) and Transformer to construct a "property-aware" molecular relationship graph that enhances molecular feature representation. The authors have also compiled a high-quality dataset of 1850 molecules across 26 classes and 17 properties, which is a valuable resource in itself. The experimental design is thorough, and the results clearly demonstrate that the proposed method achieves state-of-the-art (SOTA) performance on the vast majority of prediction tasks. The paper is well-written and clearly structured. I think this manuscript is suitable for publication in this journal after proper revisions. Below are my specific comments, questions, and suggestions aimed at helping the authors further strengthen the manuscript.
1. Dataset Partitioning and Experimental Rigor: The data partitioning strategy described in the paper (Section 2.1 and Fig. 2a) involves a simple 80%/20% stratified split into a training set and a test set. All subsequent model comparisons, hyperparameter optimization (using Optuna), and model selection are based on this single split. This constitutes a major methodological flaw. Hyperparameter optimization (HPO) is inherently a form of model selection based on performance. Using the test set to guide HPO leads to data leakage, as information from the test set implicitly influences the model building process. This means the test set is no longer a truly "unseen" dataset, and the reported performance metrics (R^2, MAE) become optimistically biased estimates that do not generalize to new data.
2. Clarity on Novelty and Core Contribution: The paper's core innovation is the use of contrastive learning to build a property-guided molecular graph, which is then used for GCN feature propagation. This approach is ingenious and effective. However, the description of this core innovation in the "Introduction" and "Abstract" could be more prominent. It is currently somewhat generically described as "integrates contrastive learning...". I recommend more explicitly stating that unlike common GNNs based on molecular structural graphs (e.g., atom-bond graphs), the "graph" in this work is an inter-molecular relationship graph based on property similarity, which is a fundamental distinction and contribution.
3. Justification for the Contrastive Learning Component: The ablation study shows that removing the contrastive module (-Contrastive) leads to a performance drop, proving its effectiveness. However, a critical question arises: What are the advantages of using contrastive learning to indirectly learn an embedding for graph construction, compared to a more direct approach of using the property labels themselves (or the inverse of property differences) as edge weights for the graph? Is the benefit better generalization, increased robustness to noise, or something else? The authors should briefly discuss this in the manuscript to justify why this more complex learning approach was chosen over a simpler label-based graph construction method.
4. Description of the Inference Process for New Molecules: This is a crucial point for practical application. The graph built during training includes all training molecules. However, during inference for a novel molecule not in the training graph, how does the model calculate its fused feature $\tilde{x}$_new? The "Methods" section (4.3) does not explicitly describe the inference mechanics. This needs to be detailed in the main text or supplementary materials. Clarifying this is essential for the paper's rigor and reproducibility.
5. Comprehensiveness of Baseline Model Selection: The comparison against classic ML models (SVR, MLP, CatBoost), a CNN, and a Vanilla Transformer is good. However, in the field of molecular prediction, Graph Neural Networks (GNNs), particularly those that directly process molecular structural graphs (e.g., built from SMILES or via RDKit atom-bond graphs) such as MPNN, DMPNN, or Attentive FP, are highly relevant and powerful baselines. The lack of comparison against such SOTA GNN baselines is a gap. I strongly recommend adding this comparison or, at a minimum, discussing the rationale behind the current baseline selection and comparing the advantages/disadvantages of the proposed approach versus standard GNNs.

(Remarks on code availability)

Version 1:

Reviewer comments:

Reviewer #2

(Remarks to the Author)

(Remarks on code availability)

Reviewer #3

(Remarks to the Author)
The author answered the reviewer's questions and made revisions to the article. I think this article can be accepted now.

(Remarks on code availability)

Open Access This Peer Review File is licensed under a Creative Commons Attribution 4.0 International License, which permits use, sharing, adaptation, distribution and reproduction in any medium or format, as long as you give appropriate credit to the original author(s) and the source, provide a link to the Creative Commons license, and indicate if changes were made.
In cases where reviewers are anonymous, credit should be given to 'Anonymous Referee' and the source.
The images or other third party material in this Peer Review File are included in the article’s Creative Commons license, unless indicated otherwise in a credit line to the material. If material is not included in the article’s Creative Commons license and your intended use is not permitted by statutory regulation or exceeds the permitted use, you will need to obtain permission directly from the copyright holder.
To view a copy of this license, visit https://creativecommons.org/licenses/by/4.0/

Ms. Ref. No.: NCOMMS-25-56305
Title: LG-Transformer: Learned-Graph Transformer Framework Enabling Diverse Physicochemical Property Prediction toward Fuel Design

Response to the reviewers:
We thank the reviewers for their constructive comments to improve the quality of the manuscript. Our responses to the reviewers' comments are marked in blue color.

Reviewer #1:

General Comments: The manuscript introduces LG-Transformer, a hybrid framework that (i) learns a property-aware graph via contrastive learning on descriptor embeddings, (ii) propagates features with a GCN, and (iii) models inter-descriptor dependencies with a Transformer encoder to predict 17 physicochemical properties for 1,850 molecules spanning 26 classes. The authors assemble a new database and report an average $R^2 \approx 0.933$ with interpretability analyses based on integrated gradients (IG). The problem is relevant, the dataset curation is substantial, and the architecture is well-motivated. However, several major issues around problem framing (multi-objective vs. single-task), validation scheme, evaluation design, dataset curation should be addressed.

1. It is unclear what the authors refer to by "multi-objective." If this means utilizing a unified backbone (i.e., shared parameters) with task-specific heads (i.e., multi-task learning, MTL), then it is important to quantify the advantage of MTL relative to single task learning through ablations. It is also important to adopt the naming convention used within ML and molecular property prediction domains (multi-task learning) to avoid confusing the readers.

In our work, the term "multi-objective" does not refer to MTL with shared parameters and task-specific heads. Instead, our framework is designed as a unified architecture that can be applied independently to predict different physicochemical properties, i.e. the same backbone can be adapted to diverse prediction tasks. Our goal is to demonstrate the generalizability of LG-Transformer across diverse fuel-related properties, rather than to optimize them jointly within an MTL setting.

To avoid confusion, we have revised the manuscript to remove the term "multi-objective" and other potentially ambiguous expressions. The updated wording clearly reflects the single-task nature of the proposed model throughout the **Title**, **Abstract**, **Introduction**, **Methods**, and **Results** sections.

2. Tab. 1 shows many NA entries and highly uneven counts across classes and properties (e.g., bicycloalkanes, terpenes). This shows that the collected dataset has problems with label missingness or imbalance. The imbalance also exists where some large families (e.g., aromatics, alkenes) dominate counts versus sparse families. Do these risks bias training and unstable estimates for low-data cells?

The observed missingness and imbalance reflect the characteristics of the fuel domain, where some fuel classes, such as aromatics and alkenes, are widely used in engines and therefore have extensive measurements, while other classes, such as bicycloalkanes and terpenes, are much less common and contain fewer data points. In building the database, we have attempted to maximize coverage, and the current collection of 26 chemical families and 17 physicochemical properties represents, to our knowledge, the most comprehensive collection for engine-relevant fuels. The detailed description of the dataset has been added in **"Results: Dataset construction**

for predicting diverse fuel properties" on Page 7 Lines 167–173.

As highlighted in Q1, LG-Transformer is trained separately for each property, so missing labels in one property do not affect learning in others. To further assess the effect of data imbalance, detailed performance comparisons across six key fuel property datasets (EOV, viscosity, ST, RON, MON, and DCN) are presented in Tab. R1. The results demonstrate that LG-Transformer achieves highly competitive performance across all fuel categories, including those with limited samples such as Ac-X (Count = 6), where it attains an R^2 of 0.960, indicating robust predictive accuracy even in data-sparse regimes. Moreover, the framework consistently maintains high R^2 values across the majority of fuel groups (e.g., HC, CHC, AROM, ALET, ALKE, PolyF, and EPOX) compared with conventional GNN and deep learning baselines. This robustness arises from the graph-based representation learning in LG-Transformer, which captures shared structural patterns across diverse fuel categories. As a result, the model generalizes effectively despite uneven data distributions, highlighting its strong adaptability for real-world fuel property prediction. The corresponding discussion is provided in **"Results: Performance comparison between LG-Transformer and other baseline models on fuel property prediction"** (Pages 14–15 Lines 264–276) of the main text, and Tab. R1 has been updated as **Tab. 4**.

Table R1 The performance comparisons of different models among six key fuel property datasets (EOV, Viscosity, ST, RON, MON, and DCN) for different fuel groups, reported from a representative split of the five-fold cross-validation.

**Fuel group**	**Count**	**Mean R^2**				
		**LG-Transformer**	Transformer [29]	CatBoost [24]	MolCLR [90]	AttentiveFP [85]
**HC**	164	**0.982**	0.960	0.972	0.967	0.974
**CHC**	77	**0.955**	0.937	0.942	0.943	0.945
**AROM**	63	**0.992**	0.976	0.973	0.982	0.981
**ALET**	57	**0.966**	0.958	0.950	0.957	0.954
**ES**	36	**0.976**	0.965	0.960	0.968	0.972
**ALKE**	25	**0.984**	0.973	0.974	0.969	0.974
**PolyF**	18	**0.966**	0.945	0.956	0.945	0.965
**EPOX**	8	**0.996**	0.985	0.992	0.990	0.992
**Ac-X**	6	**0.960**	0.923	0.933	0.955	0.947

3. Lack of comparison with comparable and state-of-the-art (SOTA) deep learning models for molecular property prediction. While the authors follow a mostly standard procedure for baselining, they compare only to classical techniques (boosting, SVR), and non-graph based methods (descriptors-based MLP implementation and a generic transformer). A fairer comparison should include models established for molecular property prediction applications on multiple properties, including ones based on molecular graphs. Examples include D-MPNN/Chemprop [1], ML-QSPR [2] , Attentive FP [3], and ACS [4]. Also important to gauge performance against SOTA contrastive learning models, such as [5], [6]and others.

To ensure a fair and comprehensive assessment, additional experiments have been conducted comparing LG-Transformer with a set of recently proposed state-of-the-art graph-based and contrastive learning baselines, including Chemprop [89], ML-QSPR [88], AttentiveFP [85], ACS [84], MolCLR [90], and KANO [91], using the same multi-property fuel dataset. To ensure statistical reliability and mitigate dataset bias, the original data split has been replaced with a rigorous five-fold cross-validation protocol (see more details in Q5). All models, including newly added baselines, are retrained and evaluated in this unified setting.

The updated results reported in Tab. R2 show that LG-Transformer consistently outperforms these SOTA methods across the majority of fuel properties, achieving the highest average R^2 of 0.900 and demonstrating robust generalization. Graph-based methods, such as Chemprop [89] and AttentiveFP [85], as well as contrastive models including MolCLR [90] and KANO [91], primarily rely on structural representations. However, these models fail to adequately capture the nuanced relationship between molecular similarity and property variation, resulting in less stable predictions for complex properties, such as RON, MON, and DCN. In contrast, the feature fusion strategy of LG-Transformer effectively handles the complex relationship between molecular structure and physicochemical characteristics, thereby enabling more reliable cross-property predictions.

In the manuscript, the rationale for selecting the above SOTA methods has been detailed in **"Results: Performance comparison between LG-Transformer and other baseline models on fuel property prediction"** (see Page 11 Lines 215–224) and **"Methods: Baselines"** (see Page 30 Lines 570–573) of the main text. The comparative results (Tab. R2) have been incorporated into **Tab. 3** (excluding ACS due to its consistently poor performance), and the corresponding analysis text has been expanded on Pages 11–14 Line 238–252. Furthermore, **Fig. 3** has been updated to illustrate the underlying reasons for the superior performance of LG-Transformer, using AttentiveFP and MolCLR as representative examples to explain its advantages across different physicochemical property predictions. This updated evaluation reinforces the conclusion that the proposed architecture provides a substantial advancement in molecular property prediction for fuel-related applications.

Table R2 Performance comparison of the benchmark models (R^2 coefficients). The table evaluates predictive accuracy for key fuel physicochemical properties across our model and 6 cutting-edge models. Results are reported as the average test R^2 scores from 5-fold cross-validation. Our proposed LG-Transformer consistently achieves the best performance across all properties.

	**Properties**	ML-QSPR[88]	Chemprop[89]	AttentiveFP[85]	MolCLR[90]	KANO[91]	ACS[84]	LG-Transformer
**Physical Properties**	**Viscosity**	0.835±0.033	0.859±0.033	0.869±0.043	0.858±0.044	0.875±0.029	-1.874±1.622	**0.894±0.024**
	**EOV**	0.965±0.009	0.952±0.018	0.967±0.025	0.944±0.027	0.952±0.020	0.569±0.363	**0.979±0.003**
	**SEF**	0.909±0.052	0.922±0.056	0.929±0.050	0.925±0.049	0.928±0.051	0.365±0.347	**0.940±0.044**
	**VP**	0.837±0.067	0.891±0.033	0.912±0.054	0.883±0.032	0.882±0.040	-0.048±1.266	**0.928±0.070**
	**ST**	0.732±0.088	0.780±0.096	0.799±0.132	0.817±0.125	0.790±0.132	-0.190±0.297	**0.835±0.133**
	**FP**	0.919±0.030	0.911±0.030	0.927±0.022	0.928±0.022	0.910±0.021	0.437±0.443	**0.931±0.020**
	**Density**	0.867±0.012	0.930±0.012	0.934±0.016	0.929±0.019	0.919±0.019	-186.519±283.345	**0.947±0.017**
	**BP**	0.802±0.027	0.812±0.023	0.839±0.012	0.814±0.026	0.814±0.026	0.748±0.030	**0.852±0.030**
	**MP**	0.748±0.029	0.757±0.030	0.772±0.029	0.757±0.020	0.772±0.025	-0.616±1.015	**0.796±0.044**
**Chemical Properties**	**RON**	0.779±0.034	0.794±0.041	0.786±0.057	0.805±0.050	0.822±0.040	-0.858±0.564	**0.856±0.038**
	**MON**	0.731±0.032	0.780±0.026	0.778±0.028	0.761±0.035	0.770±0.024	-4.754±3.905	**0.807±0.042**
	**CN**	0.758±0.056	0.795±0.048	0.782±0.067	0.798±0.032	0.806±0.047	-0.439±0.184	**0.843±0.047**
	**DCN**	0.654±0.117	0.804±0.085	0.826±0.073	0.768±0.072	0.830±0.076	-1.146±1.046	**0.842±0.066**
	**LHV**	0.993±0.003	0.989±0.006	0.994±0.001	0.991±0.002	0.993±0.003	0.987±0.008	**0.995±0.001**
	**UFL**	0.850±0.014	0.864±0.018	0.887±0.031	0.871±0.027	0.873±0.047	-2.198±5.718	**0.917±0.013**
	**LFL**	0.788±0.075	0.849±0.042	0.914±0.054	0.860±0.036	0.896±0.032	-117.967±158.914	**0.953±0.013**
	**YSI**	0.956±0.020	0.963±0.013	0.973±0.014	0.946±0.006	0.968±0.009	0.655±0.121	**0.982±0.005**
**MEAN**		0.831±0.041	0.862±0.036	0.876±0.042	0.862±0.037	0.871±0.038	-18.403±27.011	**0.900±0.036**

4. Beyond the authors' curated dataset, evaluation on standard molecular property datasets (e.g., MoleculeNet [7]) would provide a better basis for comparison and position this work relative to existing literature.

As pointed out by the reviewer, to evaluate the generalization capability of LG-Transformer beyond the domain of fuel property prediction, experiments have been conducted on five regression tasks from the widely used MoleculeNet benchmark [96], namely ESOL, FreeSolv, Lipophilicity, QM7, and QM8. These tasks are selected as they closely align with the nature of the molecular property prediction problem addressed in this study, covering both physicochemical and quantum-mechanical properties. Tab. R3 summarizes the performance of all compared models on the standard MoleculeNet benchmark tasks, reporting test errors in terms of RMSE or MAE for each dataset under identical train/validation/test splits. LG-Transformer is evaluated alongside several SOTA methods, including KANO, MolCLR, D-MPNN, and other representative graph-based approaches [90–91, 97–105]. The results clearly demonstrate that the proposed LG-Transformer architecture not only excels in fuel-specific applications but also generalizes effectively to diverse molecular regression tasks, reinforcing its competitiveness in the broader context of molecular representation learning.

Detailed discussion has been added to the **"Discussion"** (Pages 22–23 Lines 389–410) to describe the generalization capability of LG-Transformer to a wider range of molecular regression tasks within the MoleculeNet benchmarks and for predicting properties of unseen fuel molecules (see more details in Q2 from Reviewer #2).

Table R3 Test performance of different models on six regression benchmarks of physical chemistry and quantum mechanics in the MoleculeNet. LG-Transformer achieves the best performance on most tasks. Reported are the mean and standard deviation of RMSE (for ESOL, FreeSolv, Lipophilicity) or MAE (for QM7, QM8) over three independent runs.

**Model**	**Physical Chemistry (RMSE)**			**Quantum Mechanics (MAE)**	
	**ESOL**	**FreeSolv**	**Lipophilicity**	**QM7**	**QM8**
Dataset size	1,128	642	4,200	7,160	21,786
Number of tasks	1	1	1	1	12
GCN [97]	1.431±0.050	2.870±0.135	0.712±0.049	122.9±2.2	0.0366±0.0000
GIN [98]	1.452±0.020	2.765±0.180	0.850±0.071	124.8±0.7	0.0371±0.001
MPNN [99]	1.167±0.430	1.621±0.952	0.672±0.051	111.4±0.9	0.0148±0.001
DMPNN [100]	1.050±0.008	1.673±0.082	0.683±0.016	103.5±8.6	0.0156±0.001
CMPNN [101]	0.798±0.112	1.570±0.442	0.614±0.029	75.1±3.1	0.0153±0.002
N -GRAM [102]	1.100±0.030	2.510±0.191	0.880±0.121	125.6±1.5	0.0320±0.003
Hu et al. [103]	1.100±0.006	2.764±0.002	0.739±0.003	113.2±0.6	0.0215±0.001
GEM [104]	0.813±0.028	1.748±0.114	0.674±0.022	60.0±2.7	0.0163±0.001
GROVER [105]	1.423±0.288	2.947±0.615	0.823±0.010	91.3±1.9	0.0182±0.001
MolCLR [90]	1.113±0.023	2.301±0.247	0.789±0.009	90.9±1.7	0.0185±0.013
$\text{MolCLR}_{\text{CMPNN}}$ [90]	0.911±0.082	2.021±0.133	0.875±0.003	89.8±6.3	0.0179±0.001
KANO [91]	0.797±0.098	1.670±0.076	0.68±0.035	75.2±2.8	0.0147±0.0012
**LG-Transformer**	**0.5859±0.022**	**0.9531±0.195**	**0.587±0.024**	**53.2±3.4**	**0.010255±0.0004**

5. To verify the generalizability of the model, it is paramount to evaluate its performance on k-fold cross-validation training split or some of its variations. The traditional train/test split is known to overestimate model performance, especially with the lack of an unseen validation set which allows overfitting on the test data. Moreover, the hyperparameter optimization process and corresponding parameter search space are not clearly described in the manuscript. To ensure fair performance reporting, the authors should evaluate the model performance on k-fold cross-validation or a Murcko-scaffold split [8]. This also serves to demonstrate the stability of the training technique and robustness to random parameter initializations.

We agree with the reviewer that a traditional random train/validation/test split may overestimate performance in certain cases. To provide a more rigorous evaluation of model generalizability, stability, and robustness, the experimental pipeline has been restructured. Specifically, the previous single split has been replaced with a five-fold cross-validation scheme. This procedure has been applied consistently to LG Transformer as well as the newly added SOTA baseline methods. Details about cross-validation have been clarified on Page 7 Lines 184–186 (**"Results: Framework of proposed LG-Transformer"**) and Page 11 Lines 226–231 (**"Results: Performance comparison between LG-Transformer and other baseline models on fuel property prediction"**) of the main text, and the model performance in Tab. 3 and 5 are revised.

Moreover, the search space and experiment settings for key hyperparameters during training is detailed in **"Methods: Model training"** (Page 29 Lines 537–542) and the Supplementary Materials (**Supplementary Tabs. 4-7**), and validation performance is monitored with early stopping to avoid overfitting. The revised results (see the updated **Tab. 3 and Fig. 3**) confirm that LG Transformer maintains the highest predictive accuracy among all baseline models. These results demonstrate that the model provides stable and unbiased predictions across structurally diverse molecules, while preventing data leakage between hyperparameter tuning and evaluation stages.

6. Lack of detailed information about training dataset curation. The data sources provided on https://ai4fuel.sjtu.edu.cn/ show datasets known to contain predicted property values (e.g., DIPPR 801). It is important to train the models solely on experimental and rigorously derived fuel property values. A table of units and temperatures for every property, and how data drawn from multiple sources was standardized is necessary. In the supplementary data, it is important to include the data source of each fuel property value. Moreover, to facilitate results reproduction, please include a script in the code repository to enable quick training and result validation.

Any predicted or estimated values reported in the literature (e.g., DIPPR 801) have been strictly excluded to ensure that all retained data are derived solely from experimental measurements. To avoid misunderstandings, the source of each experimental value has been clearly annotated in the open-source dataset on Figshare (`https://figshare.com/articles/dataset/fuel_database_for_LG-Transformer/29571449`). Moreover, the website `https://ai4fuel.sjtu.edu.cn/` is updated for better access.

To further illustrate the standardization process, Tab. R4 provides the units, test standards and methods, measurement uncertainties, and other relevant information for each property. Specifically, EOV, SEF, VP, and ST are measured at 25 °C, where EOV and SEF use calorimetry, VP adopts the Reid method, and ST uses the ring method. Viscosity is measured at 20 °C following ASTM D445, FP uses the Pensky–Martens closed-cup test, density is determined via digital densimetry, MP uses the capillary tube method, LHV employs bomb calorimetry, and YSI applies color-ratio pyrometry. RON, MON, CN, DCN, BP, UFL, and LFL are all obtained

in accordance with ASTM standards. Note that, when multiple sources report values for the same fuel property, measurements conducted under ASTM or equivalent standardized conditions are preferentially selected. These unified measurement protocols ensure consistent and high-quality data across properties.

To address this comment, additional details have been incorporated throughout the manuscript. Specifically, Tab. R4 has been added to the main text (**"Results: Dataset construction for predicting diverse fuel properties"**, Page 5). The description of physical and chemical properties in (**"Results: Dataset construction for predicting diverse fuel properties"** (Page 6 Lines 137–145 154–160 and Page 7 Lines 167–173) has been expanded accordingly to explain these database characteristics in detail. Furthermore, **"Methods: Data preparation"** (Page 28 Lines 514–524) now elaborates on the database construction workflow, including data cleaning, screening, and validation procedures to ensure data reliability (see more details in Q9 and Q10). Moreover, a corresponding script has been included in the code repository to enable users to perform rapid validation and consistency checks.

Table R4 Units, measurement standards, data counts, and uncertainties for physical and chemical properties. All data are collected from standardized experiments to ensure reliability and consistency.

	Properties	Units	Test methods or standards	Train data	Test data	Uncertainties
Physical Properties	**Viscosity**	mPa · s	ASTM D445 [55]	253	63	± 0.35 % [68]
	**EOV**	kJ mol^{-1}	Calorimetry (25 °C) [55]	442	110	± 0.5 kJ/mol [69]
	**SEF**	kJ mol^{-1}	Calorimetry (25 °C) [55]	269	67	± 5.0 kJ/mol [23]
	**VP**	kPa	Reid Method (25 °C) [55]	555	139	± 5.5 kPa [70]
	**ST**	dyne/cm	Ring Method (25 °C) [63]	416	104	± 0.9 dyne/cm [71]
	**FP**	°C	ASTM D93 [55]	843	211	± 5 °C [72]
	**Density**	g cm^{-3}	Densimetry (15-30 °C) [55]	950	237	± 0.0005 g cm^{-3} [73]
	**BP**	°C	ASTM D86 [55]	1007	252	± 2.0 °C [74]
	**MP**	°C	Capillary Tube Method [55]	854	214	± 0.5 °C [75]
Chemical Properties	**RON**	–	ASTM D2699 [76]	307	77	± 1.0 [76]
	**MON**	–	ASTM D2700 [77]	277	69	± 1.0 [77]
	**CN**	–	ASTM D613 [78]	510	128	± 2.5 [78]
	**DCN**	–	ASTM D6890 [53]	142	36	± 2.0 [53]
	**LHV**	kJ mol^{-1}	Bomb Calorimetry [58]	839	210	± 0.2 % [79]
	**UFL**	%	ASTM E681, E682 [58]	462	115	±0.5 vol% [58]
	**LFL**	%	ASTM E681, E682 [58]	650	163	±0.2 vol% [58]
	**YSI**	–	Color-Ratio Pyrometry [54]	354	89	MAX(±4.0, 3.0%) [54]

7. Units of Mean Absolute Error values are missing in Table 2. The readers cannot discern the significance of reported MAEs if the physical unit is missing (e.g.; 1.3 Pa v.s. 1.3 kPa for VP in Table 2). Moreover, aggregation of MAEs across properties with different units (i.e., MAE 13.12 in Table 2) is not meaningful and can misguide the reader into thinking it can be used as a universal metric to compare across models. Average R^2 is a better choice.

The units of the MAE values are consistent with those listed in Tab. R4. However, to improve clarity and address the reviewer's concern regarding aggregation across properties with different units, all MAE values have been removed from **Tab. 3** (Page 12) in the revised manuscript. Model performance is now consistently reported using the R^2 metric, which provides a unit-independent and directly comparable measure across different properties.

8. Splitting is performed per property, implying varying sample sets per target; cross-property correlations may be underexploited and evaluation comparability across targets can be tricky. Do authors consider this in contrastive learning?

As clarified in the response to Q1, the modeling approach is fundamentally distinct from MTL, as no parameters or learning signals are shared across properties. The strategy to train models independently for each property reflects the intrinsic nature of the fuel properties. The 17 properties considered are fundamentally distinct and largely uncorrelated. For example, properties representing physical characteristics, such as density, are independent of chemical characteristics, such as knock resistance (RON). Consequently, each property is selected as an independent training target, which also ensures that contrastive learning focuses on meaningful similarities between molecules for the same property, thereby maintaining accuracy and effectiveness in the learning process. This discussion has been added on Page 7 Lines 177–181 of **"Results: Framework of proposed LG-Transformer"** for clarity.

9. The dataset is collected from heterogeneous sources, so it's important to spell out the specific property units, detailed unit harmonization, conflict resolution, quality control, and outlier handling procedures in the main text or caption of Fig. 1.

The requested information has been added to the revised manuscript. As highlighted in Q6, **"Results: Dataset construction for predicting diverse fuel properties"** now elaborates on the unit harmonization, standard testing conditions, and uncertainty ranges for each property by adding **Tab. 1** (Page 5 Lines 123–127).

On the other hand, **"Methods: Data preparation"** has been expanded to describe the full data processing workflow, including conflict resolution, data cleaning, quality control, and outlier handling (Page 28 Lines 514–524). Specifically, conflicting entries are resolved by prioritizing values from recognized standards such as ASTM, followed by peer-reviewed experimental data (see Tab. R4). Outliers are identified through combined statistical and structure-based checks. These systematic procedures, along with duplicate screening and manual verification of abnormal entries, ensure that the final dataset used for model training is accurate, reliable, and reproducible.

10. Are measurement uncertainties recorded or retrievable from the cited sources? Authors should estimate and report the uncertainty for each property in the dataset to enable usage of the curated dataset.

Most measurement uncertainties can be directly obtained from the original databases or corresponding standard testing methods. Specifically, uncertainties for RON, MON, CN, DCN, SEF and YSI are recorded in the cited literature or experimental reports, while those for FP, density, BP, LHV, UFL, and LFL are derived from standardized test methods following ASTM protocols. These standardized sources ensure the traceability and reliability of uncertainty information.

For properties such as EOV, VP, ST, and MP, the majority of fuels have reported measurement

uncertainties in authoritative databases. These available data were systematically reviewed and summarized to determine representative uncertainty ranges for the overall dataset. Specifically, EOV uncertainties are derived from calorimetric data in NIST Chemistry WebBook, VP follows the Reid vapor pressure method, ST follows the ring method, and MP follows the capillary tube method. The summarized uncertainty ranges and their corresponding references are presented in **Tab. 1** (Page 5), with detailed explanations provided in **"Results: Dataset construction for predicting diverse fuel properties"** and **"Methods: Data preparation"** of the revised manuscript.

11. In contrastive learning, negatives are defined as label-dissimilar yet feature-close samples. Early in training most pairs may be far in embedding space; how are sufficient negatives found or updated across epochs? I think more detail on the mining schedule would help.

In the early stages of training, the empirical distribution of pairwise distances is examined, and the marginal value τ is set to a stable value to ensure that sufficient negative samples could be reliably found. If there are no negative samples left during the training process, then there is no need for further training. This design collectively guarantees that effective negative samples are available throughout training while maintaining a consistent contrastive objective. The training results of the embedding network with different marginal value τ are discussed in **"Results: The effectiveness of learned-graph feature fusion"** (see Pages 16–17 Lines 301–306), with detailed results provided in Supplementary Tab. 2 of the supplementary materials.

12. In Methods, Norm(G) is introduced but not defined (row-norm? sym-norm? softmax over neighbors?). This choice would affect stability and scaling; please detail it.

$\mathrm{Norm}(G)$ refers to row-wise normalization of the adjacency matrix, where each row is divided by the sum of its elements so that all outgoing edge weights for a node sum to 1. This operation ensures that the aggregated neighbor information remains on a consistent scale across nodes, regardless of their degree or the number of neighbors. Such normalization stabilizes the message propagation process, prevents nodes with many connections from dominating the aggregation, and improves numerical stability during graph-based feature fusion. The corresponding modification has been added on Page 26 Lines 479–480 of the main text (**"Methods: GCN Feature propagation based on embedding"**).

13. GCN propagation depth/hops: the authors seem to use a single aggregation with $\tilde{A}$ in eq. (9). Did the authors try deeper propagation (e.g., stacked GCN layers) and observe over-smoothing or gains? Please clarify the design choice.

This framework intentionally adopts a single-layer GCN propagation using $\tilde{A}$, as finding that deeper GCN structures (e.g., stacked two-layer propagation) do not lead to performance gains but rather introduce instability and noise. This observation is consistent with prior work [106], which reported that multi-layer GCNs tend to accumulate noise from uncertain or sparsely connected graphs, leading to degraded representations. Therefore, a single aggregation layer is selected for both empirical effectiveness and computational efficiency, avoiding potential over-smoothing and ensuring stable message propagation. The corresponding modification has been added to Page 26 Line 486–488 (**"Methods: GCN Feature propagation based on embedding"**) of the main text.

14. About the descriptor-token formation details, when building the ks$\times$ kd token grid: how are missing descriptors handled, are groups padded/masked, and is token order fixed across

molecules? Mordred can yield NaNs. The paper does not state whether NaNs are imputed (mean/median/zero), dropped, or handled via a learned "missing" mask/channel. Standardization alone is insufficient context here because it presupposes finite values. This should be discussed. In addition, descriptor groups differ in size, yet the Transformer requires a fixed per-token width kd. The authors should describe which strategies are used here (e.g. padding, masking, pooling, or projection).

In the implementation, descriptors computed by Mordred occasionally yield missing or NaN values. All such missing entries are imputed with zero prior to standardization, ensuring that each molecular descriptor vector has a consistent dimension and remains numerically stable during scaling. When constructing the descriptor-token grid of size $k_s \times k_d$, each descriptor group (corresponding to one token) is assigned a fixed order according to the group mapping rules defined by the Mordred descriptor group. For groups containing fewer than k_d descriptors, the remaining positions are padded with zeros to maintain a uniform per-token width required by the Transformer encoder. This design keeps the token order consistent across all molecules while avoiding additional learned mask channels or imputation uncertainty. The corresponding modification has been added to Page 27 Lines 494–495 (**"Methods: Encoder-based transformer architecture"**) of the main text.

15. About the baselines, the paper compares the LG-transformer against traditional ML (SVR, MLP, CatBoost), a descriptor-only Transformer, and CNN, however, authors should also include some modern, strong molecular baselines, such as D-MPNN, Graphormer, MolFormer, ChemBERTa, etc. As written, the baseline roster is not state-of-the-art for molecular ML, which undercuts the "SOTA"claim.

In alignment with the response to Q3, the comparative analysis has been expanded to incorporate several modern and strong molecular baselines, including Chemprop [89], ML-QSPR [88], AttentiveFP [85], ACS [84], MolCLR [90], and KANO [91]. The results from these additional baselines are included in **Tab. 3** (Page 12), thereby providing a more comprehensive and up-to-date benchmark. These comparisons reinforce the robustness of the evaluation and confirm the superior performance of LG-Transformer within the current landscape of molecular property prediction methods.

16. Only random class-stratified splits are used. Since the dataset includes a range of fuel families, it is suggested that scaffold and leave-class-out splits should be reported. Random splits often overestimate performance. Authors should consider these splits enforcing structural dissimilarity or extrapolation across fuels to mirror real deployment of fuel design.

As clarified in our response to Q2, all experiments employ five-fold cross-validation to prevent data leakage and to ensure statistically reliable performance estimates. Because the fuel categories relevant to practical applications are comprehensively covered in Tab. 2, the probability of encountering structurally or categorically unfamiliar fuels is minimal. Consequently, scaffold or leave-class-out splits are not used. Instead, to more accurately assess real-world generalization, the model is evaluated on an external dataset that contains no molecular overlap with the training data. The corresponding results are reported in Tab. 6 (Page 22).

17. In ablation studies, because the graph is learned with label-defined positives, the authors should explicitly explain split-safe construction in detail to prevent leakage influencing ablation results. For example, in the "-contrastive" setting, replace the learned embedding with a label-free alternative and avoid building any graph from label distances; that would be direct leakage.

It is confirmed that all contrastive learning experiments are conducted under a strict five-fold cross-validation protocol, where the training, validation, and test sets are completely disjoint. The contrastive embedding is learned solely from the training set of each fold, ensuring split-safe construction. It is worth noting that during the validation/testing phase, only the descriptors of the molecules are used for the sample connection operation, and no label information is utilized, thus preventing label leakage. The specific prediction process has been elaborated in a new section (**"Methods: Model Inference"**, Pages 29–30 Lines 543–559) of the text.

Moreover, in the "-contrastive" ablation setting, the contrastive module is entirely removed. The model directly uses the descriptor-based features without any learning process. Therefore, this configuration contains no risk of data leakage.

18. The authors mention MSE and MSLE loss functions. Please clarify which tasks use which loss and why, and whether losses were task-specific in a multi-task setting.

The selection of loss functions for different task is depended on the performance of validation set, which is like the selection of hyperparameters. To standardize the training process and enhance efficiency, logarithmic transformation is applied to fuel properties with high value variance in the revised version, thereby normalizing their scales. This allowed us to unify the training process under the MSE loss, rendering the use of the MSLE loss unnecessary. Besides, in our experimental setup, a single-task learning paradigm is adopted instead of MTL, where a separate model is trained for each fuel property prediction task. The corresponding modifications have been revised in **"Methods: Encoder-based transformer architecture"** (Page 28 Lines 509–511) and **"Methods: Model training"** (Page 29 Lines 535–536) of the manuscript.

Reviewer #2:

General Comments: This manuscript describes a new machine learning approach for the prediction of mono fuel properties (physical and chemical) for the evaluation of new fuels. The novel approach is a two-stage approach to representation learning to improve the prediction accuracy for multiple properties using the same architecture. The manuscript is well-written and well structured. The manuscript well describes the method used and the logic behind the approach. The authors also describe well the training and optimization procedure for the neural network and validation method of the LG-transformer. I see that the authors have used an optimizer algorithm to optimize their neural network – if it has not been done it always sound to read up on "rules of thumbs" on ranges for the optimization parameters depending on the spread of data and number of data points used in the training data sets, and see if these are aligned with those of results of the optimization algorithm. What could improve the validity of the LG-transformer is:

1. A list of number of data points used for training and validation for each individual property. I know from experience that some property data is easier to find or measure than other. It should be transparent how much training data is available for the neural network.

The number of data points designated for training and testing for each individual property has been provided and is detailed in Tab. R4. More details are shown in the response to Q6 from # Reviewer 1. This information has also been provided on **Page 5** of the main text.

2. Adding a final validation after the model is defined with random datapoints from different property datasets that was not incorporated in the initial training and validation. This can help spotting any optimization issues causing the model to be less general for future predictions on untrained input.

An independent external dataset has been assembled from two additional sources [24,80], including 88 RON, 37 MON, 48 EOV, 169 density, and 172 BP data points, including 260 previously unseen fuel molecules. This dataset is entirely separate from the training and validation data and is used exclusively for final model assessment. The graph learning approach for predicting unseen fuel properties is described in Q4 from Reviewer #3. The results, as summarized in Tab. R5, confirm the model robustness and strong predictive performance across new, untrained data.

Table R5 Comparison of R^2 scores for LG-Transformer and several baseline models on an unseen test dataset.

**Dataset**	**LG-Transformer**	Catboost [24]	Transformer [29]	MolCLR [90]	AttentiveFP [85]
**RON**	**0.886±0.038**	0.835±0.049	0.825±0.032	0.857±0.042	0.866±0.026
**MON**	**0.830±0.067**	0.763±0.079	0.787±0.058	0.817±0.064	0.789±0.069
**EOV**	**0.956±0.013**	0.949±0.029	0.940±0.038	0.949±0.018	0.944±0.025
**BP**	**0.831±0.049**	0.770±0.048	0.808±0.038	0.789±0.049	0.824±0.058
**Density**	**0.954±0.006**	0.914±0.016	0.887±0.009	0.934±0.013	0.940±0.006
**MEAN**	**0.892±0.035**	0.846±0.044	0.849±0.035	0.869±0.037	0.873±0.037

Detailed discussion is added to the **"Discussion"** (Pages 22–23 Lines 389–409) to detail the prediction of unseen fuel properties and to discuss the broader applicability of the proposed LG-Transformer method on Page. 23 Lines 418–419 (see also Q4).

3. One important thing is that the model can capture the behavior in the predictions. For example, it is briefly discussed in the Discussion section that the alkanes show lower octane number and higher cetane number, which is expected, but how did the model capture the behavior of other molecules? Or how did it behave when multiple functional groups where present in one molecule? On the same subject it is also interesting to discuss if there where ranges or molecules where the model performed worse and if so if these are ranges/molecules important for fuel evaluation or not. This can be done with showing the full predictions (predicted value vs measured value) in a few graphs also showing important outliers (if any).

To directly address this point, detailed discussion ((Page 20 Lines 360–380)) has been added in the revised manuscript. This examines the decision-making mechanism of the model for MON prediction through a detailed interpretability analysis based on IG score and attention mechanisms, with results shown in Fig. R1. The analysis begins with a baseline molecule (n-pentane) and compares three representative derivatives, namely 2-methylbutane (effect of structural isomerization), 1-pentanol (introduction of an oxygen-containing functional group), and prenol (presence of multiple functional groups), to investigate how the model captures the influence of different structural features. The analysis compares the baseline molecule (Fig. R1a) with three structurally modified derivatives (Figs. R1b–d), highlighting how LG-Transformer responds to molecular changes in the Mordred descriptors. Key descriptors including `MoRSE` for three-dimensional topology, `Chi` and `BCUT` for branching and electronic distribution, and `CPSA` for molecular polarity. Changes in these descriptors, caused by functionalization, branching, or multiple functional groups, are captured as positive or negative contributions by the model, which in turn manifest as the observed variations in fuel properties such as MON. This discussion is provided in **"Results: Explaining the relationship between fuel molecular structure and properties using attention-based integrated gradients method"** ((Page 20 Lines 360–380)), and Fig. R1 has been added as Fig. 6 in the main text (Page 21).

Other detailed analyses are provided elsewhere for the reviewer for complete reference. Prediction performance across different value ranges for six key fuel properties is shown in Fig. 3d of the main text, indicating strong accuracy within common ranges. Group-wise performance by fuel type is summarized in Tab. R1, showing competitive results for common categories and top performance for categories with limited data. Comprehensive predicted-versus-measured plots for all properties are provided in Supplementary Figs. 4 and 5.

4. The use of various machine learning or neural network methods to more accurately predict chemical or physical properties has been a hot topic in both chemical and mechanical engineering over the last few years. The methods are getting better as the knowledge on ML and ANNs increase over time. For fuel property prediction it is an interesting approach, but I think the significance of accurate models are higher for multi-component fuels over single-component fuels (as discussed in this paper). However, there is significance of this type of work also for other applications to improve property prediction and not only for fuel evaluation. Perhaps a wider scope than just for fuel evaluation could lead to a higher significance of this paper.

While the present work focuses on single-component fuel property prediction, the proposed LG-Transformer model is fully applicable to a wider range of molecular regression tasks. To demonstrate this broader applicability, a new discussion in **"Discussion"** (Pages 22–23 Lines 389–409)

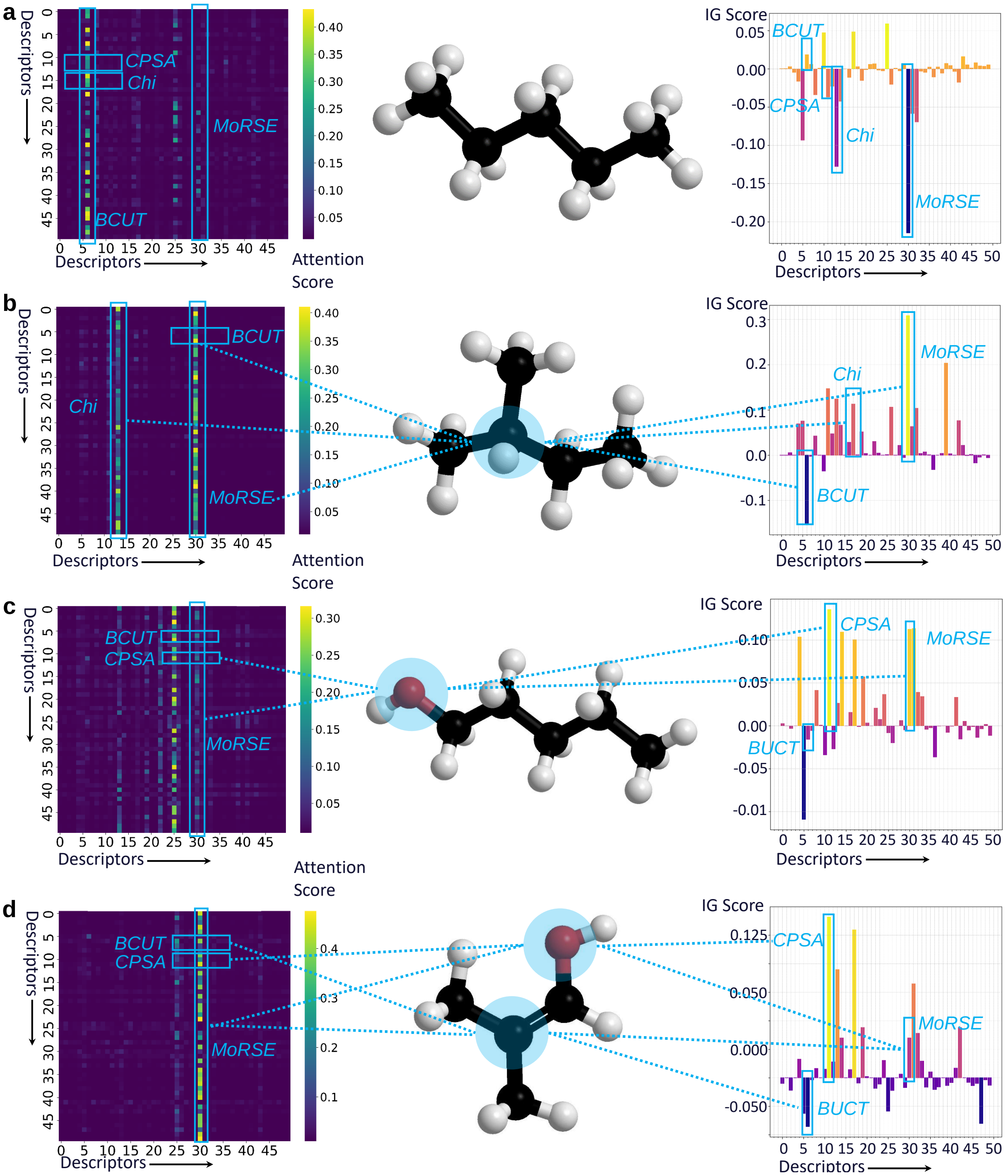

Fig. R1 Interpretability analysis of LG-Transformer for MON prediction on representative fuel molecules. The figure illustrates the decision-making process by integrating attention mechanism and Integrated Gradients analyses. **a** For the baseline molecule n-pentane, the model identifies its linear architecture as negative contributors to MON. **b** For the branched alkane 2-methylbutane, the model attributes the MON increase to its compact, spherical topology. **c** For 1-pentanol, its strong polarity and hydrogen-bonding capacity are identified as the core positive contributor. **d** For Prenol, which incorporates both branching and a functional group, this model is capable of capturing their synergistic effects.

has been added, which details the prediction of previously unseen fuel molecules using an external dataset and evaluates the model on general molecular regression tasks beyond fuel design.

As detailed in the response to Q4 from Reviewer #1, the MoleculeNet dataset is employed to evaluate the applicability of LG-Transformer beyond fuel property prediction. Five representative tasks, namely ESOL, FreeSolv, Lipophilicity, QM7, and QM8, are selected, encompassing both physical chemistry and quantum-mechanical properties. The results in Tab. R3 show that LG-Transformer achieves robust performance not only on fuel property prediction but also across these general molecular regression benchmarks. Collectively, on Pages 22–23 confirms the broad applicability of the LG-Transformer model and demonstrates the potential of the framework to advance molecular property prediction in wider chemical and engineering contexts.

5. In general the data and methodology is well described and documented. However, some elements are described in more detail than others. This cause a bit of confusion for the reader as some sections include a lot of details while other topics are only briefly discussed. The description on how the LG-transformer is used to predict different properties is unclear to me, and only mentioned briefly. The authors should better describe which properties are predicted together and if methods differ between the prediction of different properties (perhaps in a schematic?). If all properties are used for training and prediction it is important to note how the authors have dealt with properties where one node might give positive impact for one property but, for another property negative impact.

As clarified in the response to Q1 from Reviewer #1, the LG-Transformer is not applied in a multi-task learning (MTL) setup. Instead, each fuel property is predicted independently using a separate model instance, with no shared parameters across properties. This independent training ensures that no single node can exert conflicting influences across different prediction tasks. The methodology is consistent for all properties, and no properties are grouped or predicted simultaneously. For greater clarity, additional explanations and schematic illustrations of this property-specific workflow have been added to the main text (Page 7 Lines 177–181, **"Results: Framework of proposed LG-Transformer"**).

6. The presentation of the data could also benefit from a more detailed comparison of the approach described in the manuscript and the approach in other papers (e.g. comparing R^2 and root mean square error (RMSE) with the results from models in other publications (many already included in the reference list).

"Results: Performance comparison between LG-Transformer and other baseline models on fuel property prediction" on Pages 11–14 of the revised manuscript has been substantially updated to provide a more detailed comparison with prior approaches. Specifically, following Q3 from Reviewer #1, six additional state-of-the-art graph-based and contrastive learning baseline models are incorporated, including Chemprop [89], ML-QSPR [88], AttentiveFP [85], ACS [84], MolCLR [90], and KANO [91]. These comparisons, including detailed R^2 evaluations across key fuel properties, provide a more comprehensive assessment of LG-Transformer relative to existing methods.

7. I am also missing a short discussion on computational cost versus accuracy of predictions from the authors.

The computational efficiency of LG-Transformer is illustrated using the SEF dataset as an example. Fig. R2 shows the training convergence and R^2 performance of various models, including LG-Transformer, Transformer, MLP, and CatBoost. It is readily observed that LG-Transformer achieves the highest R^2 (0.97) in approximately 290 epochs, outperforming Transformer (R^2

= 0.95 at $\tilde{3}30$ epochs), MLP (R^2 = 0.93 at $\tilde{3}50$ epochs), and CatBoost (R^2 = 0.94 about 1000 epochs). This example highlights the favorable balance between predictive accuracy and computational cost for LG-Transformer. A detailed version of this comparison, including additional discussions, is provided in **"Methods: Model Training"** (Page. 29 Lines 541–542) and **Supplementary Fig. 8** of the supplementary material.

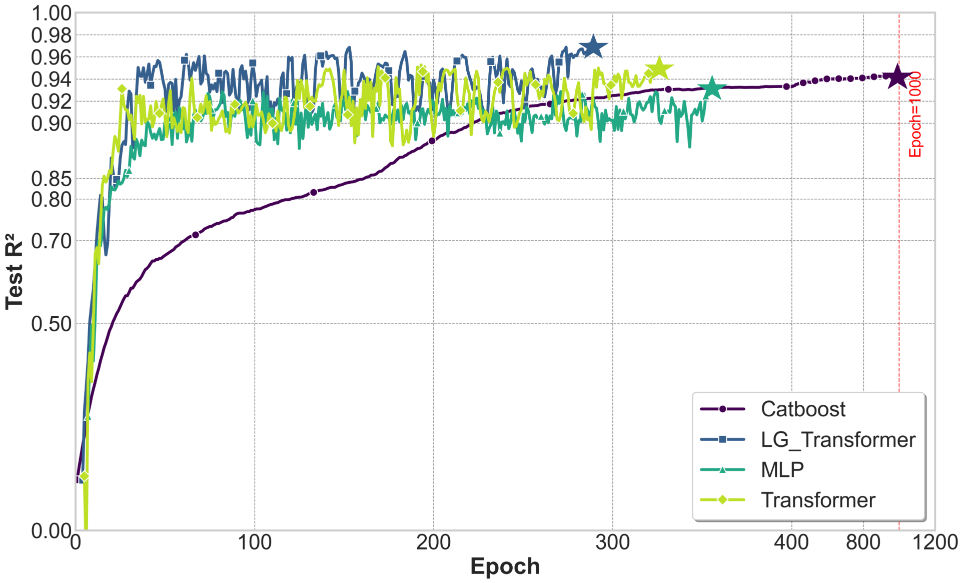

Fig. R2 Variation in computational cost and prediction accuracy for different models.

8. The authors provide enough information for the paper to be clear to the reader and for the model and methodology to be well-described. A more detailed and transparent comparison to previous work on the same topic could improve the manuscript in relation to consideration of previous work. All the necessary references are available in the reference list, the authors can use the information in those papers to compare model performances as it would better show the novelty and progress of the LG-transformer compared to previous efforts.

"Results: Performance comparison between LG-Transformer and other baseline models on fuel property prediction" on Pages 11–14 of the revised manuscript has been updated to provide a more detailed comparison with previous work on fuel design. Among the SOTA methods listed in Tab. 3, SVR, ANNs, and CatBoost have been previously applied in fuel design to predict standard enthalpy of formation, RON, MON, HOV, and laminar flame speed (LFS) [23, 24]. In light of these references, the analysis now includes additional analyses evaluating LG-Transformer across a broader set of fuel properties, with comparative visualizations provided in Fig. 3a–d. The results, summarized in Tab. 3, show that LG-Transformer consistently achieves higher R^2 values across 17 key fuel properties, demonstrating robust generalizability beyond the specific properties addressed in prior studies.

Reviewer #3:

General Comments: This manuscript presents a novel deep learning framework (LG-Transformer) for the simultaneous prediction of multiple key physicochemical properties of fuel molecules. The core contribution lies in integrating contrastive learning with Graph Convolutional Networks (GCN) and Transformer to construct a "property-aware" molecular relationship graph that enhances molecular feature representation. The authors have also compiled a high-quality dataset of 1850 molecules across 26 classes and 17 properties, which is a valuable resource in itself. The experimental design is thorough, and the results clearly demonstrate that the proposed method achieves state-of-the-art (SOTA) performance on the vast majority of prediction tasks. The paper is well-written and clearly structured. I think this manuscript is suitable for publication in this journal after proper revisions. Below are my specific comments, questions, and suggestions aimed at helping the authors further strengthen the manuscript.

1. Dataset Partitioning and Experimental Rigor: The data partitioning strategy described in the paper (Section 2.1 and Fig. 2a) involves a simple 80%/20% stratified split into a training set and a test set. All subsequent model comparisons, hyperparameter optimization (using Optuna), and model selection are based on this single split. This constitutes a major methodological flaw. Hyperparameter optimization (HPO) is inherently a form of model selection based on performance. Using the test set to guide HPO leads to data leakage, as information from the test set implicitly influences the model building process. This means the test set is no longer a truly "unseen" dataset, and the reported performance metrics (R^2, MAE) become optimistically biased estimates that do not generalize to new data.

This concern is similar to the one raised in Q5 by Reviewer #1 (see more details there). To address it rigorously, a five-fold cross-validation strategy has been implemented for all model development and hyperparameter optimization stages. In this approach, the training data are repeatedly partitioned into distinct folds, ensuring that the model is trained and validated on separate subsets without ever accessing the final test set. Consequently, hyperparameter selection relies solely on performance on the validation folds, fully isolating the test set until the final evaluation. Further details on our training results can be found in the response letter (Tab. R2) and in **Tab. 3** (Page 12) of the main text. Updates regarding the training methodology are also described on Page 11 Lines 226–231.

Furthermore, an external validation is performed using an independent dataset assembled from two sources [24,80]. To avoid data leakage, the data that are already present in the training and validation set will be removed from the new data set. The results are shown in **"Discussion"** (Page 22), and it indicates the strong performance of the model and reflects its solid generalization capability. Further specifics on this external validation are available in the response to Q2 from Reviewer #2.

2. Clarity on Novelty and Core Contribution: The paper's core innovation is the use of contrastive learning to build a property-guided molecular graph, which is then used for GCN feature propagation. This approach is ingenious and effective. However, the description of this core innovation in the "Introduction" and "Abstract" could be more prominent. It is currently somewhat generically described as "integrates contrastive learning...". I recommend more explicitly stating that unlike common GNNs based on molecular structural graphs (e.g., atom-bond graphs), the "graph" in this work is an inter-molecular relationship graph based on property similarity, which is a fundamental distinction and contribution.

The **Abstract** and **Introduction** have been revised to more clearly emphasize the core methodological contribution of this work. Specifically, the revised text (Pages 1 and 4 Lines 13–17 and 103–106) now explicitly clarifies that, unlike conventional GNNs based on molecular structural graphs, the proposed framework in this work constructs a learned graph which contains both the inter-molecular relationship and prediction similarity through contrastive learning. This key distinction is now clearly articulated to better highlight the novelty and conceptual significance of the proposed approach.

3. Justification for the Contrastive Learning Component: The ablation study shows that removing the contrastive module (-Contrastive) leads to a performance drop, proving its effectiveness. However, a critical question arises: What are the advantages of using contrastive learning to indirectly learn an embedding for graph construction, compared to a more direct approach of using the property labels themselves (or the inverse of property differences) as edge weights for the graph? Is the benefit better generalization, increased robustness to noise, or something else? The authors should briefly discuss this in the manuscript to justify why this more complex learning approach was chosen over a simpler label-based graph construction method.

If property labels are directly used as edge weights (or as inverses of property differences), the model would be unable to make predictions for new, unseen samples, since test molecules lack labels and thus cannot be connected to the existing molecules. By contrast, our approach uses contrastive learning to align the descriptor features with their underlying property similarities in a latent space, and construct graphs for all molecules based on the learned embeddings. This design provides both practical feasibility and improved robustness, as it enables the graph structure to capture meaningful relationships among molecules.

The explanation of this inference mechanism is added in the revised manuscript (**"Methods: Model Inference"** (Pages 29–30)). To further validate the generalization capability and robustness of our approach, additional experiments have conducted: (1) prediction on a novel external dataset of unseen fuels, and (2) comprehensive benchmark evaluation on MoleculeNet datasets. The results demonstrate that our method achieves strong performance in both scenarios, confirming its generalizability and robustness beyond the original fuel property dataset. These new findings are included in **"Discussion"** (Pages 22-23), with further discussions provided in our response to Q2 from Reviewer #2.

4. Description of the Inference Process for New Molecules: This is a crucial point for practical application. The graph built during training includes all training molecules. However, during inference for a novel molecule not in the training graph, how does the model calculate its fused feature $\tilde{x}_{new}$? The "Methods" section (4.3) does not explicitly describe the inference mechanics. This needs to be detailed in the main text or supplementary materials. Clarifying this is essential for the paper's rigor and reproducibility.

During inference, the model first obtains the latent representation $\mathbf{e}_p$ of the new molecule m_p by feeding its original descriptor vector $\mathbf{x}_p$ into the trained embedding network. Then the new molecule is connected to k-NN molecules based on similarities in the learned latent space. With this sparse connection, the fused feature $\tilde{\mathbf{x}}_p$ is computed through the same graph-propagation procedure used during training. For completeness and reproducibility, this inference pipeline is now explicitly described in the revised manuscript (Pages 29–30 Lines 544–558, **"Methods: Model Inference"**).

5. Comprehensiveness of Baseline Model Selection: The comparison against classic ML mod-

els (SVR, MLP, CatBoost), a CNN, and a Vanilla Transformer is good. However, in the field of molecular prediction, Graph Neural Networks (GNNs), particularly those that directly process molecular structural graphs (e.g., built from SMILES or via RDKit atom-bond graphs) such as MPNN, DMPNN, or Attentive FP, are highly relevant and powerful baselines. The lack of comparison against such SOTA GNN baselines is a gap. I strongly recommend adding this comparison or, at a minimum, discussing the rationale behind the current baseline selection and comparing the advantages/disadvantages of the proposed approach versus standard GNNs.

This comment closely aligns with Q3 from Reviewer #1, which also recommends adding more SOTA baselines. Accordingly, **"Results: Performance comparison between LG-Transformer and other baseline models on fuel property prediction"** (Pages 11–14 Lines 215–224) and **"Methods: Baselines"** (Page 30 Lines 570–573) of the revised manuscript has been substantially updated to incorporate six SOTA GNN-based and contrastive learning models as baselines for comparison, including Chemprop [89], ML-QSPR [88], AttentiveFP [85], ACS [84], MolCLR [90], and KANO [91]. These methods operate directly on molecular structural graphs derived from SMILES or atom–bond connectivity and therefore serve as strong benchmarks for evaluating the proposed LG-Transformer. The results in **Tab. R2** (Q3, Reviewer #1) and **Tab. 3** (Page 12) show that LG-Transformer consistently outperforms these SOTA baselines in R^2 accuracy across key fuel properties, demonstrating its advantage in capturing inter-molecular property relationships that conventional structure-based approaches cannot explicitly model.
